# Siderophore‐Linked Ruthenium Catalysts for Targeted Allyl Ester Prodrug Activation within Bacterial Cells

**DOI:** 10.1002/chem.202202536

**Published:** 2022-12-21

**Authors:** James W. Southwell, Reyme Herman, Daniel J. Raines, Justin E. Clarke, Isabelle Böswald, Thorsten Dreher, Sophie M. Gutenthaler, Nicole Schubert, Jana Seefeldt, Nils Metzler‐Nolte, Gavin H. Thomas, Keith S. Wilson, Anne‐Kathrin Duhme‐Klair

**Affiliations:** ^1^ University of York Department of Chemistry Heslington York YO10 5DD UK; ^2^ University of York Department of Biology Heslington Wentworth Way YO10 5DD UK; ^3^ University of York York Structural Biology Laboratory Heslington YO10 5DD UK; ^4^ Anorganische Chemie Ruhr-Universität Bochum Universitätsstraße 150 44801 Bochum Germany

**Keywords:** antibacterials, bio-orthogonal, catalysts, prodrugs, siderophores

## Abstract

Due to rising resistance, new antibacterial strategies are needed, including methods for targeted antibiotic release. As targeting vectors, chelating molecules called siderophores that are released by bacteria to acquire iron have been investigated for conjugation to antibacterials, leading to the clinically approved drug cefiderocol. The use of small‐molecule catalysts for prodrug activation within cells has shown promise in recent years, and here we investigate siderophore‐linked ruthenium catalysts for the activation of antibacterial prodrugs within cells. Moxifloxacin‐based prodrugs were synthesised, and their catalyst‐mediated activation was demonstrated under anaerobic, biologically relevant conditions. In the absence of catalyst, decreased antibacterial activities were observed compared to moxifloxacin versus *Escherichia coli* K12 (BW25113). A series of siderophore‐linked ruthenium catalysts were investigated for prodrug activation, all of which displayed a combinative antibacterial effect with the prodrug, whereas a representative example displayed little toxicity against mammalian cell lines. By employing complementary bacterial growth assays, conjugates containing siderophore units based on catechol and azotochelin were found to be most promising for intracellular prodrug activation.

## Graphical abstract

**Ganging up on bacteria**: Siderophore‐linked ruthenium catalysts were investigated for the activation of an antibacterial prodrug within cells. A fluoroquinolone prodrug was developed and shown to be compatible with the catalysts under micro‐aerobic, biologically relevant conditions. The co‐addition of the catalysts and prodrug to *E. coli* showed a combinative effect, with the dihydroxybenzoic acid‐ and azotochelin‐linked catalysts showing most promise for cellular uptake and thus intracellular prodrug activation.

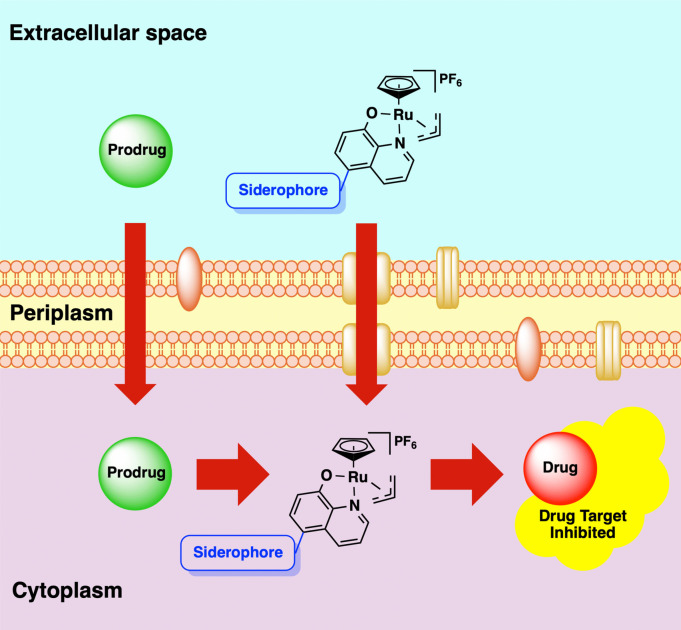

## Introduction

**Figure 1:**
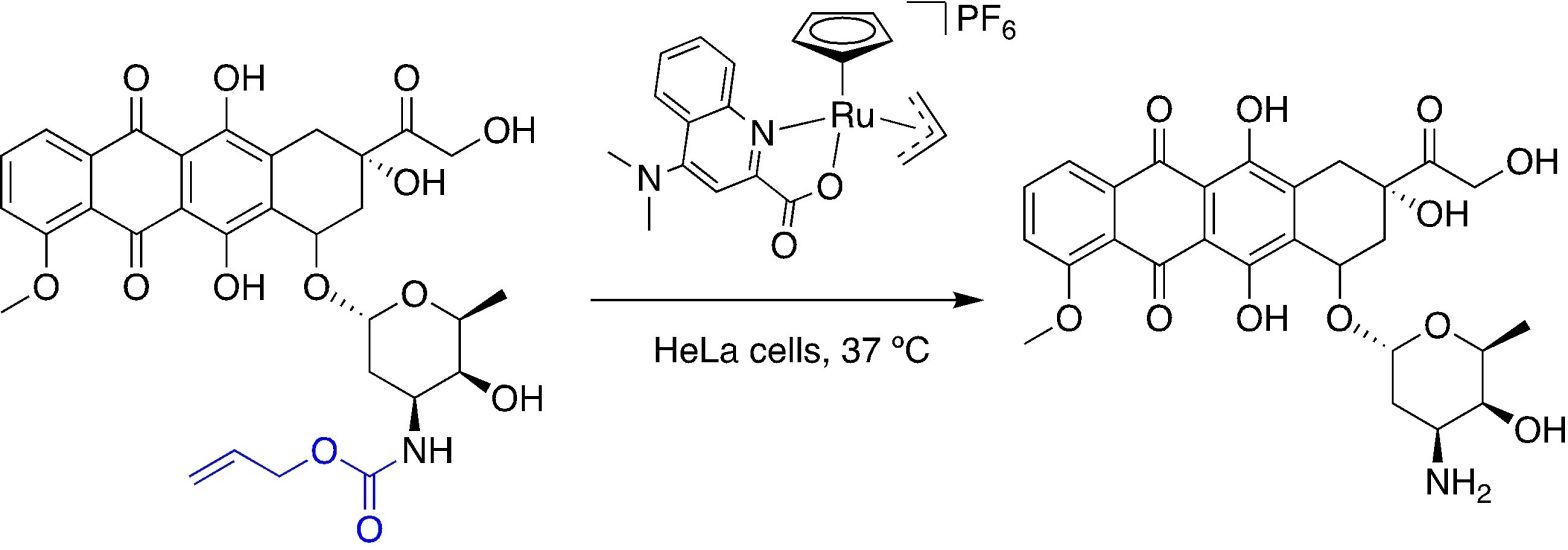
Kitamura‐type catalyst for the activation of the allyl carbamate‐protected anticancer drug doxorubicin under biologically relevant conditions.

Bio‐orthogonal chemistry exploits nonbiological chemical reactions that can occur inside living systems without interfering with native biochemical processes. The integration of abiotic, inorganic catalysts into living cells is a relatively new concept and focuses on the incorporation of organometallic catalysts that include metals such as Ru, Ir, Pd and Au. Despite limitations associated with catalyst toxicity and deactivation by reaction with biological components, numerous advances have been reported.^[1]^ Notably, in 2014 Völker et al.^[2]^ described the use of an organometallic ruthenium catalyst for the deprotection of allyl carbamate‐protected amines under biologically relevant conditions, previously reported by Kitamura et al.^[3]^ for organic synthesis purposes. More recently in 2017, Völker and Meggers optimised catalyst activity for the activation of a prodrug of the anticancer drug doxorubicin in a HeLa cell line (Figure 1).^[4]^

Since their discovery, Kitamura‐type catalysts have been investigated for uses in artificial metalloenzymes^[5]^ as well as alternative prodrug activation strategies.[^6^, ^7^] In 2016, Tomás‐Gamasa et al.^[6]^ reported the development of Kitamura‐type catalysts for reactions within the mitochondria of HeLa cells, by their conjugation to triphenylphosphonium cations. Moreover, the application of an affibody‐conjugated catalyst for bio‐orthogonal prodrug activation toward HER2‐targeted cancer chemotherapy has only been reported very recently by Zhao et al. in 2022.^[8]^ Although the bio‐compatibility of the catalysts against *Escherichia coli* (*E. coli*) bacteria has been investigated by Rubini et al.^[9]^ in 2019, the catalysts have not yet been used for prodrug activation for the treatment of bacterial infections, or conjugated to siderophores as targeting vectors.

**Figure 2:**
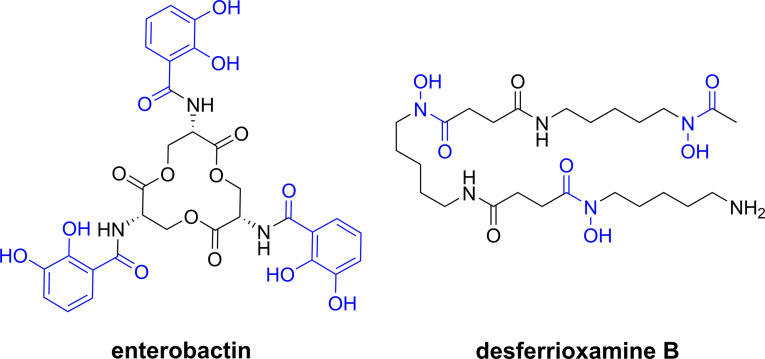
Chemical structure of the hexadentate catechol‐ and hydroxamate‐based siderophores enterobactin and desferrioxamine, with chelator parts in blue.

Siderophores are low‐molecular‐weight compounds secreted by bacteria to acquire iron, which is an essential metal in biology.[^10^, ^11^] In Gram‐negative bacteria, such as *E. coli*, iron‐siderophore complexes are first transported through the outer‐membrane and enter the periplasm, where the complexes are captured by their respective cognate periplasmic binding proteins. The iron‐siderophore complexes are then handed over to ABC transporters for transfer into the cytoplasm through the inner‐membrane. As Gram‐positive bacteria, such as *Bacillus subtilis*, do not possess two membranes, specific cell‐surface receptors transfer the iron‐siderophores complexes directly into the periplasm by using similar ABC transporters.^[7]^ Although siderophores vary in denticity and backbone structure, the iron‐binding components of siderophores are mainly based on three types of bidentate chelator: catecholates, hydroxamates, and α‐hydroxycarboxylates. The most thermodynamically stable iron‐complexes employ hexadentate ligands, for example enterobactin and desferrioxamine B (DFO, Figure 2).

**Figure 3:**
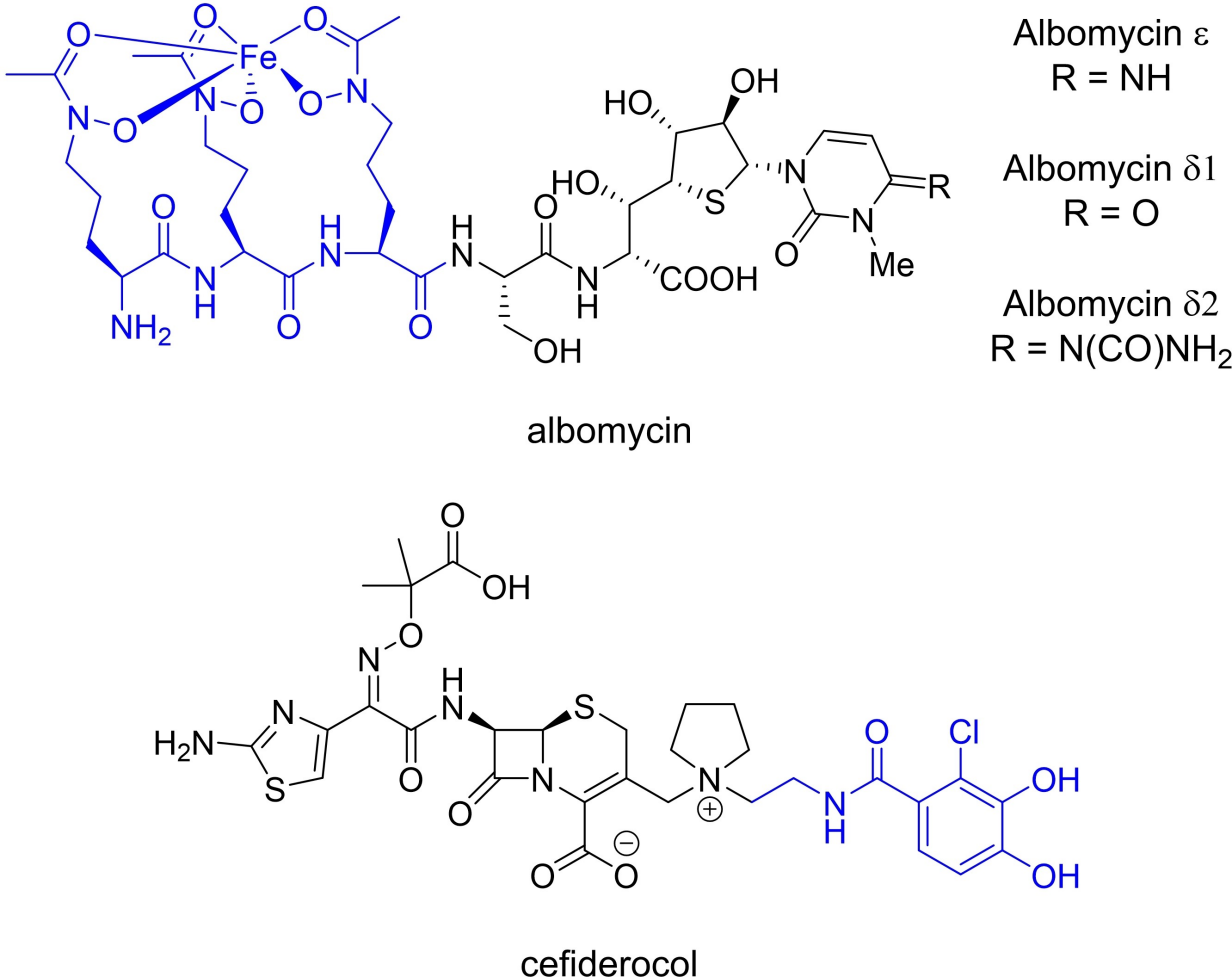
Chemical structures of the sideromycin albomycin and Trojan‐horse antibacterial cefiderocol with the antibacterial warheads in black, siderophore mimics in blue.^[16]^

Some microorganisms produce antibacterial‐siderophore conjugates called sideromycins. Disguised as innocent siderophores, sideromycins are internalised by competing bacteria as their Fe^II^ complexes through siderophore uptake pathways, and the subsequent release of the antibacterial component results in cell death. An example is albomycin produced by *Streptomyces* sp. (Figure 3).^[12]^ These compounds have inspired synthetic analogues which are commonly referred to as “Trojan‐horse” antibacterials due to analogies made with the wooden horse used to conquer the city of Troy in Greek mythology.^[13]^ To date, several Trojan‐horse antibacterials have been investigated for potential pharmaceutical applications however only cefiderocol (FETROJA®) has made it to the clinic, for the treatment of urinary tract infections (Figure 3).[^14^, ^15^]

Herein, we report a conceptionally related Trojan‐horse approach, in which siderophore‐linked derivatives of the Kitamura‐type catalyst have been developed for the activation of antibacterial prodrugs within cells. As bacteria rely on siderophores for the uptake of essential iron(III), whilst eukaryotic cells do not have siderophore transporters, a siderophore‐directed drug‐activation mechanism would select bacterial over eukaryotic cells, thus improving the therapeutic ratio of the parent drugs (antimicrobial vs. toxic effects).

## Results and Discussion

### Design and synthesis of antibacterial prodrugs

**Figure 4:**
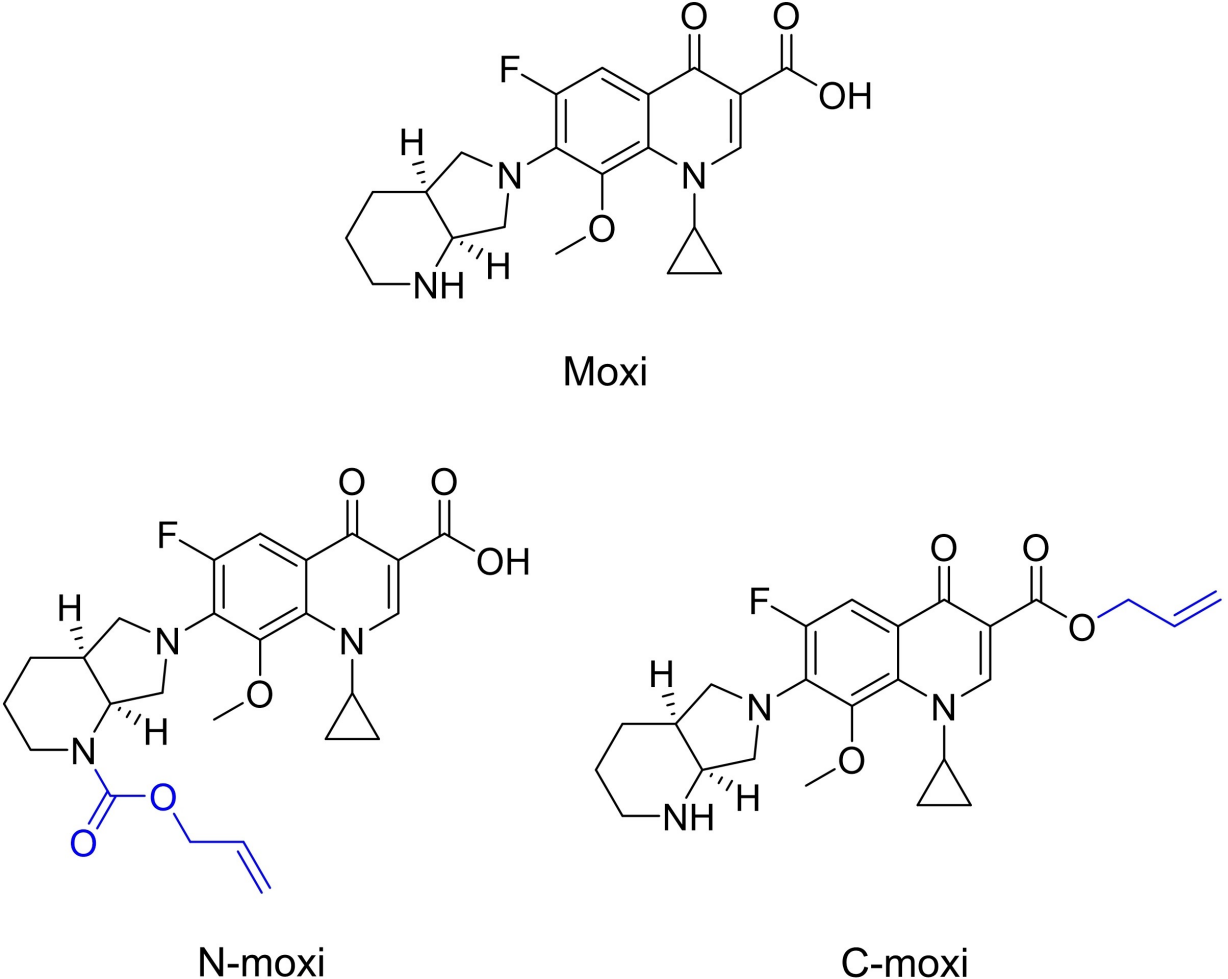
Chemical structures of Moxi, and its prodrugs N‐moxi and C‐moxi.

A suitable antibacterial for prodrug design and synthesis was identified in moxifloxacin (Moxi), a broad spectrum, fourth‐generation quinolone antibacterial that exhibits concentration‐dependent bactericidal activity, Figure 4.^[17]^ Whilst retaining the excellent antibacterial potencies against Gram‐negative bacteria earlier generation fluoroquinolones such as ciprofloxacin possess, Moxi provides expanded Gram‐positive coverage and improved activity against atypical pathogens and under anaerobic conditions.^[18]^ Similar to many quinolones, Moxi contains the essential β‐keto acid motif (C terminus) and a secondary amine (N terminus), and these functional groups can be derivatised to form allyl ester and allyl carbamate‐modified versions, respectively. Both motifs have been previously reported for catalyst‐mediated cleavage using the Kitamura‐type catalysts.

Due to the small size and hydrophilic nature of Moxi, its bacterial uptake is predominantly through porins.^[19]^ It was hypothesised that the derivatisation of Moxi at the N or C terminus to form allyl carbamate or allyl ester prodrugs N‐moxi and C‐moxi (Figure 4), respectively, might enhance bacterial uptake by passive membrane diffusion, mitigating porin deficiency‐associated resistance, due to their greater lipophilicity compared to the parent antibacterial (c log D7.4 data: Moxi=−1.11, N‐moxi=1.27, C‐moxi=1.22). The syntheses of prodrugs N‐moxi and C‐moxi are described in sections 2.1 and 2.2, respectively, of the Supporting Information.

To the best of our knowledge, catalyst‐mediated prodrug activation has not yet been used for allyl ester prodrugs. Compared to modification of the N terminus, modification at the C terminus was expected to provide a more significant toxicity window compared to its activated form, as the β‐keto acid motif constitutes part of the quinolone pharmacophore.^[20]^ Moreover, such prodrugs still possess their secondary amines, which would remain protonated under physiological conditions and thus, might favour passage across the negatively charged outer‐membrane of Gram‐negative bacteria by passive diffusion. In contrast, modification of the C terminus might reduce uptake by porins as coordination to divalent metals such as magnesium is prevented, which have been suggested to improve drug permeability.^[21]^

### Catalysts for prodrug activation

**Figure 5:**
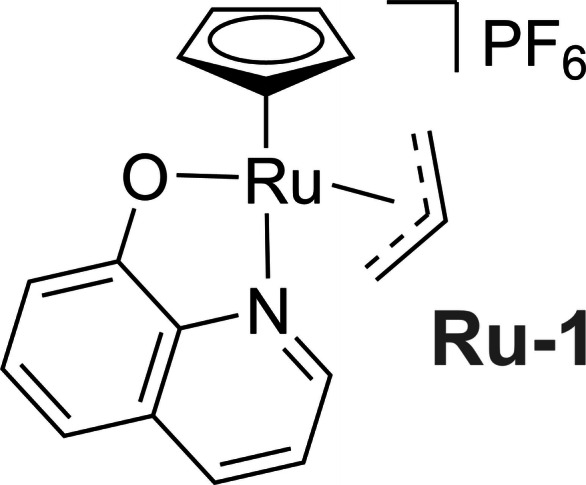
Chemical structure of **Ru**‐**1**.

Following the synthesis of the moxifloxacin prodrugs, their compatibility with Kitamura‐type catalysts for their activation to form Moxi was investigated. Activation kinetics were measured under biologically relevant conditions to mimic the environment inside bacteria, so that data can be used to infer in vivo activity. The optimised hydroxyquinoline‐ligated Kitamura‐type catalysts described by Völker and Meggers in 2017 were chosen for these investigations because of their reported improved reaction kinetics compared to the previous catalyst generations. A specific catalyst, **Ru**‐**1**, was selected and synthesised following a two‐step procedure reported by Völker and Meggers (Figure 5).^[4]^

An HPLC assay was employed to assess prodrug activation kinetics, where both the production of drug and consumption of prodrug was measured based on their corresponding UV‐vis traces. To this end, calibration curves were obtained for the prodrug (C‐moxi) and drug (Moxi), using known concentrations of these components. Analogous studies with N‐moxi were attempted, however the prodrug's poor solubility rendered such experiments unattainable. The experiments were carried out in aqueous MOPS buffer at physiological pH supplemented with 5 mM GSH and 10 % DMSO. For procedural details (including calibration curves) see Section 3 of the Supporting Information.

**Figure 6:**
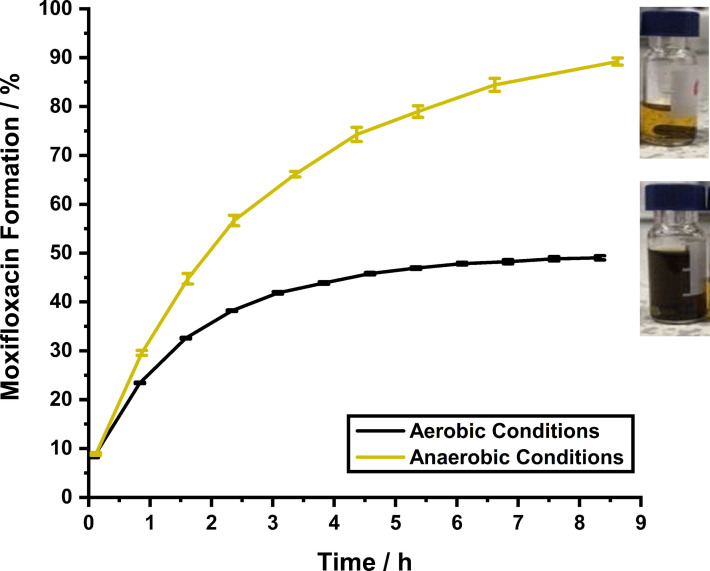
Catalyst‐mediated prodrug activation kinetics using **Ru**‐**1** (10 mol%) to activate C‐moxi (100 μM) in 10 % DMSO in aqueous MOPS buffer (pH 7.4) at room temperature, with 5 mM GSH under an aerobic atmosphere (∼45 % overall yield) and an anaerobic atmosphere (∼90 % overall yield), with corresponding final solution appearances shown next to their corresponding endpoints.

**Figure 7:**
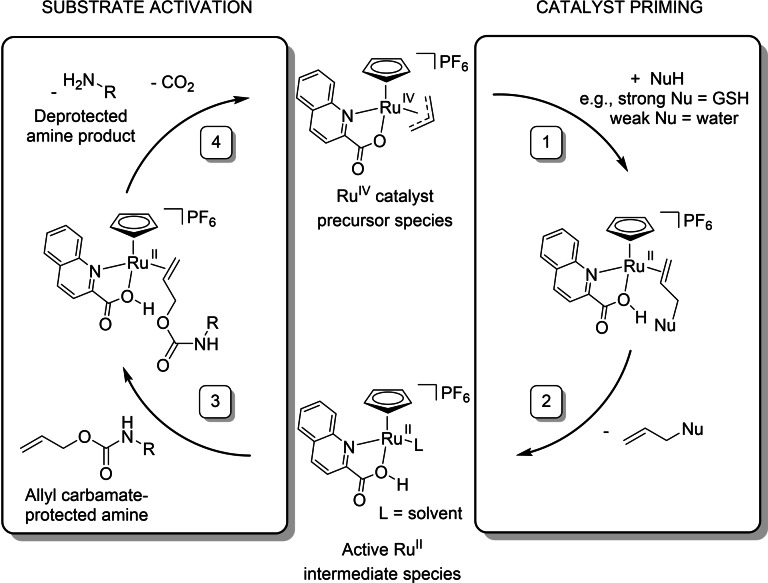
Catalytic mechanism proposed by Völker et al.^[2]^

Although C‐moxi activation occurred at an acceptable initial rate, catalyst activity quickly decreased and was completely lost after ∼8 h (Figure 6, black trace). However, activation kinetics were greatly improved in the absence of molecular oxygen, where 90 % Moxi formation is achieved after ∼8 h. Moreover, **Ru**‐**1** remains active for the entire reaction time. Collectively, these results suggest the poor catalyst activity seen under aerobic conditions is due to O_2_‐mediated catalyst decomposition. This conclusion is supported in the literature, as in 2010 Kiesewetter and Waymouth reported faster catalyst kinetics for the quinaldic series in methanol compared to water, where reactions performed under air gave decreased conversions.^[30]^ Although these investigations did not unambiguously determine the cause for the low conversions for the methanolysis in air, they proposed oxidative decomposition of the active Ru^II^ intermediate formed during the catalyst cycle proposed by Völker et al. (Figure 7).^[2]^

**Figure 8:**
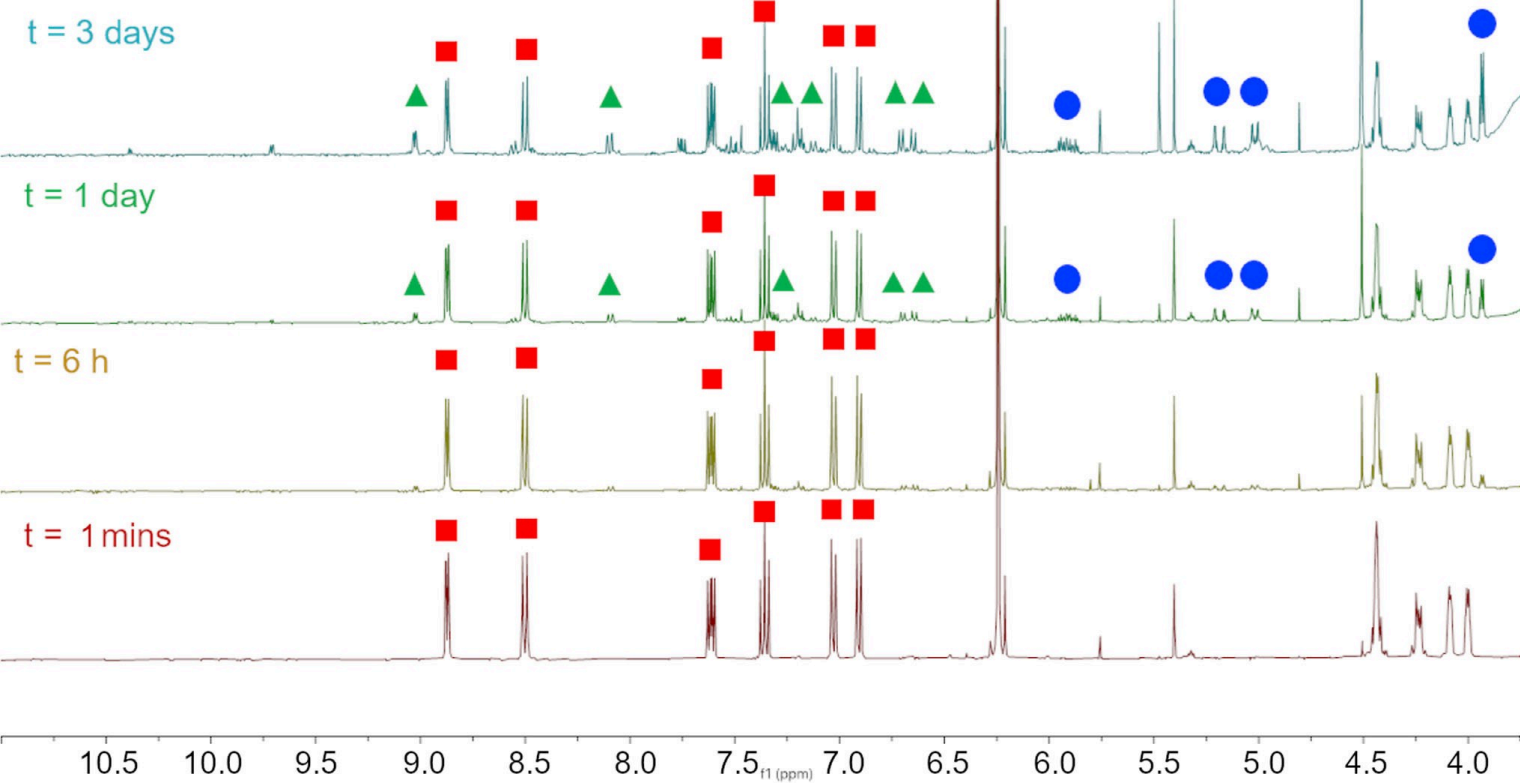
^1^H NMR spectra of **Ru**‐**1** at 10 mM in [D_6_]DMSO after 1 min, 6 h, 1 d and 3 d (▪: resonances assigned to the Ru^IV^ catalyst precursor, ▴: resonances assigned to the corresponding Ru^II^ intermediate, •: resonances assigned to allyl alcohol).

Investigations were undertaken to confirm the sensitivity of the active Ru^II^ intermediate species. By measuring ^1^H NMR spectra of **Ru**‐**1** in [D_6_]DMSO (Figure 8) 1 min, 6 h, 1 d and 3 d after catalyst dissolution, the stability of the Ru^IV^ catalyst precursor species (red square) was confirmed. It remains the major species after 3 d in [D_6_]DMSO under an aerobic atmosphere, with small amounts of the active Ru^II^ intermediate species (green triangle) formed through catalyst priming by water.^[2]^ Moreover, significant amounts of the Ru^IV^ catalyst precursor species remain after one week (Supporting Information, Section 4.1). The formation of the active Ru^II^ intermediate species is identified by the characteristic chemical shift corresponding to cyclopentadienyl ligand at 4.5 ppm and generation of equimolar, allyl alcohol (blue circle).

By promoting formation of the active Ru^II^ intermediate species by dissolution at 2 mM in aqueous MOPS buffer (pH 7.4) using deuterated water with 10 % [D_6_]DMSO, through catalyst priming by nucleophilic water, the sensitivity of this species to molecular oxygen was confirmed (Supporting Information, Section 4.2). Storage under anaerobic conditions preserved this species for 18 h, as a yellow solution, whereas under an aerobic atmosphere, the active species decomposes and precipitates to form a dark‐coloured suspension, like the samples depicted in Figure 6.

### Assessing the prodrug–drug toxicity windows

As the Kitamura‐type catalysts decompose in the presence of oxygen, their medicinal applications in vivo are favoured by low oxygen environments, to prolong catalyst lifetimes. This considered, the treatment of intestinal bacterial infections was identified as a promising target, based on the relatively low oxygen concentrations.^[22]^ *Enterobacteriaceae* are a family of bacteria labelled a “critical priority” by the World Health Organisation due to antibiotic resistance.^[23]^ Gastrointestinal tract infections caused by enteric pathogens affect over 1.7 billion individuals annually, with approximately 2.2 million cases ending in death.^[24]^ Among the leading causes of these infections are Gram‐negative bacteria such as *E. coli*, *Salmonella* sp. and *Campylobacter jejuni*.

An essential property of prodrugs is their reduced activity prior to activation. Therefore, to establish the suitability of the prodrugs N‐moxi and C‐moxi, their antibacterial activity was tested against the relevant, facultative anaerobe, *E. coli* K12 (BW25113) versus Moxi. To mimic the environment of the intestine, assays were carried out under a reduced oxygen atmosphere (2 % O_2_) and at low iron concentrations. Even though some food compounds can be a source of iron, it is generally believed that there is limited access to iron in the gut and therefore iron acquisition is competitive.^[25]^

**Figure 9:**
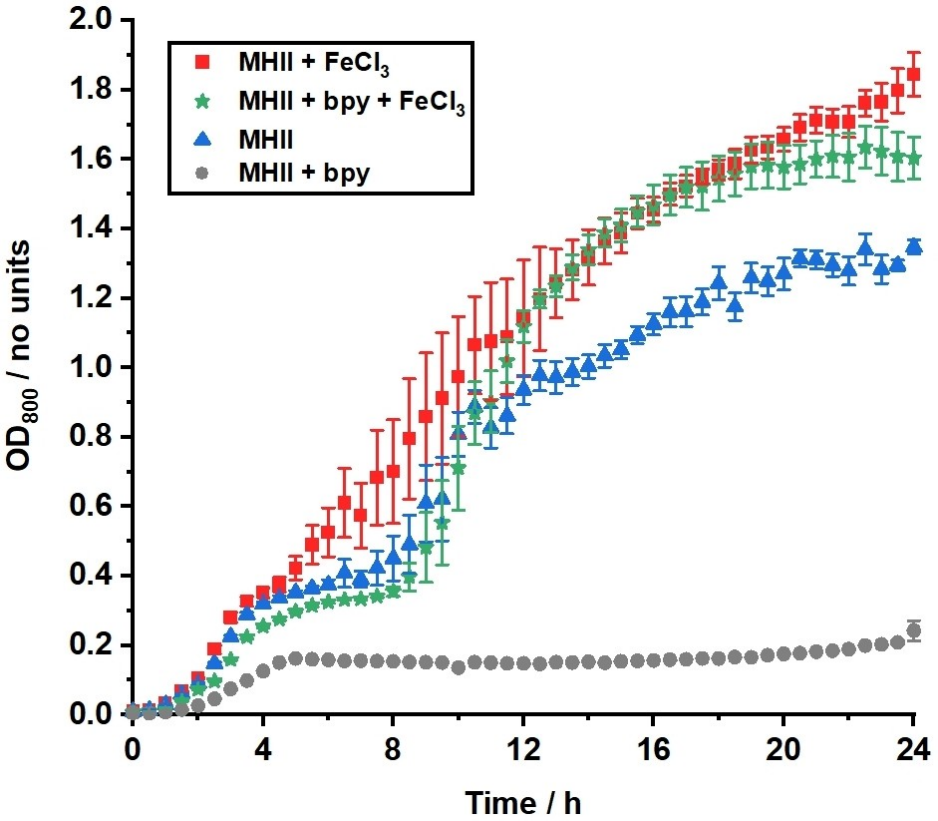
Micro‐aerobic (2 % O_2_) growth of *E. coli* K12 (BW25113) in just MHII (pH 7.4) or MHII supplemented with 200 μM bpy, 100 μM FeCl_3_, and 200 μM bpy + 100 μM FeCl_3_, at 37 °C for 24 h.

A typical iron‐limited growth medium for *E. coli* is desferrated Müller‐Hinton broth II (MHII).[^10^, ^26^] However, the levels of concomitant iron in such media often remain too high to impede bacterial growth. Hence, additional measures are taken, such as the use of Chelex resin and the addition of synthetic chelators such as bipyridine (bpy), during bacterial growth assays to sequester iron.^[27]^ The suitability of MHII supplemented with 200 μM bpy for iron‐limited growth of *E. coli* was assessed using growth curve assays. The optical density (OD) was measured at 800 nm, as the Kitamura‐type catalysts, which will be used in future experiments, typically absorb at 600 nm, the wavelength commonly used to monitor bacterial growth, Figure 9.

**Figure 10:**
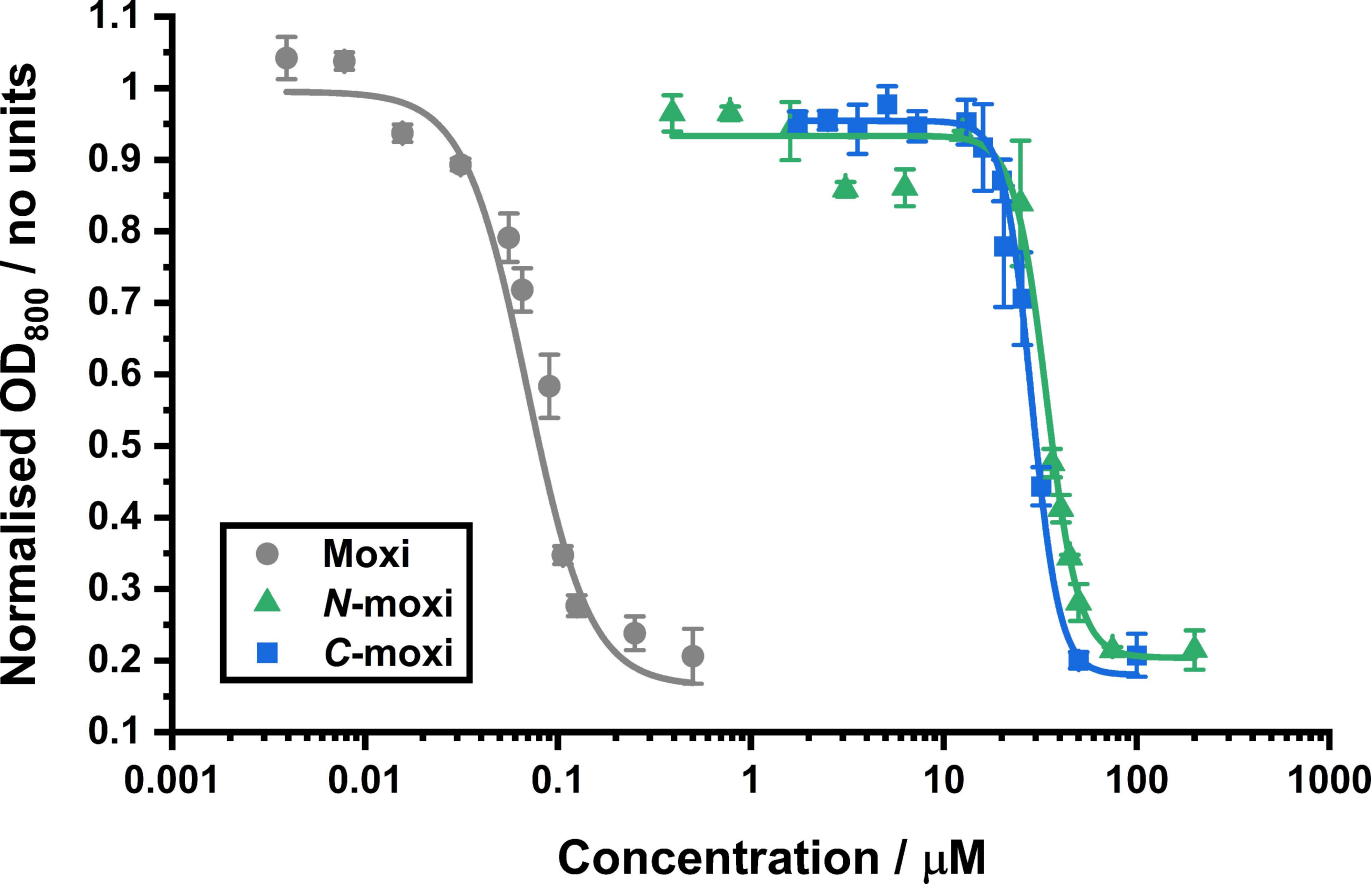
Dosage–response curves of *E. coli* K12 (BW25113) overall growth 24 h after substrate addition. Data are normalised to “no addition” controls, for each of Moxi, N‐moxi and C‐moxi, at their varied substrate concentrations under iron‐limited (MHII supplemented with 200 μM bpy), micro‐aerobic (2 % O_2_) conditions, at 37 °C.

Chelex‐treated MHII alone was insufficient to impose iron limitation, as overall growth is very similar to the positive controls. However, the addition of 200 μM bpy successfully imposes iron limitation. It can be assumed that this is due to bpy coordinating to residual iron and withholding it from the bacteria, as these conditions supplemented with FeCl_3_ rescue bacteria growth. ICP‐MS measurements reported by Sanderson et al. revealed the iron content of Chelex treated MHII is as high as 53 μM, which explains why bpy addition was required.^[28]^ The subsequent antibacterial activity of Moxi and its prodrugs against *E. coli* K12 under the aforementioned conditions is represented as dosage‐response curves (Figure 10).

These data show that under these conditions, the minimum inhibitory concentration (MIC) of the active drug, Moxi, is approximately 300 nM, with no antibacterial activity observed at concentrations lower than approximately 10 nM. Both prodrugs possess significantly larger MIC values, both at approximately 100 μM. No antibacterial activity for N‐moxi or C‐moxi is seen at concentrations lower than approximately 10 μM. This means both prodrugs provide a very similar toxicity window, where only approximately 3 % of each prodrug at its upper nontoxic concentration needs activation to completely inhibit bacterial growth under these conditions.

For prodrug activation inside bacterial cells, it is essential that both reaction components (catalyst and prodrug) are readily taken up by bacteria in sufficient quantities. The bacterial uptake of C‐moxi at 10 μM was evaluated using an incubation experiment, (Supporting Information, Section 6.5). The results suggested ∼16 % internalisation, meaning that for bacteria grown under these conditions, approximately 20 % need activation to observe the maximum inhibitory effect of Moxi (Table S2). Unfortunately, bacterial uptake studies were unsuccessful for N‐moxi due to solubility issues.

### Design and synthesis of siderophore‐linked ruthenium catalysts

**Figure 11:**
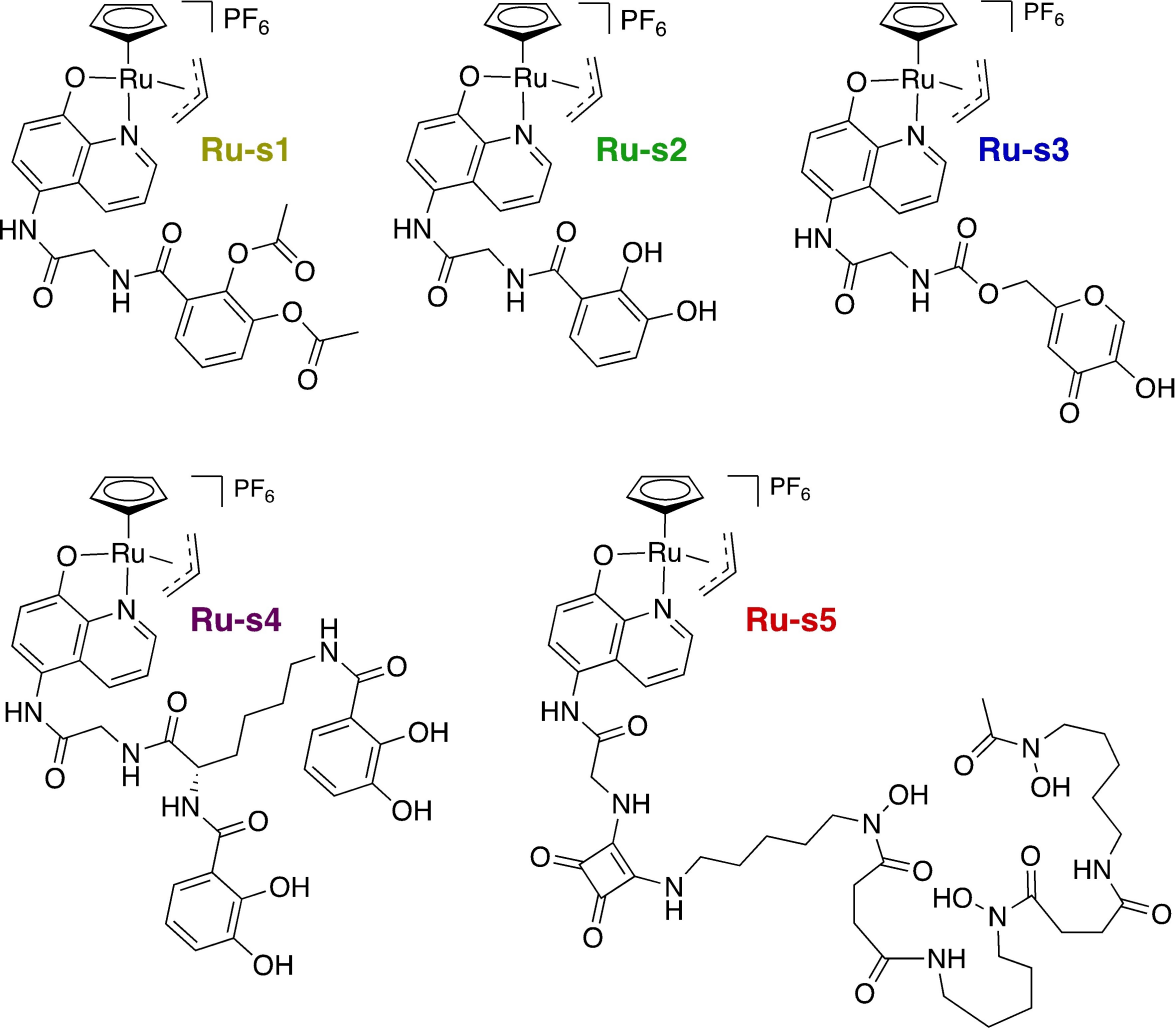
Chemical structures of siderophore‐linked Kitamura‐type catalysts.

In order to facilitate internalisation of the catalyst into bacteria, so that prodrug activation occurs within cells, they were linked to a variety of siderophores and siderophore mimics, which possess various denticities, based on either catechol, hydroxamate or pyranone iron‐binding motifs, including an acetate‐protected catechol that has been shown to auto‐uncage at physiological pH.^[29]^ A total of five siderophore‐catalyst conjugates were synthesised (Figure 11) in which siderophore attachment to the 8‐hydroxyquonline ligand of the Kitamura‐type catalyst was achieved through a glycine linker, accessed in five steps from 5‐nitro‐8‐hydroxyquinoline. All compounds were fully characterised (Supporting Information, Sections 2.4–2.9).

### Prodrug activation kinetics for the siderophore‐linked ruthenium catalysts

**Figure 12:**
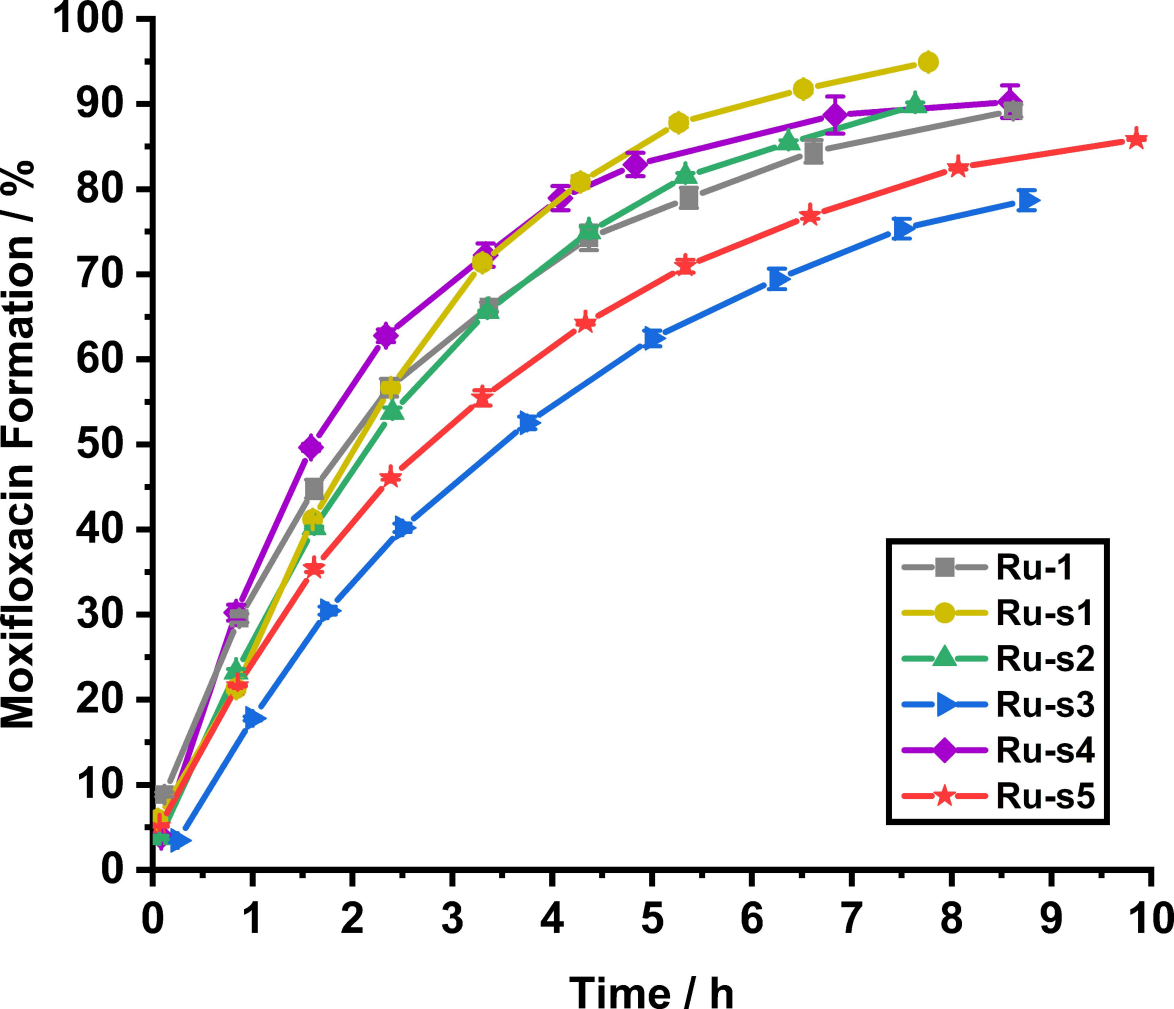
Catalyst‐mediated prodrug activation kinetics in aqueous MOPS buffer (pH 7.4) with 10 % DMSO at room temperature under an anaerobic atmosphere, showing C‐moxi (100 μM) activation to form Moxi for synthesised catalysts **Ru**‐**1**, **Ru**‐**s1**, **Ru**‐**s2**, **Ru**‐**s3**, **Ru**‐**s4** and **Ru**‐**s5** at 10 mol % loading, in triplicate.

The activity of each of the catalyst‐siderophore conjugates for prodrug activation under biologically relevant conditions was determined. To assess the impact of siderophore attachment on catalytic prodrug activation kinetics, reaction rates and conversions were compared to those achieved by the control catalyst, **Ru**‐**1**. To this end, HPLC kinetics were employed under an anaerobic atmosphere. Kinetic data for Moxi formation are shown for each catalyst in Figure 12. Each catalyst's C‐moxi consumption and corresponding Moxi formation kinetics are shown in Section 3.5 of the Supporting Information.

The results confirm that every catalyst‐siderophore conjugate activates the prodrug C‐moxi to form Moxi under these conditions. Most catalysts perform as well as or better than the control catalyst **Ru**‐**1**, with yields of approximately 90 % Moxi formation and 10 % C‐moxi remaining after 8 h. The exceptions to this include the pyranone‐ and DFO‐conjugated catalysts **Ru**‐**s3** and **Ru**‐**s5**, respectively. The activity of **Ru**‐**s5** might be explained by the relatively large size of their siderophore compared to the those of the other catalysts, as it probably sterically hinders the active ruthenium metal centre more, thereby kinetically perturbing its reaction with nucleophiles for catalyst priming and prodrug during substrate activation.

### Effect of siderophore‐linked catalysts on bacterial growth and cytotoxicity

**Figure 13:**
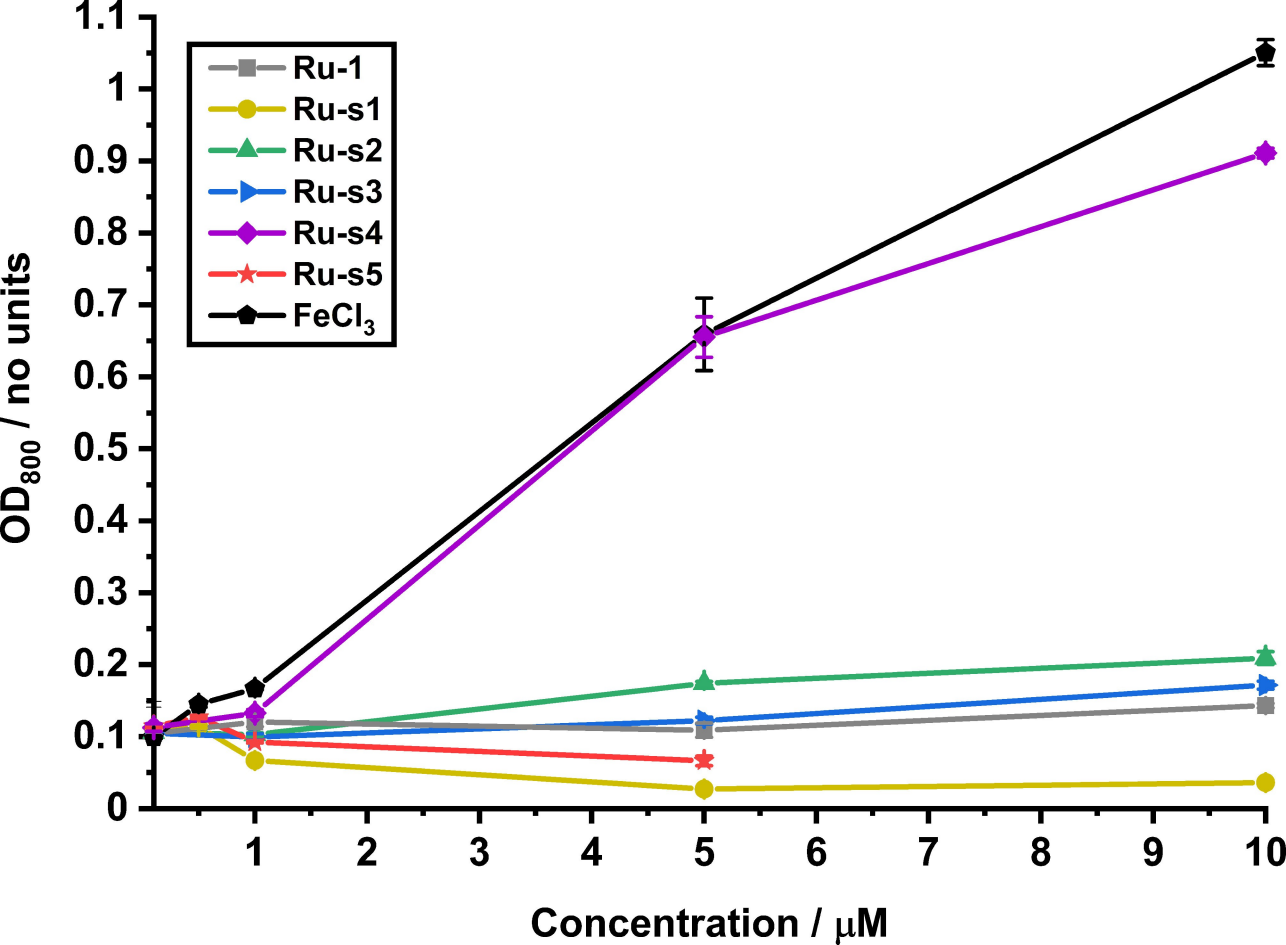
Dose‐response curves of *E. coli* K12 (BW25113) overall growth. Overall growth at 24 h, for each of **Ru**‐**1**, **Ru**‐**s1**, **Ru**‐**s2**, **Ru**‐**s3**, **Ru**‐**s4**, **Ru**‐**s5** and the iron control (FeCl_3_) at their varied substrate concentrations under iron‐limited (MHII supplemented with 200 μM bpy), micro‐aerobic (2 % O_2_) conditions, in at least technical triplicate.

The antibacterial activity of each catalyst‐siderophore conjugate was tested between 0.1–10 μM against *E. coli* K12 (BW25113) grown in iron‐limited MHII under micro‐aerobic conditions. The growth conditions used are the same as those reported for the determination of the prodrug MICs, apart from the preparation of the samples inside an anaerobic chamber. The data from these experiments are represented as dosage‐response curves for each catalyst‐siderophore conjugate in Figure 13. The procedural details for these experiments can be found in the Supporting Information, Section 6.6.3.

These data show that “no addition” controls grow to an OD_800_ of ∼0.1 and that the Kitamura‐type catalyst control **Ru**‐**1** has no observable effect on overall bacterial growth. In contrast, a positive effect is observed for FeCl_3_, with overall growth increasing tenfold at 10 μM compared to “no addition”. Interestingly, the azotochelin conjugate **Ru**‐**s4** improves bacterial growth at upper concentrations at a similar rate to the iron control, thus suggesting that this conjugate improves the availability of iron to the bacteria. No observable effect on bacterial growth was observed for the pyranone conjugate **Ru**‐**s3** however overall growth is doubled by the addition of the catechol conjugate **Ru**‐**s2** at 10 μM. In contrast, the acetate‐protected catechol **Ru**‐**s1** and DFO **Ru**‐**s5** conjugates gave overall growth reductions at concentrations above 1 μM and 500 nM, respectively. The antibacterial activity of **Ru**‐**s5** can be attributed to the iron‐sequestering ability of DFO, as toxicity is reversed in excess iron (data not shown).

In order to strengthen claims that **Ru**‐**s2** and **Ru**‐**s4** might facilitate iron‐uptake into bacteria, the iron concentration of the media after the addition of each catalyst at 10 μM was determined by ICP‐MS in order to discount iron contamination (Supporting Information, Section 6.5). The value for each of the catalyst‐siderophore conjugates showed no [^56^Fe] values greater than that of the “no‐addition” control suggesting no significant iron contamination occurs on the addition of these catalysts.

Additionally, the cytotoxicity of **Ru**‐**s4**, Moxi and C‐moxi was tested against the human cervical cancer cell line HeLa and noncancerous fibroblast cell line GM5657, performing MTT‐based proliferation assays with an incubation time of 48 h. For all tested compounds, the IC_50_ values were above the tested concentration range (0.05–100 μM). Especially the results for **Ru**‐**s4** are encouraging for the future application of this biorthogonal system as the Ru‐containing catalysts do not seem to be toxic to mammalian cells.

### Antibacterial activity of siderophore‐linked catalysts with prodrug co‐addition

**Figure 14:**
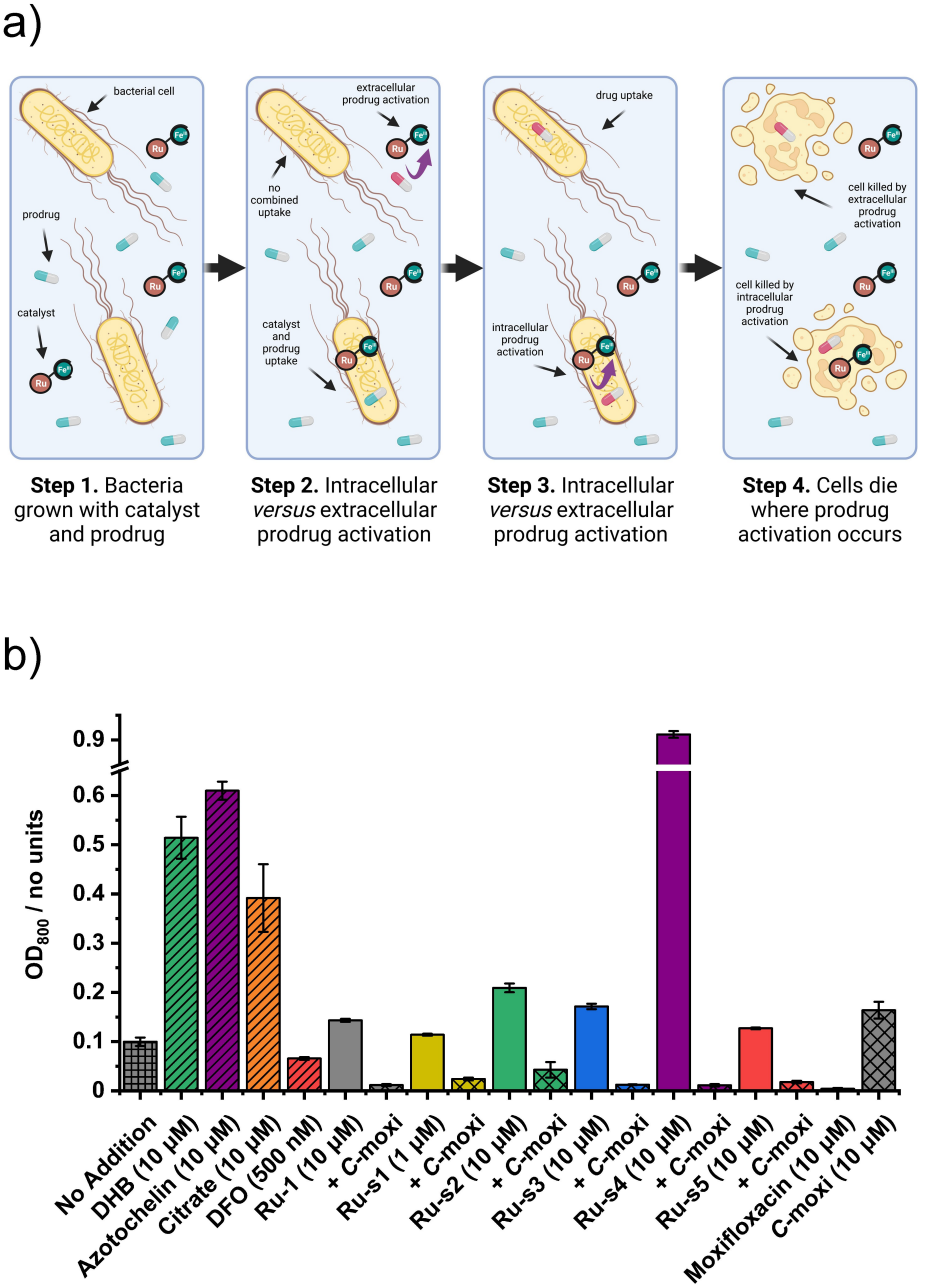
Catalyst–prodrug co‐addition. a) Schematic representation of antibacterial activity by intra‐ and extracellular prodrug activation during co‐incubation of catalyst and prodrug. Created with biorender.com. b) Overall growth of *E. coli* K12 (BW25113) grown under iron‐limited (MHII supplemented with 200 μM bpy), micro‐aerobic (2 % O_2_) conditions after 24 h with each catalyst–siderophore conjugate at its upper nontoxic concentration with and without C‐moxi (10 μM), and controls for siderophores, C‐moxi and Moxi, in at least technical triplicate.

Now that the upper nontoxic concentrations have been determined for each of the prodrugs and synthesised catalyst‐siderophore conjugates, their combined antibacterial activities at these concentrations were evaluated. Each catalyst was added at their upper nontoxic concentration with C‐moxi, also at its upper nontoxic concentration (10 μM). The data from these experiments are represented in the form of a bar chart, where each bar represents the overall growth of bacteria 24 h after the addition of the respective combination of components, Figure 14. Each co‐addition is listed adjacent to its “just catalyst‐siderophore conjugate” control. Additional controls include tests with **Ru**‐**1**, “just prodrug” (C‐moxi) and drug (Moxi), and siderophore controls including DFO, 2,3‐dihydroxybenzoic acid (DHB), azotochelin, and citrate.

Firstly, it is important to examine the growth effect of the siderophore controls. The siderophores DHB, azotochelin and citrate improve growth relative to the “no addition” control. The growth enhancement observed for azotochelin is similar to that provided by **Ru**‐**s4** at the same concentration. In contrast, DHB provides significant growth enhancements but neither of the catechol conjugated catalysts **Ru**‐**s1** and **Ru**‐**s2** provide this to the same extent, although **Ru**‐**s2** does double overall growth. The siderophore DFO does not improve growth, confirming it is a poor siderophore for *E. coli*, explaining the similar lack of growth improvement for the DFO conjugate **Ru**‐**s5**.

The co‐addition experiments for each catalyst result in antibacterial activity comparable to that following Moxi addition at 10 μM, as the overall growth of bacteria diminishes significantly compared to the “no addition” control and their corresponding “just catalyst” controls. In fact, the growth enhancement observed for the azotochelin conjugate **Ru**‐**s4** is completely reversed following prodrug addition. These results suggest that the catalysts do indeed activate the prodrug C‐moxi to form Moxi and that their stability in the presence of bacteria is sufficient to produce a toxic concentration of Moxi. These experiments do not, however, establish the location of catalyst‐mediated prodrug activation, as both are added to the bacterial growth media simultaneously.

### Evaluating the bacterial uptake of the siderophore‐linked catalysts

The 2017 World Health Organization list of priority pathogens is dominated by Gram‐negative bacteria, many of which are fluoroquinolone resistant.^[14]^ Gram‐negative bacteria, however, are a challenging target for the development of much needed new antibiotics, due to the permeability barrier imposed by their protective cell envelope, in particular the outer membrane decorated with its lipopolysaccharides. As siderophore‐based targeting vectors have the potential to overcome this permeability barrier, we proceeded to investigate *E. coli* as a representative Gram‐negative organism with well‐characterised iron‐siderophore transporters.

The uptake of the prodrug C‐moxi into *E. coli* K12 (BW25113) was inferred through prodrug uptake studies. Therefore, if bacteria are preloaded with the prodrug and the prodrug remains within cells, any antibacterial activity observed by the addition of synthesised catalyst‐siderophore conjugates relative to their corresponding “no C‐moxi” controls, implies intracellular prodrug activation and thus, catalyst uptake. Consequently, prodrug incubation experiments were used to evaluate the bacterial uptake of the catalyst‐siderophore conjugates. To encourage uptake, *E. coli* K12 (BW25113) cells were incubated with C‐moxi during the exponential growth phase. Additional MIC data were obtained for C‐moxi and Moxi after addition at this time, represented as dosage response curves in Section 6.6.4 and Figure S22 in the Supporting Information. The MIC of Moxi is approximately 25 μM whereas for C‐moxi, no antibacterial activity is observed up to its solubility limit at 500 μM.

**Figure 15:**
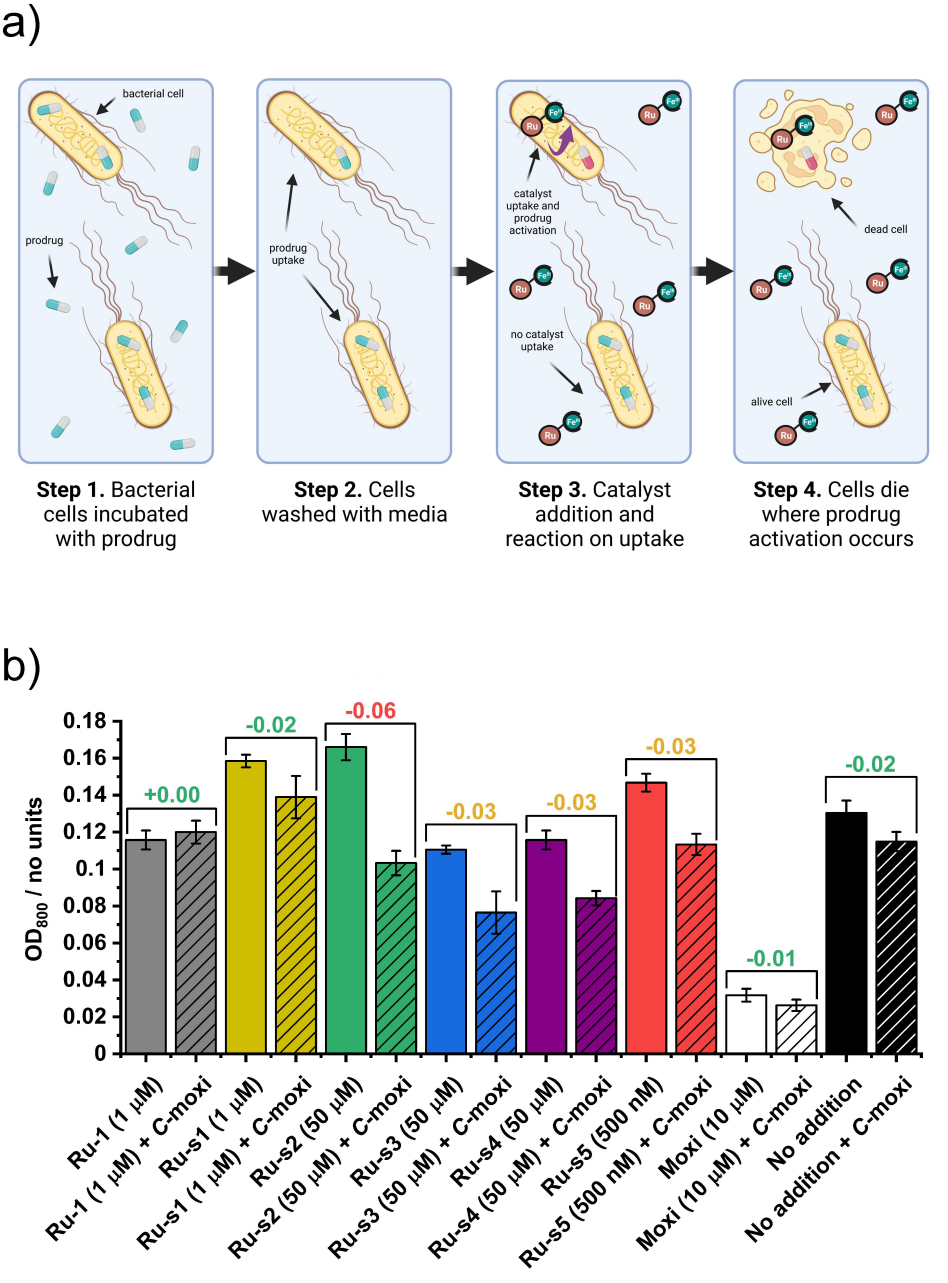
Evaluating cellular uptake. a) Schematic representation of antibacterial activity as consequence of intracellular prodrug activation, following sequential prodrug incubation, cellular uptake, washing and catalyst addition steps. Created with biorender.com. b) Overall growth of *E. coli* K12 (BW25113) under iron‐limited (MHII supplemented with 200 μM bpy), micro‐aerobic (2 % O_2_) conditions, 18 h after C‐moxi (hatched bars) or DMSO (solid bars) incubation and subsequent addition of substrate. Substrates include **Ru**‐**1**, **Ru**‐**s1**, **Ru**‐**s2**, **Ru**‐**s3**, **Ru**‐**s4** and **Ru**‐**s5** at their upper, nontoxic concentrations and controls: Moxi (white) and “no addition” (black). The difference in overall growth between each incubation with and without C‐moxi incubation, is highlighted in a bracket over the corresponding bar charts. Each incubation was carried out in technical triplicate, and each subsequent substrate addition in at least technical triplicate.

For prodrug incubation experiments, the bacteria were therefore incubated with C‐moxi at 500 μM for 15 min at 37 °C. This culture was then spun down, the pellet isolated and resuspended in fresh MHII, where this process was repeated twice to ensure no C‐moxi remained in the media, and that any prodrug still present was inside or associated with the cells. Each catalyst was added at its upper nontoxic concentration, which sometimes varied from previous experiments due to addition in the exponential phase. The overall growth of these bacteria after 18 h was recorded and is represented as a bar chart in Figure 15. For each catalyst, there is also a “no C‐moxi incubation” control, where the cells were instead incubated with plain DMSO. Since the catalysts perform prodrug activation at a similar rate, the greater reduction in growth following C‐moxi incubation compared to DMSO implies better catalyst uptake. Controls include “no addition” and Moxi addition, with their corresponding DMSO and C‐moxi incubations. It is important to also take into consideration the growth improvements provided by the conjugates and thus data are interpreted by evaluating the growth difference between incubations.

Expectedly, data show no significant difference between incubations with subsequent no catalyst addition. There is also no significant difference between incubations with subsequent Moxi addition, as Moxi and C‐moxi do not work in an additive manner. The overall growth is lower for Moxi as the added concentration is above its MIC value. There is no difference between incubations with subsequent **Ru**‐**1** addition indicating it is not internalised in sufficient amounts to observe an additive antibacterial effect due to C‐moxi activation. Although there is a small difference between incubations for **Ru**‐**s1**, this is not statistically significant. However, there are observable differences between incubations for the remaining catalyst‐siderophore conjugates **Ru**‐**s2**, **Ru**‐**s3**, **Ru**‐**s4** and **Ru**‐**s5**. This is especially the case for **Ru**‐**s2** where overall growth is diminished by ∼40 %, which reveals the monocatechol conjugate **Ru**‐**s2** as the most promising catalyst conjugate for prodrug activation within these bacteria. Interestingly, this result is in line with the fact that the most successful Trojan‐horse antibacterial conjugate to date, cefiderocol, also utilises a monocatechol siderophore unit. The growth inhibition after C‐moxi and **Ru**‐**s2** co‐addition, however, is not as inhibitory as the Moxi control on its own. As both the catalyst and the prodrug must be co‐located in sufficient concentrations for activation to occur, this observation is not unexpected and points towards a need for improved uptake of either C‐moxi or the catalyst‐siderophore conjugate, or higher catalyst turnovers. Yet, these initial results are promising and merit further investigations to this end, whilst also prompting studies into the opportunities that this approach offers for the targeting of bacterial cells over mammalian cells.

## Conclusions

A series of siderophore‐linked ruthenium catalysts, **Ru**‐**s1**—**Ru**‐**s5**, with a variety of different chelator motifs and denticities has been synthesised and characterised. Each example was shown to activate the synthesised prodrug C‐moxi (derived from the potent antibacterial moxifloxacin Moxi) under biologically relevant conditions. It was found that the ruthenium‐based catalysts perform better under anaerobic conditions due to the sensitivity of the active Ru^II^ intermediate species to molecular oxygen. Hence, bacteria grown under a micro‐aerobic atmosphere were targeted. Due to the poor solubility profile of N‐moxi, only C‐moxi was taken forward into bacterial assays, where the prodrug was found to be ∼300 times less toxic than the parent antibacterial, Moxi. A combinative effect was observed for each catalyst conjugate with C‐moxi when both were added at their individual nontoxic concentrations.

A number of bacterial growth assays were employed to evaluate the bacterial uptake of each catalyst. Considering the enhanced growth of iron‐limited bacteria in the presence of the catechol (**Ru**‐**s2**) and especially the azotochelin conjugate (**Ru**‐**s4**), it was inferred that these conjugates facilitated iron uptake and therefore were internalised. Subsequent bacterial growth assays with C‐moxi incubation implied that each of the catalyst‐siderophore conjugates was internalised in *E. coli* to some extent; however, none was comparable to the positive control Moxi. The bacterial growth was substantially diminished compared to the non‐siderophore control (**Ru**‐**1**) for each of the conjugates apart from **Ru**‐**s1**. **Ru**‐**s2** performed best in these assays, as bacterial growth was reduced by ∼40 % compared to “no‐prodrug” incubation. Furthermore, the low cytotoxicities of Moxi, C‐moxi and especially **Ru**‐**s4** with IC_50_ values greater than 100 μM for two tested mammalian cell lines are promising results for the future application of such biorthogonal systems in human tissue.

Overall, these investigations demonstrate the potential that catalyst‐siderophore conjugates have for prodrug activation strategies; however, there is still much development required before this approach can be considered for the targeted treatment of bacterial infections. The sensitivity of the catalyst to molecular oxygen limits its applicability to micro‐aerobic and anaerobic environments. Moreover, to obtain targeted bacterial uptake, further studies are required to probe the compatibility of the siderophore conjugates with the targeted membrane receptors required for internalisation. As each bacterial species has its own system of siderophores that it can produce and use, in principle a siderophore‐directed drug‐activation mechanism could allow the targeting of specific pathogenic strains based on the type of siderophore used, thereby avoiding the indiscriminate exposure that spreads resistance. Future work will investigate the catalyst‐siderophore conjugates with Gram‐positive bacteria, where published results can be expected soon.

## Experimental Section

All experimental data, synthesis and assay protocols are provided in the Supporting Information.

## Conflict of interest

The authors declare no conflict of interest.

1

## Supporting information

As a service to our authors and readers, this journal provides supporting information supplied by the authors. Such materials are peer reviewed and may be re‐organized for online delivery, but are not copy‐edited or typeset. Technical support issues arising from supporting information (other than missing files) should be addressed to the authors.

Supporting Information

## Data Availability

The data that support the findings of this study are available in the supplementary material of this article.
