# Supplementary material for: Siderophore‐Linked Ruthenium Catalysts for Targeted Allyl Ester Prodrug Activation within Bacterial Cells
Source: Chemistry. 2022 Dec 21;29(8):e202202536. doi: 10.1002/chem.202202536 (PMC10108276; doi:10.1002/chem.202202536)
Supplement: Supplementary file 1 — Supporting Information [file CHEM-29-0-s001.pdf]

Chemistry–A European Journal

Supporting Information

Siderophore-Linked Ruthenium Catalysts for Targeted Allyl Ester Prodrug Activation within Bacterial Cells

James W. Southwell, Reyme Herman, Daniel J. Raines, Justin E. Clarke, Isabelle Böswald, Thorsten Dreher, Sophie M. Gutenthaler, Nicole Schubert, Jana Seefeldt, Nils Metzler-Nolte, Gavin H. Thomas, Keith S. Wilson, and Anne-Kathrin Duhme-Klair*

Contents

1. General Remarks ________ *3*

1.1. Materials ________ 3

1.2. Instrumentation ________ 3

2. Synthesis ________ *5*

2.1. The Synthesis of *N*-moxi ________ 5

2.2. The Synthesis of *C*-moxi ________ 7

2.3. The Synthesis of Ru-1 ________ 12

2.4. The Synthesis of Glycine-linked 8-alloxyquinoline ________ 15

2.5. The Synthesis of Ru-s1 ________ 21

2.6. The Synthesis of Ru-s2 ________ 26

2.7. The Synthesis of Ru-s3 ________ 31

2.8. The Synthesis of Ru-s4 ________ 38

2.9. The Synthesis of Ru-s5 ________ 50

3. Catalyst-mediated Prodrug Activation Kinetics using HPLC ________ *59*

3.1. Sample Conditions ________ 59

3.2. Column Conditions ________ 59

3.3. Calibration Curves ________ 60

3.4. Kinetic Run Procedure ________ 61

3.4.1. Aerobic Conditions ________ 61

3.4.2. Anaerobic Conditions ________ 62

3.5. Additional Data for *C*-moxi Activation by Catalyst-Siderophore Catalyst Conjugates ________ 62

4. Catalyst Stability Studies ________ *66*

4.1. Oxygen Sensitivity of the Ru(IV) catalyst Precursor Species ________ 66

4.2. Oxygen Sensitivity of the Active Ru(II) Intermediate Species ________ 67

5. Calculated Octanol-Water Coefficients ________ *68*

6. Bacteria Growth Assays 69

6.1. Media Recipes 69

6.1.1. Minimal M9 Media 69

6.1.2. Müller-Hinton II Broth 69

6.2. ICP-MS Measurements for the Iron and Ruthenium Content of Media 70

6.3. Bacterial Strains 71

6.4. Iron-starved Bacteria 71

6.5. Prodrug Uptake Assay 71

6.5.1. Incubation Preparation 72

6.5.2. HPLC Sample Preparation 72

6.5.3. HPLC Column Conditions 73

6.5.4. Calibration Curve 73

6.5.5. Data Processing 74

6.6. Plate Assays 75

6.6.1. Probing Iron-limitation of Müller-Hinton Broth II 75

6.6.2. Antibacterial Activity of Moxifloxacin and Prodrugs 76

6.6.3. Antibacterial Activity of Catalyst-Siderophore Conjugates and their Co-addition with Prodrug *C*-moxi 78

6.6.4. Antibacterial Activity of Moxifloxacin and *C*-moxi during the Exponential Growth Phase 79

6.6.5. Antibacterial Activity of Catalyst-Siderophore Conjugates following Bacteria Incubation with *C*-moxi During the Exponential Growth Phase 81

7. Cytotoxicity against Mammalian Cells 84

7.1. Cell viability assays for Moxi, *C*-Moxi and Ru-s4 84

8. References 86

1. General Remarks

1.1. Materials

All materials were obtained from commercial suppliers (Acros, Alfa-Aesar, Fisher Chemicals, Fisher Scientific, Fluka, Sigma-Aldrich, Tokyo Chemical Industry) and used as supplied unless otherwise stated.

1.2. Instrumentation

^{1}H, $^{13}C\{^{1}H\}$, DEPT 135, HSQC, COSY, HMBC and NOESY NMR spectra were recorded on Jeol EX and ES 400 MHz instruments (^{1}H NMR 400 MHz, ^{13}C NMR 101 MHz), unless stated otherwise. Alternative instruments included a Bruker AV500b (^{1}H NMR 500 MHz, ^{13}C NMR 126 MHz) and a AVIIIHD600 (^{1}H NMR 600 MHz, ^{13}C NMR 151 MHz). Multiplicity abbreviations are as follows; s for singlet, d for doublet, t for triplet, q for quartet qn for quintet, sx for sextet, sp for septet, dd for double doublet, td triple doublet, ddd for doublet of doublet of doublets, m for multiplet, br for broad and app for apparent. NMR assignments were aided by various combinations of DEPT 135, HSQC, COSY, HMBC and NOESY experiments, where required.

Electrospray ionisation mass spectrometry (ESI-MS) was recorded on a Bruker compact® TOF mass spectrometer and Liquid Injection Field Desorption Ionization (LIFDI), on a Waters GCT Premier TOF mass spectrometer by Mr. K. Heaton, Dr. R. Cercola and Mr. A. Lopez. Error is reported in two forms: ‘error (ppm)’ which describes closeness of the measured m/z value to that of the theoretical, and ‘mSigma’ which describes the similarity of the measured isotope patten to that of the theoretical.

Elemental analysis was carried out by Dr. G. McAllister on an Exeter CE-440 elemental analyser and results are within ± 0.5 %, unless stated otherwise.

Analytical HPLC was performed using a Shimadzu Prominence LC 20AD setup, with C18 column and SPD M20A diode array detector under the direction of Ms. A. Dixon and Dr. S. Hicks.

TLC was performed on Merck silica gel 60 F_{254} aluminium backed plates and visualised under a Chromato-vue Model CC-10 UV lamp, unless otherwise stated. Electronic absorption spectra were recorded on a Shimadzu UV-1800 spectrophotometer at ambient temperature.

Infrared (ATIR) spectra were recorded on a Bruker ALPHA II – Platinum FT-IR Spectrometer with Platinum Diamond-ATR QuickSnap Sampling Module, at ambient temperature.

Melting points were determined using a Stuart Scientific SMP3 melting point apparatus.

2. Synthesis

2.1. The Synthesis of *N*-moxi

1-Cyclopropyl-6-fluoro-8-methoxy-4-oxo-7-{1-[(prop-2-en-1-yloxy) carbonyl]-octahydro-1*H*-pyrrolo[3,4-b]pyridin-6-yl}-1,4-dihydro-quinoline-3-carboxylic acid, *N*-moxi

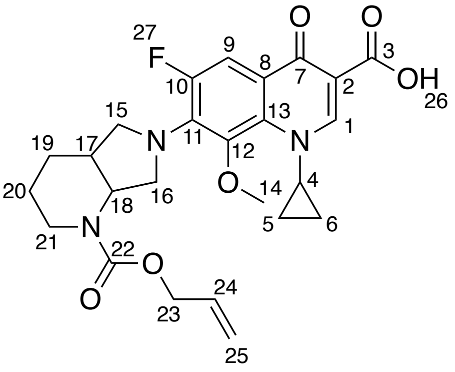

$C_{25}H_{28}FN_3O_6$, 485.512 g mol^{-1}

1-Cyclopropyl-6-fluoro-8-methoxy-4-oxo-7-{1-[(prop-2-en-1-yloxy) carbonyl]-octa-hydro-1*H*-pyrrolo[3,4-b]pyridin-6-yl}-1,4-dihydro-quinoline-3-carboxylic acid was prepared based on a procedure from the literature.[1]

To a green/yellow suspension of moxifloxacin hydrochloride (50 mg, 0.114 mmol) in dry dichloromethane (3 mL) at room temperature, was added 1,8-bis(dimethyl)naphthalene (24 mg, 0.114 mmol). After cooling the reaction mixture to 0 °C, DIPEA (20 μL, 0.114 mmol) and allyl chloroformate (13 μL, 0.114 mmol) were added to give a yellow, clear solution and left stirring for 18 h at room temperature. After this time, dichloromethane (25 mL) was added and the organic layer was washed with a mixture of 1 M HCl (aq) (10 mL) and brine (10 mL), x3. Solvent was removed *in vacuo*, to give a pale yellow solid. The resulting solid was then triturated in PET and filtered to yield the title compound as an off-white white solid. The resulting pale yellow solid was purified *via* column chromatography, eluting with dichloromethane : MeOH (19:1) and then triturated with Et_2O, to yield the title compound as an off-white solid.

Yield: 54 mg, 111 μmol, 97%

Rf: 0.70 in dichloromethane : MeOH (9:1)

HRMS (ESI+):

Calc. for $[C_{25}H_{28}FN_3O_6]+H^+$ = 486.2035 Found = 486.2043 (-1.6 ppm error, 0.7 mSigma)

Calc. for $[C_{25}H_{28}FN_3O_6]+Na^+$ = 508.1854 Found = 508.1864 (-1.8 ppm error, 1.9 mSigma)

^{1}H NMR: (400 MHz, $CDCl_3$) δ: 14.98 (s, 1H-**26**), 8.75 (s, 1H-**9**), 7.76 (d, *J* = 14.0 Hz, 1H-**1**), 6.00 – 5.89 (m, 1H-**24**), 5.30 (dd, *J* = 17.0, 2.0 Hz, 1H-**25 (trans)**), 5.22 (dd, *J* = 10.0, 1.5 Hz, 1H-**25 (cis)**), 4.82 (brs, 1H**-18**), 4.66 – 4.57 (m, 2H-**23**), 4.18 – 4.06 (m, 2H**-15/16+21**), 4.02 – 3.94 (m, 1H-**4**), 3.93 – 3.85 (m, 1H**-15/16**), 3.57 (s, 3H-**14**), 3.42 (s, 1H**-15/16**), 3.28 (d, *J* = 10.0 Hz, 1H**-15/16**),f 3.02 – 2.89 (m, 1H**-21**), 2.35 – 2.23 (m, 1H-**17**), 1.90 – 1.71 (m, 2H-**19**), 1.61 – 1.42 (m, 2H-**20**), 1.34 – 1.21 (m, 1H-**5**), 1.18 – 1.01 (m, 2H-**5/6**), 0.88 – 0.76 (m, 1H-**6**).

^{13}C NMR: (101 MHz, $CDCl_3$) δ: 176.9-**7**, 167.2-**3**, 155.8-**10,** 152.6 (d, *J* = 19.0 Hz, -**11**) 149.9-**1**, 137.3 (d, *J* = 10.5 Hz-**12**), 134.5-**13**, 133.0-**24**, 118.9 (d, *J* = 9.0 Hz, **-8**), 117.9-**25**, 108.2 (d, *J* = 24.0 Hz, **-9**), 107.8-**2**, 66.5-**23**, 61.3-**14**, 56.6 (d, *J* = 8.5 Hz, -**15**), 52.7-**18**, 49.0 (d, *J* = 6.0 Hz, **-16**), 40.5-**4**, 39.7-**21**, 35.7-**17**, 25.3-**19**, 24.2-**20**, 10.7-**5**, 8.6-**6**.

^{19}F NMR: (376 MHz, $CDCl_3$) δ: -121.1 (s, 1F-**27**)

IR ATIR (cm^{-1}): 3083 w (Aromatic/Alkene C-H stretch), 2922 m (Alkane C-H stretch), 2852 m (Alkane C-H stretch), 1727 s (C=O stretch), 1694 s (C=O stretch), 1617 s (Alkene C=C stretch), 1508 m (Aromatic C=C stretch).

Elemental Analysis:

Calculated for $[C_{25}H_{28}FN_3O_6 \cdot 0.15\ C_4H_{10}O]$: %C 61.94, %H 6.04, %N 8.40
Measured for $[C_{25}H_{28}FN_3O_6 \cdot 0.15\ C_4H_{10}O]$: %C 61.84, %H 5.89, %N 8.27

Melting Point: 90 – 92 °C

UV-vis Spectroscopy:

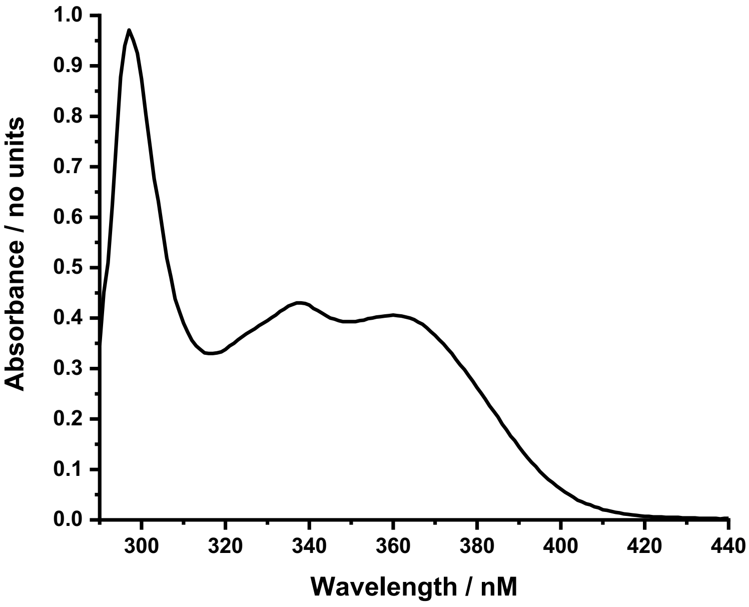

Figure S 1 UV-vis spectrum, 100 μM in 10% DMSO in aqueous MOPS buffer (pH 7.4).

2.2. The Synthesis of *C*-moxi

7-{1-[(*Tert*-butoxy)carbonyl]-octahydro-1*H*-pyrrolo[3,4-b]pyridin-6-yl}-1-cyclopropyl-6-fluoro-8-methoxy-4-oxo-1,4-dihydroquinoline-3-carboxylic acid, 1

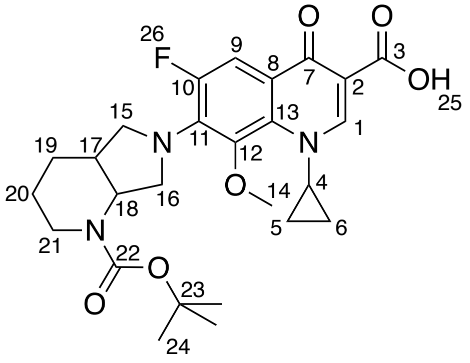

$C_{26}H_{32}FN_3O_6$, 501.555 g mol^{-1}

To a green/yellow suspension of moxifloxacin hydrochloride (423 mg, 0.966 mmol) in a dioxane : water mixture (1:1, 10 mL) at room temperature, was added 1M NaOH (aq) (2 mL) to yield a pale green solution, and then di-*tert*-butyl dicarbonate (1.26 mmol, 274 mg). The resulting solution was left stirring at room temperature for 3 d. After this time, dioxane was removed *in vacuo* and to the remaining aqueous solution was added dichloromethane (50 mL). The organic layer was washed with 0.1 M formic acid (aq) (30 mL, x3) and solvent removed *in vacuo*, to yield the title compound, as a beige solid.

Yield: 464 mg, 0.925 mmol, 96%

Rf: 0.83 in dichloromethane : MeOH : DIPEA (90:10:1)

HRMS (ESI+):

Calc. for $[C_{26}H_{32}FN_3O_6]+H^+$ = 502.2348 Found = 502.2338 (1.9 ppm error, 3.9 mSigma)

Calc. for $[C_{26}H_{32}FN_3O_6]+Na^+$ = 524.2167 Found = 524.2160 (1.5 ppm error, 9.1 mSigma)

^{1}H NMR: (400 MHz, $CDCl_3$) δ: 15.00 (br s, 1H-**25**), 8.76 (s, 1H-**1**), 7.78 (d, *J* = 13.5 Hz, 1H-**9**), 4.78 (br s, 1H-**18**), 4.12 – 4.03 (m, 2H-**15**+**21**), 4.03 – 3.95 (m, 1H-**4**), 3.86 (td, *J* = 10.5, 3.0 Hz, 1H-**16**), 3.56 (s, 3H-**14**), 3.38 (br s, 1H-**16**), 3.30 – 3.20 (m, 1H-**15**), 2.87 (t, J = 11.5 Hz, 1H-**21**), 2.33 – 2.19 (m, 1H-**17**), 1.86 – 1.72 (m, 2H-**19**+**20**), 1.47 (m, 11H-**19**+**20**+**24**), 1.35 – 1.23 (m, 1H-**5**), 1.18 – 1.00 (m, 2H-**5**+**6**), 0.87 – 0.77 (m, 1H-**6**).

^{19}F NMR: (376 MHz, $CDCl_3$) δ: -120.9 (d, *J* = 10.0 Hz, 1F-**26**).

NMR spectra are in agreement with literature reports.[2]

Prop-2-en-1-yl 7-{1-[(*tert*-butoxy)carbonyl]-octahydro-1*H*-pyrrolo [3,4-b]pyridin-6-yl}-1-cyclopropyl-6-fluoro-8-methoxy-4-oxo-1,4-di-hydroquinoline-3-carboxylate, 2

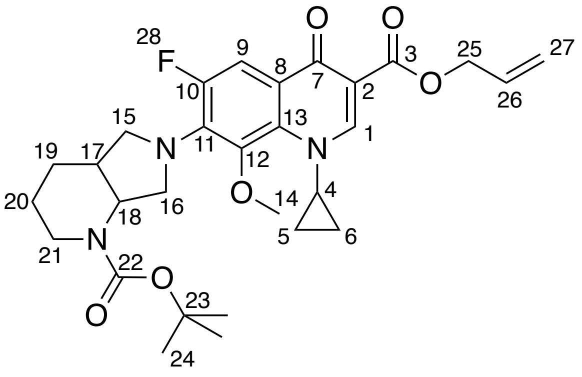

$C_{29}H_{36}FN_3O_6$, 541.620 g mol^{-1}

To a solution of 7-{1-[(*tert*-butoxy)carbonyl]-octahydro-1H-pyrrolo[3,4-b]pyridin-6-yl}-1-cyclopropyl-6-fluoro-8-methoxy-4-oxo-1,4-dihydroquinoline-3-carboxylic acid (445 mg, 0.890 mmol) in a dry DMF (15 mL) at room temperature, was added K_2CO_3 (245 mg, 1.77 mmol) and allyl bromide (152 µL, 1.77 mmol). The resulting solution was heated to 80 °C and left stirring at this temperature for 18 h. After this time, solvent was removed *in vacuo* and the residue formed dissolved in dichloromethane (40 mL). The organic layer was washed with water (30 mL, x3) the solvent removed *in vacuo* to yield the title compound as an orange/yellow foam.

Yield: 454 mg, 0.838 mmol, 94%

Rf: 0.63 in EtOAc

HRMS (ESI+):

Calc. for $[C_{29}H_{36}FN_3O_6]+H^+$ = 542.2667 Found = 542.2667 (-1.0 ppm error, 1.7 mSigma)

Calc. for $[C_{29}H_{36}FN_3O_6]+Na^+$ = 564.2480 Found = 564.2487 (-1.2 ppm error, 2.1 mSigma)

Calc. for $[C_{29}H_{36}FN_3O_6]+K^+$ = 580.2220 Found = 580.2233 (-2.3 ppm error, 33.3 mSigma)

^{1}H NMR: (400 MHz, $CDCl_3$) δ: 8.54 (s, 1H-**1**), 7.82 (d, *J* = 14.0 Hz, 1H-**9**), 6.12 – 5.97 (m, 1H-**26**), 5.46 (dd, J = 17.0, 1.5 Hz, 1H-**27(trans)**), 5.26 (dd, *J* = 9.5, 1.5 Hz, 1H-**27(cis)**), 4.83-4.81 (m, 2H-**25**), 4.76 (br s, 1H-**18**), 4.10 – 3.97 (m, 2H-**15**+**21**), 3.93 – 3.76 (m, 2H-**4**+**16**), 3.55 (s, 3H-**14**), 3.34 (br s, 1H-**16**), 3.25 – 3.13 (m, 1H-**15**), 2.93 – 2.81 (m, 1H-**21**), 2.23 (h, *J* = 5.5 Hz, 1H-**17**), 1.85 – 1.71 (m, 2H-**19**+**20**), 1.30 – 1.15 (m, 1H-**5**), 1.12 – 0.93 (m, 2H-**5**+**6**), 0.86 – 0.71 (m, 1H-**6**).

^{19}F NMR: (376 MHz, $CDCl_3$) δ: 123.2 (s, 1F-**28**).

NMR spectra are in agreement with literature reports.[1]

Prop-2-en-1-yl 1-cyclopropyl-6-fluoro-8-methoxy-7-{octahydro-1*H*-pyrrolo[3,4-b]pyridin-6-yl}-4-oxo-1,4-dihydroquinoline-3-carboxy-late, *C*-moxi

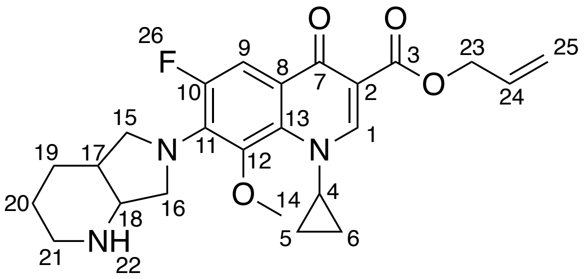

$C_{24}H_{28}FN_3O_4$, 441.503 g mol^{-1}

To a solution of *N*-boc-*C*-moxi (437 mg, 0.81 mmol) in a dichloromethane (15 mL) at room temperature, was added trifluoroacetic acid (1 mL) and left stirring at this temperature for 18 h. After this time, solvent was removed *in vacuo* and the residue formed dissolved in $CHCl_3$ (30 mL). The organic layer was washed with saturated $NaHCO_3$ (aq) (20 mL, x3), and the solvent removed *in vacuo* to yield the title compound as a light brown solid.

Yield: 350 mg, 793 µmol, 98%

HRMS (ESI+):

Calc. for $[C_{24}H_{28}FN_3O_4]+H^+$ = 442.2137 Found = 442.2126 (2.4 ppm error, 1.9 mSigma)

Calc. for $[C_{24}H_{28}FN_3O_4]+Na^+$ = 464.1956 Found = 464.1947 (2.4 ppm error, 6.8 mSigma)

^{1}H NMR: (400 MHz, $CDCl_3$) δ: 8.53 (s, 1H-**1**), 7.77 (d, *J* = 14.5 Hz, 1H-**9**), 6.04 (ddt, *J* = 16.0, 10.5, 5.5 Hz, 1H-**24**), 5.46 (ddd, *J* = 17.0, 2.0 Hz, 1H-**25(trans)**), 5.25 (d, *J* = 10.5 Hz, 1H-**25(cis)**), 4.86 – 4.76 (m, 2H-**23**), 3.99 – 3.82 (m, 3H-**4**+**15**+**16**), 3.54 (s, 3H-**14**), 3.44 – 3.34 (m, 2H-**15**+**16**), 3.31 (m, 1H-**18**), 3.04 (dt, *J* = 11.5, 3.5 Hz, 1H-**21**), 2.73 – 2.69 (td, *J* = 11.5, 3.0 Hz,1H-**21**), 2.29 (tt, *J* = 8.5, 4.0 Hz, 1H-**17**), 1.90 – 1.62 (m, 3H-**19**+**20**), 1.58 – 1.45 (m, 1H-**20**), 1.29 – 1.11 (m, 1H-**5**), 1.11 – 0.94 (m, 2H-**5**+**6**), 0.87 – 0.73 (m, 1H-**6**).

^{13}C NMR: (101 MHz, $CDCl_3$) δ: 172.9 (d, *J* = 2.5 Hz, -**7**), 165.7-**3**, 153.5 (d, *J* = 248.0 Hz-**10**), 150.6-**1**, 140.8 (d, *J* = 7.0 Hz, -**12**), 136.2 (d, *J* = 11.0 Hz, -**11**), 133.5 (d, *J* = 1.0 Hz, -**13**), 132.6-**24**, 121.5 (d, *J* = 8.0 Hz, **8**), 118.2-**25**, 109.6-**2**, 108.7 (d, *J* = 24.0 Hz, -**9**), 65.4-**23**, 60.9-**14**, 58.3 (d, *J* = 6.0 Hz, -**16**), 56.7 (d, *J* = 2.0 Hz, -**18**), 52.4 (d, *J* = 6.5 Hz, -**15**), 45.0-**21**, 39.6-**4**, 36.9 (d, *J* = 1.0 Hz, -**17**), 23.5-**19**, 22.1-**20**, 10.1-**6**, 8.7-**5**.

^{19}F NMR: (376 MHz, $CDCl_3$) δ: 122.9 (d, *J* = 14.0 Hz, 1F-**26**).

NMR spectra are in agreement with literature reports.[1]

IR ATIR (cm^{-1}): 3091 m (Aromatic/Alkene C-H stretch), 3009 w (Aromatic C-H stretch), 2922 m (Alkane C-H stretch), 2871 m (Alkane C-H stretch), 2852 m (Alkane C-H stretch), 1688 s (C=O stretch), 1607 m (Alkene C=C stretch), 1480 m (Aromatic C=C stretch).

Elemental Analysis:

Calculated for $[C_{24}H_{28}FN_3O_4 \cdot 0.05\ CHCl_3 \cdot 0.05\ H_2O]$: %C 64.43, %H 6.33, %N 9.37
Measured for $[C_{24}H_{28}FN_3O_4 \cdot 0.05\ CHCl_3 \cdot 0.05\ H_2O]$: %C 64.64, %H 6.15, %N 9.07

Melting Point: 139 – 141 °C

UV-vis Spectroscopy:

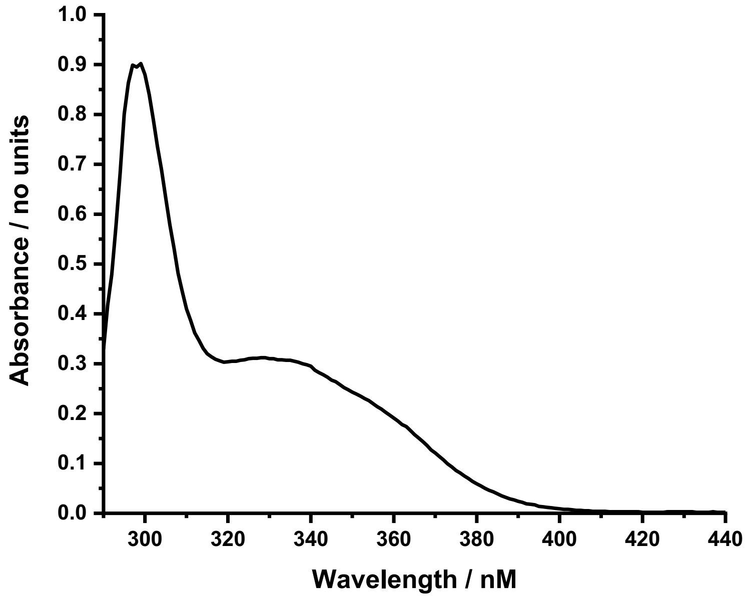

Figure S 2 UV-vis spectrum, 50 μM in 10% DMSO in aqueous MOPS buffer (pH 7.4).

2.3. The Synthesis of Ru-1

8-(Prop-2-en-1-yloxy)quinoline, 3

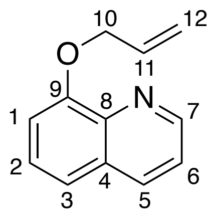

$C_{12}H_{11}NO$, 185.226 g mol^{-1}

8-(Prop-2-en-1-yloxy)quinoline was prepared following a procedure from the literature.[3]

Yield: 284 mg, 1.53 mmol, 74%

Rf: 0.3 in EtOAc : PET (1:3)

HRMS (ESI+):

Calc. for $[C_{12}H_{11}NO]+H^+$ = 186.0913 Found = 186.0910 (1.8 ppm error, 1.5 mSigma)

^{1}H NMR: (400 MHz, $CDCl_3$)) δ: 8.94 (dd, *J* = 4.0, 1.5 Hz, 1H-**7**), 8.11 (dd, *J* = 8.5, 1.5 Hz, 1H-**5**), 7.46 – 7.36 (m, 3H-**6+3+2**), 7.06 (dd, *J* = 7.5, 1.0 Hz, 1H-**1**), 6.26 – 6.16 (m, 1H-**11**), 5.46 (dd, *J* = 17.5, 1.5 Hz, 1H-**12(trans)**), 5.33 (dd, *J* = 10.5, 1.5 Hz, 1H-**12(cis)**), 4.87 (dt, *J* = 5.5, 1.5 Hz, 2H-**10**).

^{13}C NMR: (126 MHz, $CDCl_3$) δ: 154.4-**9**, 149.5-**7**, 140.5-**8**, 136.0-**5**, 133.3-**11**, 129.6-**4**, 126.7-**6/3/2**, 121.7-**6/3/2**, 119.8-**6/3/2**, 118.5-**12**, 109.3-**1**, 69.9-**10**.

NMR spectra are in agreement with literature reports.[3]

Ru-1

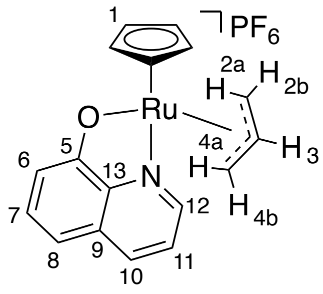

$C_{17}H_{16}F_6NOPRu$, 496.355 g mol^{-1}

Ru-1 was prepared following a procedure from the literature.[3]

Yield: 16 mg, 32.2 μmol, 56%

HRMS (ESI+):

Calc. for $[C_{17}H_{16}NORu]^+$ = 352.0270 Found = 352.0272 (0.7 ppm error, 8.6 mSigma)

^{1}H NMR: (400 MHz, acetone-*d$_6$*)) δ: 8.96 (dd, *J* = 5.0, 1.0 Hz, 1H-**12**), 8.49 (dd, *J* = 8.5, 1.0 Hz, 1H-**10**), 7.60 (dd, *J* = 8.5, 5.0 Hz, 1H-**11**), 7.39 (t, *J* = 8.0 Hz, 1H-**7**), 7.04 (d, *J* = 8.0 Hz, 1H-**6/8**), 6.94 (dd, *J* = 8.0, 0.5 Hz, 1H-**6/8**), 6.30 (s, 5H-**1**), 4.77 – 4.56 (m, 2H-**3+2a/4a**), 4.48 – 4.38 (m, 1H-**2a/4a**), 4.38 – 4.31 (m, 1H-**2b/4b**), 4.22 – 4.16 (m, 1H-**2b/4b**).

^{13}C NMR: (101 MHz, acetone-*d$_6$*) δ: 170.2-**5**, 156.2-**12**, 146.5-**9**, 140.5-**10**, 131.6-**13**, 131.1-**6/7**, 124.5-**11**, 116.6-**6/7**, 112.8-**8**, 99.4-**3**, 97.0-**1**, 69.8-**2/4**, 63.4-**2/4**

^{19}F NMR: (376 MHz, acetone-*d$_6$*) δ: -72.4 (d, *J* = 707.5 Hz, 6F).

^{31}P NMR: (162 MHz, acetone-*d$_6$*) δ: -143.6 (p, *J* = 707.5 Hz, 1P).

NMR spectra are in agreement with literature reports.[3]

IR ATIR (cm^{-1}): 3124 m (Aromatic/Alkene C-H stretch), 3107 w (Aromatic/Alkene C-H stretch), 1585 m (Alkene C=C stretch), 1572 m (Alkene C=C stretch), 1502 m (Aromatic C=C stretch).

Elemental Analysis:

Calculated for [$C_{17}H_{16}F_6NOPRu$ • 0.35 CH_2Cl_2 • 0.5 $C_4H_{10}O$]: %C 40.30, %H 3.48, %N 2.59

Measured for [$C_{17}H_{16}F_6NOPRu$ • 0.35 CH_2Cl_2 • 0.5 $C_4H_{10}O$]: %C 40.54, %H 3.20, %N 2.57

UV-vis Spectroscopy:

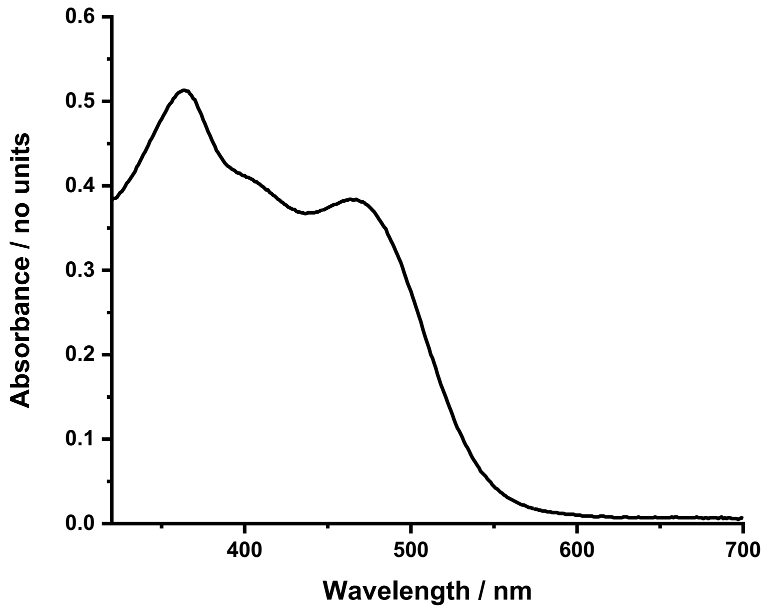

Figure S 3 UV-vis spectrum, 200 μM in DMSO.

2.4. The Synthesis of Glycine-linked 8-alloxyquinoline

Each siderophore-linked catalyst was accessed through this intermediate.

5-Azaniumyl-8-hydroxyquinolin-1-ium dichloride, 4

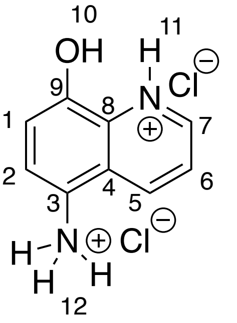

$C_9H_{10}ON_2Cl$, 233.09 g mol^{-1}

5-Azaniumyl-8-hydroxyquinolin-1-ium dichloride was prepared following a procedure from the literature.[6]

Yield: 361 mg, 1.55 mmol, 98%

Rf: 0.67 in dichloromethane : MeOH (9:1)

HRMS (ESI+):

Calc. for $[C_9H_{10}ON_2Cl]+H^+$ = 161.0709 Found = 161.0708 (0.9 ppm error, 5.1 mSigma)

^{1}H NMR: (400 MHz, D_2O) δ: 9.12 – 9.01 (m, 2H-**5+7**), 8.21 – 8.07 (m, 1H-**6**), 7.71 (d, *J* = 8.0 Hz, 1H-**2**), 7.38 (d, *J* = 8.4 Hz, 1H-**1**).

^{13}C NMR: (101 MHz, D_2O) δ: 147.5-**9**, 143.9-**5/7**, 140.7-**5/7**, 129.4-**8**, 125.0-**2**, 123.9-**4**, 123.0-**6**, 119.8-**3**, 115.7-**1**.

2-{[(*Tert*-butoxy)carbonyl]amino}acetyl 2-{[(*tert*-butoxy)carbonyl]-amino}acetate, 5

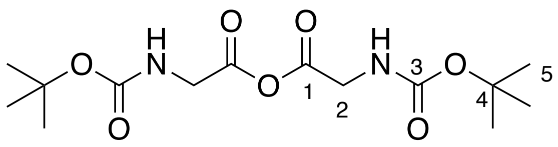

$C_{14}H_{24}N_2O_7$, 332.353 g mol^{-1}

2-{[(*Tert*-butoxy)carbonyl]amino}acetyl 2-{[(*tert*-butoxy)carbonyl]amino}acetate was prepared following a procedure from the literature and used immediately for the synthesis of *tert*-butyl *N*-{[(8-hydroxyquinolin-5-yl)carbamoyl]methyl}carbamate, without characterisation.[5]

Tert-butyl *N*-{[(8-hydroxyquinolin-5-yl)carbamoyl]methyl}carbamate, 6

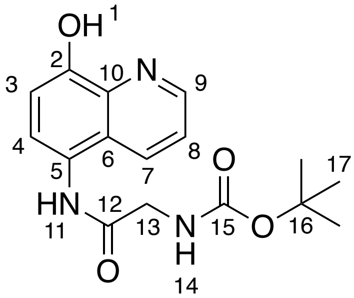

$C_{16}H_{19}N_3O_4$, 317.345 g mol^{-1}

To a solution of 5-amino-8-hydroxyquinoline dihydrochloride (1.00 g, 4.29 mmol) and DIPEA (2.39 mL, 13.7 mmol) in dry DMF, was added 2-{[(*tert*-butoxy)carbonyl]amino}acetyl 2-{[(*tert*-butoxy)carbonyl]amino}acetate (2.16 g, 7.75 mmol) dropwise over 30 min at 0 °C under a nitrogen atmosphere. The reaction mixture was then stirred for 2 d at room temperature. After this time, the mixture was quenched with water (10 mL) and the solvent removed *in vacuo*. The resulting solid was re-dissolved in EtOAc (70 mL) and washed with $NaHCO_3$ (aq) (30 mL, x3) and

then water (30 mL, x3). The organic layer solvent was then removed *in vacuo*, to yield the title compound crude, as a green solid.

Yield: 2.29 g, 7.22 mmol, 93% (crude)

Rf: 0.24 in dichloromethane : MeOH : DIPEA (95:15:1)

HRMS (ESI+):

Calc. for $[C_{16}H_{19}N_3O_4]+H^+$ = 318.1448 Found = 318.1443 (1.6 ppm error, 2.0 mSigma)

Calc. for $[C_{16}H_{19}N_3O_4]+Na^+$ = 340.1268 Found = 340.1267 (0.3 ppm error, 1.8 mSigma)

^{1}H NMR: (400 MHz, $CDCl_3$) δ: 8.73 (d, *J* = 4.5 Hz, 1H-**9**), 8.52 (br s, 1H-**11**), 8.16 (d, *J* = 7.0 Hz, 1H-**7**), 7.61 – 7.50 (m, 1H-**4**), 7.37 (dd, *J* = 9.0, 4.0 Hz, 1H-**8**), 7.07 (d, *J* = 8.0 Hz, 1H-**3**), 5.58 – 5.41 (m, 1H-**14**), 4.03 (d, *J* = 6.0 Hz, 1H-**13**), 1.49 (s, 9H-**17**).

^{13}C NMR: (101 MHz, $CDCl_3$) δ: 169.4-**12**, 157.0-**15**, 151.0-**2**, 148.0-**9**, 138.2-**10**, 131.7-**7**, 124.2-**4**, 124.0-**5**, 123.1-**3**, 122.0-**6**, 109.4, 81.0-**13**, 45.6-**16**, 28.5-**17**.

IR ATIR (cm^{-1}): 3322 m (O-H stretch), 3233 m (N-H stretch), 2978 m (Aromatic/Alkene C-H stretch), 2930 m (Alkane C-H stretch), 2801 w (Alkane C-H stretch), 1715 s (C=O stretch), 1691 s (C=O stretch).

***Tert*-butyl *N*-({[8-(prop-2-en-1-yloxy)quinolin-5-yl]carbamoyl}methyl)carbamate, 7**

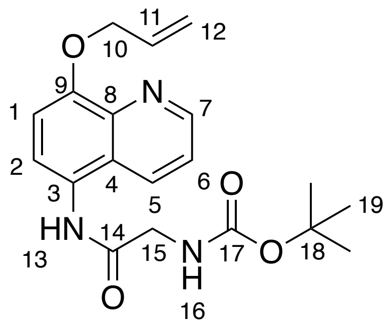

$C_{19}H_{23}O_3N_4$, 357.410 g mol^{-1}

To a stirring solution of crude *tert*-butyl *N*-{[(8-hydroxyquinolin-5-yl)carbamoyl]methyl}-carbamate (28.3 mmol, 8.98 g) in dry DMF (50 mL), was added potassium carbonate (12.4 mmol, 1.71 g) and allyl bromide (12.4 mmol, 1.07 mL) portion-wise, at room temperature. This reaction mixture was then heated to 50 °C and left stirring for 18 h. After this time, reaction solvent was removed *in vacuo* and the residue re-dissolved in dichloromethane (100 mL). This solution was then washed with $NaHCO_3$ (aq) (50 mL, x2) and water (50 mL, x2). The organic layers were combined, and the solvent removed *in vacuo*. The resulting residue was purified *via* column chromatography, eluting with EtOAc : NEt_3 (99:1) and then hot filtration in EtOAc, to yield the title compound as a beige crystalline solid.

Yield: 1.41 g, 3.95 mmol, 14%

Rf: 0.82 in dichloromethane : MeOH (19:1)

HRMS (ESI+):

Calc. for $[C_{19}H_{23}O_3N_4]+H^+$ = 358.1761 Found = 358.1762 (-0.2 ppm error, 0.3 mSigma)

^{1}H NMR: (400 MHz, $CDCl_3$) δ: 8.92 (dd, *J* = 4.0, 2.0 Hz, 1H-**7**), 8.74 (br s, 1H-**13**), 8.21 (d, *J* = 8.5 Hz, 1H-**5**), 7.63 (d, *J* = 8.5 Hz, 1H-**2**), 7.38 (dd, *J* = 8.5, 4.0 Hz, 1H-**6**), 6.98 (dd, *J* = 8.0 Hz, 1H-**1**), 6.15 (ddt, *J* = 16.0, 10.5, 5.5 Hz, 1H-**11**), 5.58 (t, *J* = 6.0 Hz, 1H-**16**), 5.44 (dd, *J* = 17.5, 1.5 Hz, 1H-**12(trans)**), 5.31 (dd, *J* = 10.5, 1.0 Hz, 1H-**12(cis)**), 4.82 (d, *J* = 5.5 Hz, 2H)-**10**, 4.04 (d, *J* = 6.0 Hz, 2H-**15**), 1.47 (s, 9H-**19**).

^{13}C NMR: (101 MHz, $CDCl_3$) δ: 169.4-**14/17**, 157.0-**14/17**, 152.6-**9**, 149.2-**7**, 140.1-**4**, 133.0-**11**, 130.9-**8**, 124.6-**5**, 122.8-**6**, 121.7-**2**, 118.7-**12**, 108.9-**1**, 81.0-**15**, 70.05-**10**, 45.6-**18**, 28.4-**19**.

IR ATIR (cm^{-1}): 3220 m (Amide N-H stretch), 3019 m (Aromatic/Alkene C-H stretch), 2976 m (Aromatic C-H stretch), 1700 s (C=O stretch), 1657 s (C=O stretch), 1618 s (Alkene C=C stretch), 1537 s (Aromatic C=C stretch).

Elemental Analysis:

Calculated for [$C_{19}H_{23}O_3N_4$ • 0.05 H_2O • 0.05 $C_4H_8O_2$]: %C 63.58, %H 6.53, %N 11.58

Measured for [$C_{19}H_{23}O_3N_4$ • 0.05 H_2O • 0.05 $C_4H_8O_2$]: %C 63.53, %H 6.37, %N 11.43

Melting Point: 149 – 151 °C

5-(2-Azaniumylacetamido)-8-(prop-2-en-1-yloxy)quinolin-1-ium ditrifluoroacetate, Glycine-linked 8-allyloxyquinoline

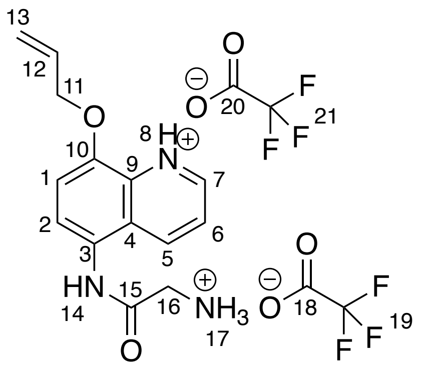

$C_{18}H_{17}F_6N_3O_6$, 485.339 g mol^{-1}

To a solution of *tert*-butyl *N*-({[8-(prop-2-en-1-yloxy)quinolin-5-yl]carbamoyl}-methyl)carbamate (1.129 mmol, 426 mg) in dichloromethane (20 mL), was added TFA (2 mL) at 0 °C. This solution was stirred for 5 h and allowed to warm to room temperature. The reaction solvent was removed *in vacuo* by azeotroping with MeOH (10 mL, x3) and the resulting residue triturated with Et_2O and filtered, to yield the title compound as a beige solid.

Yield: 440 mg, 1.185 mmol, 99%

Rf: 0.45 in dichloromethane : MeOH (8:2) with 1% NH_3 (aq)

HRMS (ESI+):

Calc. for [$C_{14}H_{15}N_3O_2$]+H^+ = 258.1237 Found = 258.1236 (0.3 ppm error, 3.0 mSigma)

Calc. for $[C_{14}H_{15}N_3O_2]+Na^+$ = 280.1056 Found = 280.1055 (0.5 ppm error, 9.4 mSigma)

^{1}H NMR: (400 MHz, DMSO-d_6) δ: 10.42 (s, 1H-**14**), 8.95 (dd, *J* = 4.0, 1.5 Hz, 1H-**7**), 8.52 (d, *J* = 8.5 Hz, 1H-**5**), 8.22 (brs, 3H-**17**), 7.68 (dd, *J* = 8.5, 4.0 Hz, 1H-**6**), 7.60 (d, *J* = 8.5 Hz, 1H-**2**), 7.29 (d, *J* = 8.5 Hz, 1H-**1**), 6.17 (ddt, *J* = 17.0, 10.5, 5.5 Hz, 1H-**12**), 5.52 (dd, *J* = 17.5, 1.5 Hz, 1H-**13(trans)**), 5.33 (dd, *J* = 10.5, 1.5 Hz, 1H-**13(cis)**), 4.81 (d, *J* = 5.5 Hz, 2H-**11**), 3.95 (d, *J* = 5.0 Hz, 2H-**16**).

^{19}F NMR: (376 MHz, MeOD-d_4) δ: -76.8 (s, 6F-**19+21**).

^{13}C NMR: (101 MHz, DMSO-d_6) δ: 166.1-**15**, 158.3 (q, *J* = 34.0 Hz)-**18/20**, 151.6-**10**, 148.7-**7**, 138.5-**9**, 133.5-**12**, 132.9-**5**, 125.1-**3**, 124.6-**4**, 123.3-**2**, 121.8-**6**, 118.0-**13**, 109.9-**1**, 69.3-**11**, 40.8-**16**.

IR ATIR (cm^{-1}): 3219 w (N-H stretch), 3050 m (Aromatic/Alkene C-H stretch), 1684 s (C=O stretch), 1671 s (C=O stretch), 1595 w (Aromatic C=C stretch).

Elemental Analysis:

Calculated for $[C_{14}H_{15}N_3O_2 \cdot 1.9\ C_2HF_3O_2 \cdot 0.1\ C_4H_{10}O]$: %C 45.79, %H 3.60, %N 8.85

Measured for $[C_{14}H_{15}N_3O_2 \cdot 1.9\ C_2HF_3O_2 \cdot 0.1\ C_4H_{10}O]$: %C 45.84, %H 3.61, %N 8.90

Melting Point: 182 – 184 °C

2.5. The Synthesis of Ru-s1

2,3-Bis(acetyloxy)benzoic acid, 8

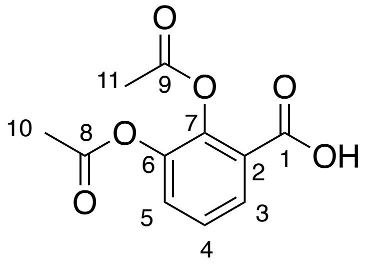

$C_{11}H_{10}O_6$, 238.195 g mol^{-1}

2,3-Bis(acetyloxy)benzoic acid was prepared following a procedure from the literature.[4]

Yield: 7.42 g, 31.2 mmol, 96%

Rf: 0.87 in dichloromethane : MeOH (9:1)

HRMS (ESI+):

Calc. for $[C_{11}H_{10}O_6]+Na^+$ = 261.0370 Found = 261.0365 (1.6 ppm error, 2.4 mSigma)

^{1}H NMR: (400 MHz, $CDCl_3$) δ: 7.99 (dd, *J* = 8.0, 1.5 Hz, 1H-**3**), 7.44 (dd, *J* = 8.0, 1.5 Hz, 1H-**5**), 7.36 (t, *J* = 8 Hz, 1H-**4**), 2.34 (s, 3H-**10**/**11**), 2.23 (s, 3H-**10**/**11**).

^{13}C NMR: (101 MHz, $CDCl_3$) δ: 168.8-**8/9**, 168.6-**1**, 168.4-**8/9**, 143.8-**2**, 143.3-**7**, 129.8-**3**, 128.9-**5**, 126.4-**4**, 124.0-**6**, 20.8-**10**+**11**.

NMR spectra are in agreement with literature reports.[4]

2,3-Bis(acetyloxy)benzoyl 2,3-bis(acetyloxy)benzoate, 9

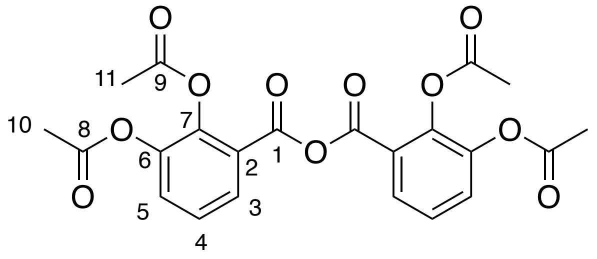

$C_{22}H_{18}O_{11}$, 458.375 g mol^{-1}

2,3-Bis(acetyloxy)benzoyl 2,3-bis(acetyloxy)benzoate was prepared based on a procedure from the literature and used immediately for the synthesis of 2-(acetyloxy)-3-[({[8-(prop-2-en-1-yloxy)quinolin-5-yl]carbamoyl} methyl)carbamoyl]-phenyl acetate, without characterisation.[5]

To a solution of 2,3-bis(acetyloxy)benzoic acid (370 mg, 1.55 mmol) in dry dichloromethane (15 mL), was added dicyclohexylcarbodiimide (160 mg, 0.780 mmol) at room temperature under a nitrogen atmosphere and left stirring for 1 h. After this time, the resulting suspension was filtered, and the filtrate solvent was removed *in vacuo*. The resulting white solid was used immediately for the synthesis of 2-(acetyloxy)-3-[({[8-(prop-2-en-1-yloxy)quinolin-5-yl]carbamoyl} methyl)carbamoyl]phenyl acetate.

2-(Acetyloxy)-3-[({[8-(prop-2-en-1-yloxy)quinolin-5-yl]carbamoyl} methyl)carbamoyl]phenyl acetate, 10

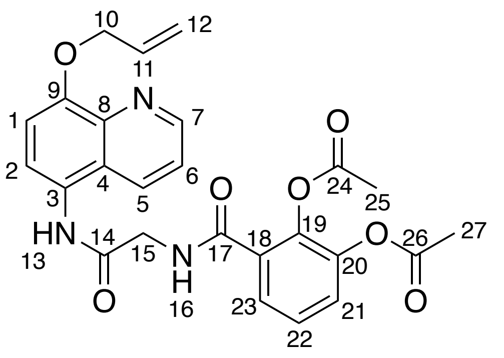

$C_{25}H_{23}N_3O_7$, 477.473 g mol^{-1}

To a stirred solution of 5-(2-azaniumylacetamido)-8-(prop-2-en-1-yloxy)quinolin-1-ium ditrifluoroacetate (100 mg, 0.206 mmol) in dry DMF (20 mL) was added two molar equivalents of crude 2,3-bis(acetyloxy)benzoyl 2,3-bis(acetyloxy)benzoate as a solution in dry dichloromethane (20 mL), dropwise over 30 min, under a nitrogen atmosphere at room temperature. After stirring under these conditions for 3 h, water (10 mL) was added to quench and the solvent removed *in vacuo*. The resulting solid residue was then re-dissolved in EtOAc (30 mL) and washed with water (30 mL, x5). The resulting organic layer solvent was removed *in vacuo* and purified *via* column chromatography, eluting with $CHCl_3$ in MeCN gradient (6:4 - 1:1) and then triturated with Et_2O, to yield the title compound as a beige solid.

Yield: 58 mg, 121 μmol, 45%

Rf: 0.23 in $CHCl_3$: MeCN (6:4)

HRMS (ESI+):

Calc. for $[C_{25}H_{24}O_3N_7]+H^+$ = 478.1609 Found = 478.1616 (-1.6 ppm error, 35.1 mSigma)

^{1}H NMR: (400 MHz, $CDCl_3$) δ: 9.00 (br s, 1H-**13**), 8.89 (dd, *J* = 4.0, 1.5 Hz, 1H-**7**), 8.22 (dd, *J* = 8.5, 1.0 Hz, 1H-**5**), 7.78 (t, *J* = 5.0 Hz, 1H-**16**), 7.62 (dd, J = 7.5, 1.5 Hz, 1H-**21**/**23**), 7.59 (d, J = 8.5 Hz, 1H-**2**), 7.34 – 7.28 (m, 3H-**6**+**21**/**23**), 7.22 (t, *J* = 8.0 Hz,

1H-**22**), 6.98 (d, *J* = 8.5 Hz, 1H-**1**), 6.17 (ddt, *J* = 16.0, 10.5, 5.5 Hz, 1H-**11**), 5.45 (dd, *J* = 17.5, 1.5 Hz, 1H-**12(trans)**), 5.32 (dd, *J* = 10.5, 1.5 Hz, 1H-**12(cis)**), 4.82 (d, *J* = 5.5 Hz, 2H-**10**), 4.44 (d, *J* = 5.0 Hz, 2H-**15**), 2.28 (s, 3H-**25/27**), 2.24 (s, 3H- **25/27**).

^{13}C NMR: (101 MHz, $CDCl_3$) δ: 168.5-**14**, 168.3-**24**+**26**, 165.8-**17**, 152.9-**9**, 149.4-**7**, 143.0-**19**, 140.5-**3**, 140.3-**20**, 133.0-**11**, 131.1-**5**, 128.8-**8**, 127.2-**21/23**, 126.7-**21/23**, 126.7-**22**, 124.8-**4**, 124.6-**18**, 123.1-**2**, 121.9-**6**, 118.7-**12**, 108.7-**1**, 70.1-**15**, 44.6-**10**, 20.8-**25/27**, 20.7-**25/27**.

IR ATIR (cm^{-1}): 3270 w (Amide N-H stretch), 2924 w (Aromatic/Alkene C-H stretch), 2854 w (Alkane C-H stretch), 1766 s (C=O stretch), 1655 m (Alkene C=C stretch), 1525 (Aromatic C=C stretch).

Elemental Analysis:

Calculated for [$C_{25}H_{23}N_3O_7$ • 0.45 CH_2Cl_2 • 0.55 $C_4H_{10}O$]: %C 59.68, %H 5.33, %N 7.55

Measured for [$C_{25}H_{23}N_3O_7$ • 0.45 CH_2Cl_2 • 0.55 $C_4H_{10}O$]: %C 59.95, %H 4.89, %N 7.13

Ru-s1

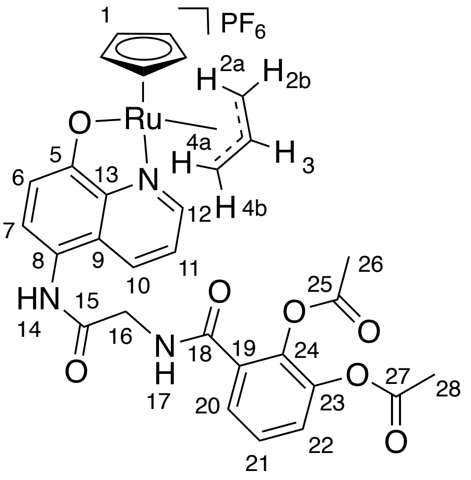

$C_{30}H_{28}F_6N_3O_7PRu$, 788.602 g mol^{-1}

To a solution of tris(acetonitrile)cyclopentadienylruthenium(II) hexafluorophosphate (26 mg, 59.9 μmol) in dry, degassed dichloromethane (20 mL), was added

2(acetyloxy)-3-[({[8-(prop-2-en-1-yloxy)quinolin-5-yl]carbamoyl}methyl)carbamoyl] phenyl acetate (29 mg, 59.9 μmol) in dry, degassed dichloromethane (4 mL) under a dry, nitrogen atmosphere at room temperature to form, at first a dark red solution which turned to a light red solution over 2 min. After stirring under these conditions for 1 h, the reaction suspension was allowed to settle under gravity and the clear, orange supernatant was removed. The remaining solid was washed with dry dichloromethane (1 mL, x3) to yield the title compound as dark brown solid.

Yield: 27 mg, 41.4 μmol, 69%

HRMS (LIFDI):

Calc. for $[C_{30}H_{28}N_3O_7Ru]^+$ = 644.09653 Found = 644.09966 (4.87 ppm error, 18.5 DBE)

^{1}H NMR: (400 MHz, acetone-d_6) δ: 9.17 (s, 1H-**14**), 8.96 (d, *J* = 5.0 Hz, 1H-**12**), 8.50 (d, *J* = 8.5 Hz, 1H-**10**), 8.02 (t, *J* = 4.5 Hz, 1H-**17**), 7.75 (dd, *J* = 5.5, 4.0 Hz, 1H-**20**), 7.60 (dd, *J* = 8.5, 5.0 Hz, 1H-**11**), 7.43 – 7.39 (m, 2H-**21**+**22**), 7.38 (d, *J* = 8.5 Hz, 1H-**6**), 6.90 (d, *J* = 8.5 Hz, 1H-**7**), 6.30 (s, 5H-**1**), 4.75 – 4.62 (m, 2H-**3+2a/4a^1**), 4.40 (d, *J* = 10.0 Hz, 1H-**2a/4a^2**), 4.36 (dd, *J* = 6.0, 3.0 Hz, 1H-**2b/4b^2**), 4.30 (d, *J* = 5.5 Hz, 2H-**16**), 4.18 (dd, *J* = 6.0, 3.0 Hz, 1H-**2b/4b^1**), 2.30 (s, 3H-**26/28**), 2.28 (s, 3H-**26/28**).

^{13}C NMR: (126 MHz, acetone-d_6) δ: 169.6-**15**, 169.1-**5**, 168.8-**25/27**, 168.8-**25/27**, 165.8-**18**, 156.3-**12**, 146.3-**9**, 144.4-**19**, 141.7-**23**, 137.3-**10**, 130.8-**24**, 128.4-**6**, 127.6-**20**, 127.5-**13**, 127.1-**21/22**, 127.1-**21/22**, 124.2-**11**, 119.9-**8**, 115.1-**7**, 99.5-**3**, 97.0-**1**, 70.0-**2/4**, 63.6-**2/4**, 44.4-**16**, 20.7-**26/28**, 20.5-**26/28**.

^{19}F NMR: (565 MHz, acetone-d_6) δ: -72.5 (d, *J* = 707.5 Hz, 6F).

^{19}P NMR: (243 MHz, acetone-d_6) δ: -144.3 (sp, *J* = 707.0 Hz, 1P).

IR ATIR (cm^{-1}): 3393 w br (Amide N-H stretch), 3117 w br (Aromatic/Alkene C-H stretch), 2964 m (Aromatic/Alkene C-H stretch), 2912 w (Aromatic/Alkene C-H stretch), 2801 w (Alkane C-H stretch), 1769 s (C=O stretch), 1696 s (C=O stretch), 1660 s (Alkene C=C stretch), w 1573 (Aromatic C=C stretch).

Elemental Analysis:

Calculated for [$C_{30}H_{28}F_6N_3O_7PRu$ • 2 CH_2Cl_2]: %C 40.10, %H 3.37, %N 4.38

Measured for [$C_{30}H_{28}F_6N_3O_7PRu$ • 2 CH_2Cl_2]: %C 39.71, %H 3.37, %N 3.93

UV-vis Spectroscopy:

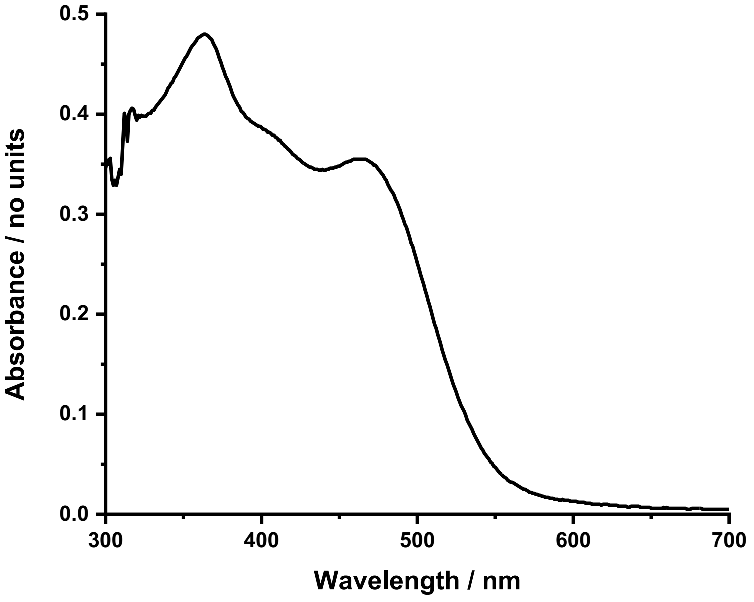

Figure S 4 UV-vis spectrum, 200 μM in DMSO.

2.6. The Synthesis of Ru-s2

2-[(2,3-Dihydroxyphenyl)formamido]-*N*-[8-(prop-2-en-1-yloxy) quinolin-5-yl]acetamide, 11

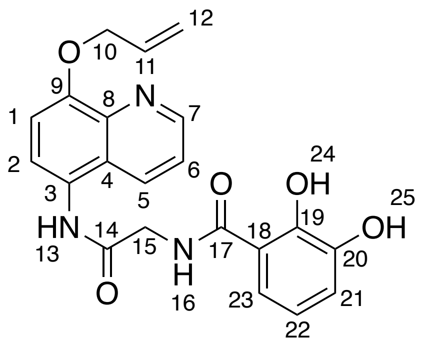

$C_{21}H_{19}N_3O_5$, 393.40 g mol^{-1}

To a stirred solution of 2-(acetyloxy)-3-[({[8-(prop-2-en-1-yloxy)quinolin-5-yl]carbamoyl}methyl)carbamoyl]phenyl acetate (111 mg, 0.232 mmol) in dry dichloromethane (3 mL) at 0 °C was added piperidine (138 μL, 1.40 mmol), and the resulting mixture was left stirring for 45 min. After this time, the solvent was removed *in vacuo* and the resulting residue was triturated in Et_2O (10 mL), and then washed with dichloromethane (x3, 5 mL) to yield the title compound as a white solid.

Yield: 87 mg, 221 μmol, 95%

Rf: 0.56 in dichloromethane : MeOH (4:1)

HRMS (ESI-):

Calc. for $[C_{21}H_{19}N_3O_5]$-H^+ = 392.1252 Found = 392.1257 (-1.4 ppm error, 1.9 mSigma)

^{1}H NMR: (600 MHz, DMSO-*d6*) δ: 16.17 – 15.95 (br s, 1H-**24**), 10.02 (s, 1H-**13**), 9.37 (s, 1H-**25**), 8.88 (dd, *J* = 4.0, 1.5 Hz, 1H-**7**), 8.39 (dd, *J* = 8.5, 1.5 Hz, 1H-**5**), 7.59 (dd, *J* = 8.5, 4.0 Hz, 1H-**6**), 7.51 (d, *J* = 8.5 Hz, 1H-**1**), 7.34 (d, *J* = 8.0 Hz, 1H-**23**), 7.20 (d, *J* = 8.5 Hz, 1H-**2**), 6.90 (d, *J* = 7.5 Hz, 1H-**21**), 6.66 (br s, 1H-**22**), 6.16 (ddd, *J* = 22.5, 10.5, 5.5 Hz, 1H-**11**), 5.51 (dd, *J* = 17.5, 2.0 Hz 1H-**12(trans)**), 5.35 – 5.23 (dd, *J* = 10.5, 1.5 Hz 1H-**12(cis)** 2H), 4.78 (d, *J* = 5.0 Hz, 2H-**10**), 4.22 (d, *J* = 5.5 Hz, 2H-**15**).

^{13}C NMR: (151 MHz, DMSO-*d6*) δ: 170.4-**17**, 168.9-**14**, 152.6-**9**, 149.5-**7**, 146.7-**18**, 140.3-**3**, 134.2-**11**, 132.2-**6**, 126.3-**4**, 125.3-**8**, 123.7-**2**, 121.9-**5**, 119.1-**19**, 118.3-**21/23/12**, 118.2-**21/23/12+21/23/12**, 115.8-**20**, 109.8-**1**, 69.6-**10**, 43.33-**15**.

In some cases, peaks appear to overlap and therefore are hidden by others. This is believed to be the case at:

- 118.2 ppm = two of **21**, **23**, and **12** - which are undistinguishable
- 118.3 ppm = one of **21**, **23**, and **12** - which are undistinguishable

IR ATIR (cm^{-1}): 3301 m (O-H stretch), 3072 w (Aromatic/Alkene C-H stretch), 2936 w (Alkane C-H stretch), 2852 w (Alkane C-H stretch), 2714 w (Alkane C-H stretch), 1684 m (C=O stretch), 1585 s (Aromatic C=C stretch).

Elemental Analysis:

Calculated for [$C_{21}H_{19}N_3O_5$ • 0.4 CH_2Cl_2 • 0.05 $C_4H_{10}O$]: %C 60.18, %H 4.75, %N 9.44
Measured for [$C_{21}H_{19}N_3O_5$ • 0.4 CH_2Cl_2 • 0.05 $C_4H_{10}O$]: %C 60.48, %H 4.50, %N 9.44

Melting Point: 209 – 211 °C

Ru-s2

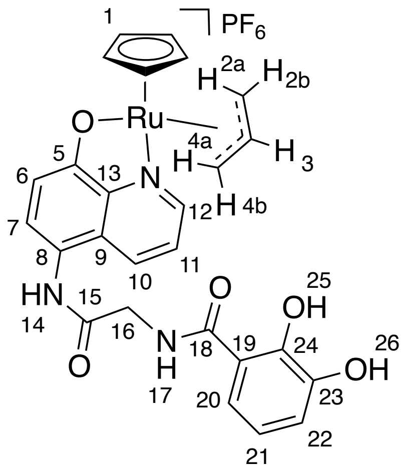

$C_{26}H_{24}F_6N_3O_5PRu$, 704.528 g mol^{-1}

To a stirred solution of tris(acetonitrile)cyclopentadienylruthenium(II) hexafluorophosphate (12.47 mg, 28.7 µmol) in dry, degassed DMF (0.5 mL) was added 2-[(2,3-dihydroxyphenyl)formamido]-*N*-[8-(prop-2-en-1-yloxy) quinolin-5-yl]acetamide (10.49 mg, 28.7 µmol) as a solution in dry, degassed DMF (3 mL) under a dry, nitrogen atmosphere at room temperature to form, at first a dark red solution which turned to a light red solution over 2 min. After stirring for 30 min under these conditions, the ruthenium-complex was precipitated using dry Et_2O (45 mL), where the resulting supernatant was decanted to leave the solid. This solid was washed with dry Et_2O (20 mL, x4) and then dry dichloromethane (20 mL, x2), to yield the title compound as a red/orange solid.

Yield: 17 mg, 24.1 µmol, 84%

HRMS (ESI+):

Calc. for $[C_{23}H_{20}N_3O_5Ru]^+$ = 520.0441 Found = 520.0412 (6.8 ppm error, 46.7 mSigma)

Calc. for $[C_{23}H_{19}N_3O_5Ru]+Na^+$ = 542.0260 Found = 542.0236 (5.7 ppm error, 39.2 mSigma)

Calc. for $[C_{23}H_{18}N_3O_5Ru]+2Na^+$ = 564.0080 Found = 564.0079 (1.2 ppm error, 160.2 mSigma)

Calc. for $[C_{23}H_{19}N_3O_7Ru]+Na^+$ = 574.0159 Found = 574.0121 (7.6 ppm error, 249.0 mSigma)

Calc. for $[C_{23}H_{18}N_3O_7Ru]+2Na^+$ = 595.9966 Found = 595.9978 (3.1 ppm error, 167.2 mSigma)

^{1}H NMR: (600 MHz, acetone-*d*6) δ: 12.64 (s, 1H-**25/26**), 9.34 (s, 1H-**14**), 8.98 (d, *J* = 5.0 Hz, 1H-**12**), 8.64 (br s, 1H), 8.50 (dd, *J* = 9.0, 1.0 Hz, 1H-**10**), 7.84 (br s, 1H), 7.62 (dd, *J* = 8.5, 5.0 Hz, 1H-**11**), 7.34 (d, *J* = 8.5, 1H-**6**), 7.32 (dd, *J* = 8.0, 1.0 Hz, 1H-**20**), 7.01 (d, *J* = 8.0 Hz, 1H-**22**), 6.89 (d, *J* = 8.5 Hz, 1H-**7**), 6.77 (t, *J* = 8.0 Hz, 1H-**21**), 6.32 (s, 5H-**1**), 4.79 – 4.57 (m, 2H-**3+2a/4a**), 4.42 (d, *J* = 10.0 Hz, 1H-**2a/4a**), 4.38 (dd, *J* = 5.5, 2.5 Hz, 1H-**2b/4b**), 4.31 (d, *J* = 5.5 Hz, 2H-**16**), 4.19 (dd, *J* = 6.0, 3.0 Hz, 1H-**2b/4b**).

^{19}F NMR: (471 MHz, acetone-*d*6) δ: -72.5 (d, *J* = 707.5 Hz, 6F).

^{19}P NMR: (202 MHz, acetone-*d*6) δ: -144.2 (sp, *J* = 707.5 Hz, 1P).

^{13}C NMR: (126 MHz, acetone-*d*6) δ: 172.0-**18**, 169.3-**15/5**, 169.1-**15/5**, 156.3-**12**, 150.8-**8**, 147.3-**23**, 146.3-**9**, 137.4-**10**, 128.5-**24/6**, 128.4-**24/6**, 127.6-**13**, 124.2-**11**, 120.0-**19**, 119.7-**22**, 119.3-**21**, 118.0-**20**, 115.1-**7**, 99.5-**16**, 97.1-**1,** 70.0-**2/4**, 63.6-**2/4**, 44.1-**16**.

IR ATIR (cm^{-1}): 3388 w br (Amide N-H stretch), 3117 w br (Aromatic/Alkene C-H stretch), 3107 m (Aromatic C-H stretch), 1644 m (C=O stretch), 1572 m (C=O stretch), 1537 s (Aromatic C=C stretch).

Elemental Analysis:

Calculated for [$C_{26}H_{24}F_6N_3O_5PRu$ • 0.15 $C_4H_{10}O$ • 0.1 C_3H_7NO • 0.75 CH_2Cl_2]: %C 42.22, %H 3.55, %N 5.52
Measured for [$C_{26}H_{24}F_6N_3O_5PRu$ • 0.15 $C_4H_{10}O$ • 0.1 C_3H_7NO • 0.75 CH_2Cl_2]: %C 42.12, %H 3.63, %N 5.53

UV-vis Spectroscopy:

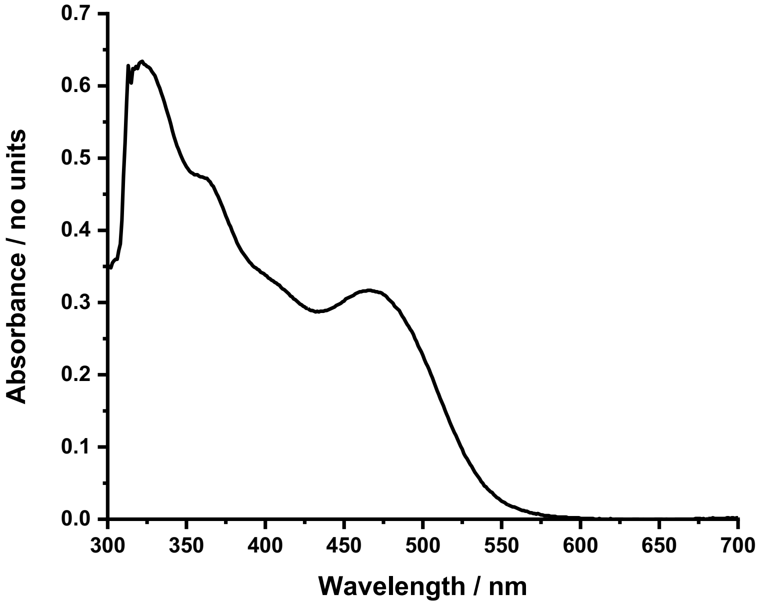

Figure S 5 UV-vis spectrum, 200 μM in DMSO.

2.7. The Synthesis of Ru-s3

2-(Hydroxymethyl)-5-[(4-methoxyphenyl)methoxy]-4H-pyran-4-one, 12

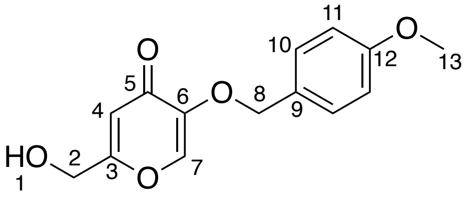

$C_{14}H_{14}O_5$, 262.26 g mol^{-1}

2-(Hydroxymethyl)-5-[(4-methoxyphenyl)methoxy]-4H-pyran-4-one was prepared based on a procedure from the literature.[7]

A suspension of kojic acid (2.29 g, 16.0 mmol), potassium carbonate (2.66 g, 19.3 mmol), 4-methoxybenzyl chloride (2.39 mL, 17.7 mmol) in dry DMF was heated to 80 °C and left stirring for 18 h. After this time, the reaction solvent was removed *in vacuo*, and the resulting beige residue was re-suspended and sonicated in water (100 mL) for 2 h. The solid produced from this sonication was isolated by filtration and washed with more water (100 mL) and finally EtOAc (50 mL) to give the title compound as a beige solid.

Yield: 3.14 g, 12.0 mmol, 74%

Rf: 0.56 in dichloromethane : MeOH (8:2)

HRMS (ESI+):

Calc. for $[C_{14}H_{14}O_5]+Na^+$ = 285.0733 Found = 285.0731 (0.9 ppm error, 3.1 mSigma)

^{1}H NMR: (400 MHz, DMSO-*d*$_6$) δ 8.15 (s, 1H-**7**), 7.34 (d, *J* = 8.5 Hz, 2H-**10**), 6.95 (d, *J* = 8.5 Hz, 2H-**11**), 6.31 (s, 1H-**4**), 5.69 (br s, 1H-**1**), 4.85 (s, 2H-**8**), 4.29 (s, 2H-**2**), 3.76 (s, 3H-**13**).

^{13}C NMR: (126 MHz, DMSO-d_6) δ: 173.3-**5**, 168.0-**3**, 159.3-**12**, 146.6-**6**, 141.1-**7**, 130.1-**10**, 128.1-**9**, 113.8-**11**, 111.2-**4**, 70.3-**8**, 59.3-**2**, 55.1-**13**.

NMR spectra are in agreement with literature reports.[7]

Elemental Analysis:

Calculated for [$C_{14}H_{14}O_5$ • 0.25 H_2O]: %C 63.03, %H 5.48
Measured for [$C_{14}H_{14}O_5$ • 0.25 H_2O]: %C 63.00, %H 5.24

{5-[(4-Methoxyphenyl)methoxy]-4-oxo-4*H*-pyran-2-yl}methyl-*N*-({[8-(prop-2-en-1-yloxy)quinolin-5-yl]carbamoyl}methyl)carba-mate, 13

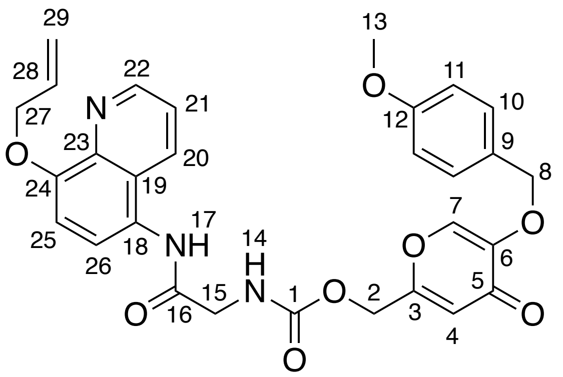

$C_{29}H_{27}N_3O_8$, 545.548 g mol^{-1}

{5-[(4-Methoxyphenyl)methoxy]-4-oxo-4H-pyran-2-yl}methyl-*N*-({[8-(prop-2-en-1-yloxy)quinolin-5-yl]carbamoyl}methyl)carbamate was prepared based on a procedure from the literature.[8]

To a stirred solution of 2-(hydroxymethyl)-5-[(4-methoxyphenyl)methoxy]-4H-pyran-4-one (60 mg, 0.229 mmol) in dry DMF (3 mL) was added carbonyldiimidazole (133 mg, 0.817 mmol) and left stirring for 22 h, at room temperature. After this time, a solution of DIPEA (57 µL, 0.326 mmol) and 5-(2-azaniumylacetamido)-8-(prop-2-en-1-yloxy)quinolin-1-ium ditrifluoroacetate (61 mg, 0.163 mmol) in dry DMF (2 mL) was added dropwise and the mixture was left stirring for an additional 18 h, at room temperature. The reaction solvent was then removed *in vacuo*, and the resulting residue re-dissolved in dichloromethane (40 mL) and washed with sat. NH_4Cl(aq) (20

mL, x3) and then sat. $NaHCO_3$(aq) (20 mL, x3). The organic layer solvent was then removed *in vacuo*, and purified *via* column chromatography, eluting with EtOAc : MeOH (23:2) to yield the title compound as a beige solid.

Yield: 40 mg, 72.8 μmol, 45%

Rf: 0.46 in EtOAc : MeOH (9:1)

HRMS (ESI+):

Calc. for $[C_{29}H_{27}N_3O_8]+H^+$ = 546.1871 Found = 546.1858 (2.3 ppm error, 29.2 mSigma)

Calc. for $[C_{29}H_{27}N_3O_8]+Na^+$ = 568.1690 Found = 568.1680 (1.8 ppm error, 5.8 mSigma)

^{1}H NMR: (400 MHz, DMSO-*d6*) δ: 9.92 (s, 1H-**17**), 8.88 (dd, *J* = 4.5, 1.5 Hz, 1H-**22**), 8.33 (dd, *J* = 8.5, 1.5 Hz, 1H-**20**), 8.22 (s, 1H-**7**), 7.89 (t, *J* = 6.0 Hz, 1H-**14**), 7.58 (dd, *J* = 8.5, 4.0 Hz, 1H-**21**), 7.50 (d, *J* = 8.5 Hz, 1H-**25/26**), 7.34 (d, *J* = 8.5 Hz, 2H-**10**), 7.20 (d, *J* = 8.5 Hz, 1H-**25/26**), 6.95 (d, *J* = 8.5 Hz, 2H-**11**), 6.44 (s, 1H-**4**), 6.17 (ddt, *J* = 17.4, 10.5, 5.5 Hz, 1H-**28**), 5.51 (dd, *J* = 17.0, 2.0 Hz, 1H-**29(trans)**), 5.31 (dd, *J* = 10.5, 1.5 Hz, 1H-**29(cis)**), 4.94 (s, 2H-**2**), 4.85 (s, 2H-**8**), 4.77 (dt, *J* = 5.5, 1.5 Hz, 2H-**27**), 3.96 (d, *J* = 6.0 Hz, 2H-**15**), 3.75 (s, 3H-**13**).

^{13}C NMR: (126 MHz, DMSO-*d6*) δ: 173.0-**5**, 168.8-**1/16**, 162.6-**1/16**, 159.3-**12**, 155.8-**3**, 152.1-**24**, 149.0-**22**, 146.8-**6**, 141.4-**7**, 139.8-**18**, 133.7-**28**, 131.2-**20**, 130.1-**10**, 127.9-**9**, 125.7-**19/23**, 124.8-**19/23**, 123.2-**26**, 121.5-**21**, 117.8-**29**, 113.8-**11**, 113.2-**4**, 109.3-**25**, 70.3-**2**, 69.1-**27**, 61.2-**8**, 55.1-**13**, 43.9-**15**.

IR ATIR (cm^{-1}): 3202 m br (N-H stretch), 3035 w br (Aromatic/Alkene C-H stretch), 2951 w (Aromatic/Alkene C-H stretch), 1731 s (C=O stretch), 1659 s (C=O stretch), 1550 s (Alkene C=C stretch), 1504 m (Aromatic C=C stretch).

Elemental Analysis:

Calculated for $[C_{29}H_{27}N_3O_8 \cdot 0.5\ H_2O]$: %C 62.81, %H 5.09, %N 7.58
Measured for $[C_{29}H_{27}N_3O_8 \cdot 0.5\ H_2O]$: %C 62.54, %H 4.82 %N 7.63

Melting Point: 180-182 °C

(5-Hydroxy-4-oxo-4H-pyran-2-yl)methyl-*N*-({[8-(prop-2-en-1-yloxy) quinolin-5-yl]carbamoyl}methyl)carbamate, 14

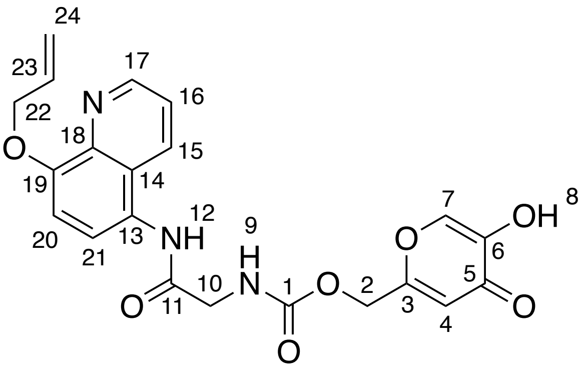

$C_{21}H_{19}N_3O_7$, 425.397 g mol^{-1}

To a stirred solution of {5-[(4-methoxyphenyl)methoxy]-4-oxo-4*H*-pyran-2-yl}methyl-*N*-({[8-(prop-2-en-1-yloxy)quinolin-5-yl]carbamoyl}methyl)carbamate (25.7 mg, 0.0471 mmol) in dichloromethane (2.5 mL) was added trifluoroacetic acid (0.25 mL, 32.7 mmol) dropwise at room temperature, and the resulting solution was left to stir under these conditions for 30 min. After this time, the solvent was removed *in vacuo* with co-evaporation with MeOH (x3, 5 mL), free-based using an SCX cartridge, loading in minimum dichloromethane : MeOH (1:1), washing with MeOH (x3, 5 mL) and eluted with 7N NH_3 in MeOH (5 mL). The resulting solution was then dried *in vacuo*, re-dissolved in minimum dichloromethane : MeOH (1:1) and precipitated with Et_2O to yield the title compound as a beige solid.

Yield: 16.2 mg, 38.1 μmol, 81%

Rf: 0.17 in EtOAc : MeOH (23:1)

HRMS (ESI+):

Calc. for $[C_{21}H_{19}N_3O_7]+H^+$ = 426.1296 Found = 426.1290 (1.4 ppm error, 5.5 mSigma)

Calc. for $[C_{21}H_{19}N_3O_7]+Na^+$ = 448.1115 Found = 448.1118 (-0.5 ppm error, 17.9 mSigma)

^{1}H NMR: (400 MHz, MeOD-d_4 : $CDCl_3$ (5:3)) δ: 9.02 (d, J = 3.5 Hz, 1H-**17**), 8.51 (d, J = 8.5 Hz, 1H-**15**), 8.06 (s, 1H-**7**), 7.71 (dd, J = 14.5, 6.0 Hz, 2H-**16+20/21**), 7.29 (d, J = 8.5 Hz, 1H-**20/21**), 6.70 (s, 1H-**4**), 6.37 (ddt, J = 16.0, 10.5, 5.5 Hz, 1H-**23**), 5.65 (d, J = 17.0 Hz, 1H-**24(trans)**), 5.51 (d, J = 10.5 Hz, 1H-**24(cis)**), 5.17 (s, 2H-**2**), 5.02 (d, J = 5.0 Hz, 2H-**22**), 4.27 (s, 2H-**10**).

^{13}C NMR: (126 MHz, MeOD-d_4 : $CDCl_3$ (5:3)) δ: 175.6-**5**, 170.9-**11+1**, 164.1-**3**, 157.2, 153.3-**19**, 149.3-**17**, 147.0-**6**, 140.4-**7**, 140.1-**13**, 133.5-**15**, 133.2-**28**, 126.4-**14/18**, 125.5-**14/18**, 125.1-**20/21**, 122.5-**16**, 118.5-**24**, 112.1-**4**, 109.6-**20/21**, 70.5-**22**, 62.6-**2**, 44.9-**10**.

IR ATIR (cm^{-1}): 3248 m br (O-H stretch), 3077 w (Aromatic/Alkene C-H stretch), 1745 s (C=O stretch), 1649 s (C=O stretch), 1609 s (Alkene C=C stretch), 1580 m (Aromatic C=C stretch).

Elemental Analysis:

Calculated for [$C_{21}H_{19}N_3O_7$]: %C 62.81, %H 5.09, %N 7.58
Measured for [$C_{21}H_{19}N_3O_7$]: %C 62.54, %H 4.82 %N 7.63

Melting Point: 174-176 °C

Ru-s3

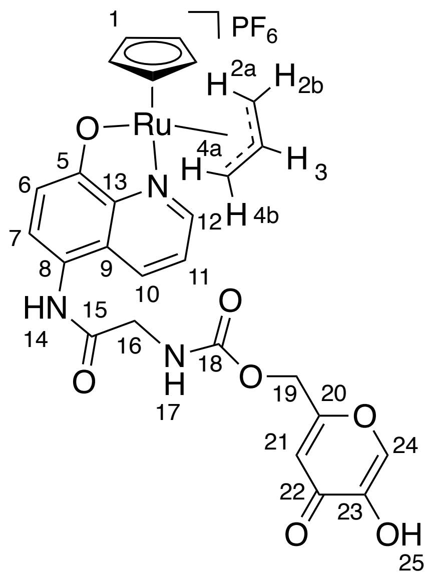

$C_{26}H_{24}F_6N_3O_7PRu$, 736.526 g mol^{-1}

To a stirred solution of tris(acetonitrile)cyclopentadienylruthenium(II) hexafluorophosphate (7.68 mg, 17.7 μmol) in dry, degassed DMF (0.5 mL) was added (5-hydroxy-4-oxo-4H-pyran-2-yl)methyl-*N*-({[8-(prop-2-en-1-yloxy)quinolin-5-yl]carbamoyl}methyl)carbamate (7.52 mg, 17.7 μmol) as a solution in dry, degassed DMF (3 mL) under a dry, nitrogen atmosphere at room temperature to form, at first a dark red solution which turned to a light red solution over 2 min. After stirring for 30 min under these conditions, the reaction mixture was precipitated using dry Et_2O (45 mL), centrifuged (10 min, at 4.4 rps) where the resulting supernatant was decanted to leave the solid. This solid was washed with dry Et_2O (20 mL, x4) and then dry dichloromethane (20 mL, x2), to yield the title compound as a dark orange/brown solid.

Yield: 10.0 mg, 13.6 μmol, 77%

HRMS (ESI+):

Calc. for $[C_{23}H_{19}N_3NaO_7Ru]^+$ = 574.0159 Found = 574.0172 (-1.1 ppm error, 23.7 mSigma)

Calc. for $[C_{26}H_{24}N_3O_7Ru]^+$ = 592.0652 Found = 592.0652 (-0.2 ppm error, 68.3 mSigma)

Calc. for $[C_{23}H_{19}N_3NaO_9Ru]^+$ = 606.0057 Found = 606.0011 (8.7 ppm error, 153.4 mSigma)

^{1}H NMR: (500 MHz, DMSO-*d6*) δ: 9.79 (s, 1H-**14**), 9.21 (s, 1H-**25**), 8.89 (d, *J* = 4.5 Hz, 1H-**12**), 8.35 (d, *J* = 8.5 Hz, 1H-**10**), 8.08 (s, 1H-**24**), 7.84 (t, *J* = 6.0 Hz, 1H-**17**), 7.63 (dd, *J* = 8.5, 5.0 Hz, 1H-**11**), 7.27 (d, *J* = 8.5 Hz, 1H-**7**), 6.87 (d, *J* = 8.5 Hz, 1H-**6**), 6.43 (s, 1H-**21**), 6.25 (s, 5H-**1**), 4.92 (s, 2H-**19**), 4.54 – 4.42 (m, 2H-**3+2a/4a**), 4.25 (d, *J* = 9.5 Hz, 1H-**2a/4a**), 4.14 – 4.09 (m, 1H-**2b/4b**), 4.04 – 3.99 (m, 1H-**2b/4b**), 3.90 (d, *J* = 6.0 Hz, 2H-**16**).

^{13}C NMR: (126 MHz, DMSO-*d6*) δ: 173.6-**22**, 168.9-**15**, 167.2-**5**, 162.5-**20**, 155.8-**18**, 155.7-**12**, 146.0-**23**, 144.7-**13**, 139.7-**24**, 135.9-**10**, 126.7-**7**, 125.9-**9**, 123.3-**11**, 118.9-**8**, 113.8-**6**, 111.9-**27**, 98.0-**3**, 95.8-**1**, 68.2-**2/4**, 63.0-**2/4**, 61.4-**19**, 43.8-**16**.

^{19}F NMR: (471 MHz, DMSO-*d6*) δ: -70.2 (d, *J* = 711.0 Hz, 6F).

^{19}P NMR: (202 MHz, DMSO-*d6*) δ: -144.2 (sp, *J* = 711.0 Hz, 1P).

IR ATIR (cm^{-1}): 3266 w br (O-H stretch), 3110 w (Aromatic/Alkene C-H stretch), 1721 m (C=O stretch), 1649 m (C=O stretch), 1572 m (Alkene C=C stretch), 1500 m (Aromatic C=C stretch).

Elemental Analysis:

Calculated for $[C_{26}H_{24}F_6N_3O_7PRu \cdot 1.0\ CH_2Cl_2 \cdot 0.25\ C_4H_{10}O]$: %C 40.04, %H 3.42, %N 5.00

Measured for $[C_{26}H_{24}F_6N_3O_7PRu \cdot 1.0\ CH_2Cl_2 \cdot 0.25\ C_4H_{10}O]$: %C 40.45, %H 2.98 %N 4.56

UV-vis Spectroscopy:

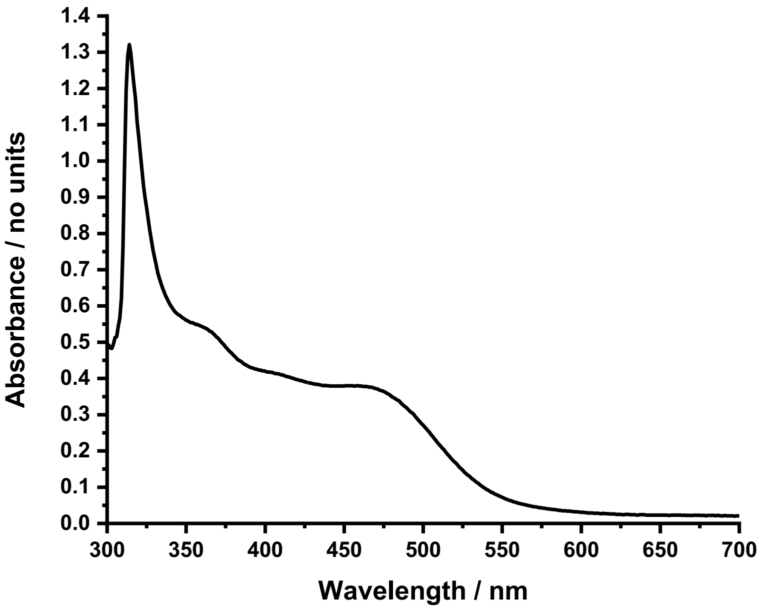

Figure S 6 UV-vis spectrum, 200 μM in DMSO.

2.8. The Synthesis of Ru-s4

2,3-Bis[(4-methoxyphenyl)methoxy]benzoic acid, 15

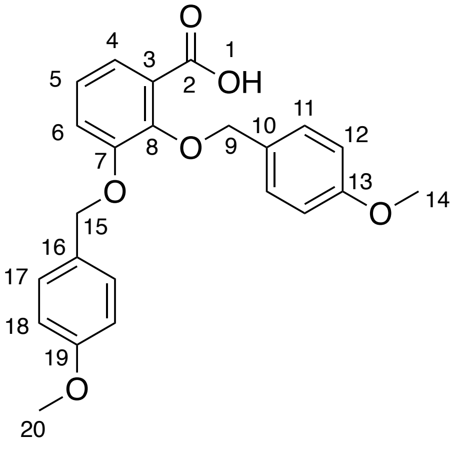

$C_{23}H_{22}O_6$, 394.42 g mol^{-1}

2,3-Bis[(4-methoxyphenyl)methoxy]benzoic acid was prepared following a procedure from the literature.[9]

Yield: 3.60 g, 9.13 mmol, 99%

Rf: 0.20 in EtOAc : PET (1:1)

HRMS (ESI+):

Calc. for $[C_{23}H_{22}O_6]+H^+$ = 393.1344 Found = 393.1237 (4.3 ppm error, 22.9 mSigma)

^{1}H NMR: (400 MHz, $CDCl_3$) δ: 7.72 (dd, *J* = 8.0, 1.5 Hz, 1H-**4**), 7.41 (d, *J* = 8.5 Hz, 1H-**12**), 7.27 – 7.21 (m, 3H-**6+18**), 7.17 (t, *J* = 8.0 Hz, 1H-**5**), 6.96 (d, *J* = 8.5 Hz, 1H-**11**), 6.83 (d, *J* = 8.5 Hz, 1H-**17**), 5.19 (s, 2H-**15**), 5.11 (s, 2H-**9**), 3.84 (s, 3H-**14/20**), 3.79 (s, 3H-**14/20**).

^{13}C NMR: (126 MHz, $CDCl_3$) δ: 165.4-**2**, 160.5-**19**, 160.0-**13**, 151.5-**8**, 147.2-**7**, 131.3-**18**, 129.8-**12**, 128.0-**10**, 126.9-**16**, 125.1-**5**, 124.5-**4**, 123.0-**3**, 119.1-**6**, 114.3-**11+17**, 77.0-**15**, 71.45-**9**, 55.5-**14/20**, 55.4-**14/20**.

Methyl (2*S*)-2,6-bis({2,3-bis[(4-methoxyphenyl)methoxy]phenyl} formamido)hexanoate, 16

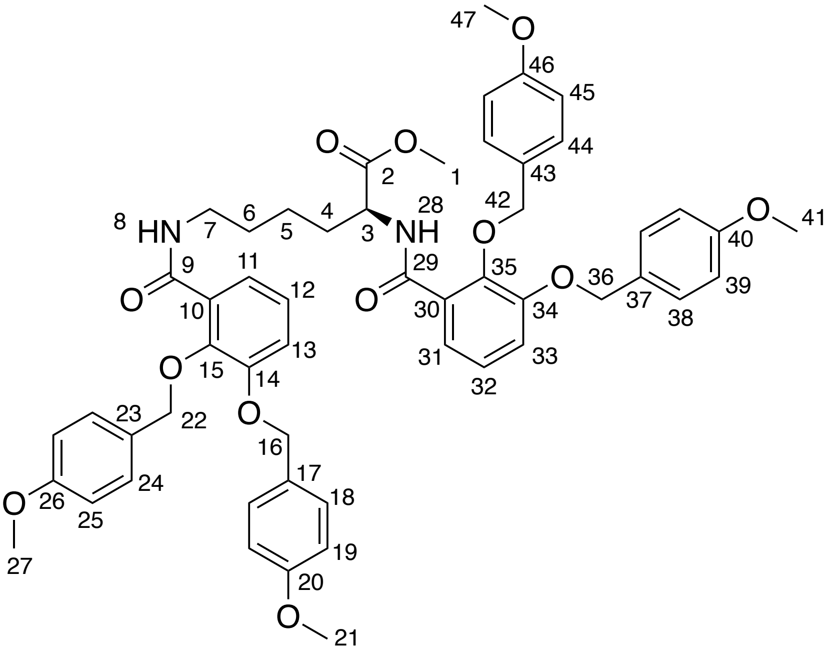

$C_{53}H_{56}N_2O_{12}$, 913.03 g mol^{-1}

Methyl (2*S*)-2,6-bis({2,3-bis[(4-methoxyphenyl)methoxy]phenyl}formamido) hexan-oate was prepared following a procedure from the literature.[9]

Yield: 1.611 g, 1.76 mmol, 72%

Rf: 0.22 in EtOAc : PET (1:1)

HRMS (ESI+):

Calc. for $[C_{53}H_{56}N_2O_{12}]+H^+$ = 913.3906 Found = 913.3919 (-1.4 ppm error, 4.6 mSigma)

Calc. for $[C_{53}H_{56}N_2O_{12}]+Na^+$ = 935.3725 Found = 935.3729 (-0.4 ppm error, 3.0 mSigma)

^{1}H NMR: (400 MHz, $CDCl_3$) δ: 8.55 (d, *J* = 7.5 Hz, 1H-**28**), 7.95 (t, *J* = 5.5 Hz, 1H-**8**), 7.73 – 7.68 (m, 2H-**11+31**), 7.39 (d, *J* = 7.5 Hz, 4H-**Ar**), 7.28 (d, *J* = 8.5 Hz, 2H-**Ar**), 7.20 (d, *J* = 8.5 Hz, 2H-**Ar**), 7.15 – 7.10 (m, 4H-**12+13+32+33**), 6.93 (d, *J* = 8.5 Hz, 4H-**Ar**), 6.81 (d, *J* = 8.5 Hz, 2H-**Ar**), 6.77 (d, *J* = 8.5 Hz, 2H-**Ar**), 5.11 – 5.00 (m, 6H-**16/22/36/42+16/22/36/42+16/22/36/42**), 4.97 (s, 2H-**16/22/36/42**), 4.62 (td, *J* = 7.5, 6.0 Hz, 1H-**3**), 3.84 (s, 3H-**27/47**), 3.83 (s, 3H-**27/47**), 3.75 (s, 3H-**21/41**), 3.74 (s, 3H-**1**),3.71 (s, 3H-**21/41**), 3.16 (m, 2H-**7**), 1.70 – 1.62 (m, 2H-**4**), 1.51 – 1.41 (m, 1H-**4**), 1.32 – 1.23 (m, 2H-**6**), 1.23 – 1.13 (m, 2H-**5**).

^{13}C NMR: (126 MHz, $CDCl_3$) δ: 172.9-**2**, 165.2-**29/19**, 165.2-**29/19**, 160.0-**20/26/40/46**, 159.9-**20/26/40/46**, 159.8-**20/26/40/46**, 159.8-**20/26/40/46**, 151.9-**17/23/37/43**, 151.9-**17/23/37/43**, 147.2-**17/23/37/43**, 147.0-**17/23/37/43**, 130.78-**19/25/39/45+19/25/39/45**, 130.6-**19/25/39/45+19/25/39/45**, 129.8-**18/24/38/44+18/24/38/44**, 129.6-**18/24/38/44+18/24/38/44**, 128.6-**14/15/34/35+14/15/34/35**, 128.5-**14/15/34/35+14/15/34/35**, 127.4-**10/30**, 126.6-**10/30**, 124.4-**13/33**, 124.3-**13/33**, 123.3-**11/31**, 123.3-**11/31**, 117.3-**12/32**, 117.1-**12/32**, 114.2-**19/25/39/45+19/25/39/45**, 114.1**-19/25/39/45**, 113.9-**19/25/39/45**, 76.1-**22/42**, 76.0-**22/42**, 71.2-**16/36**, 71.2-**16/36**, 55.5-**21/27/41/47+21/27/41/47**, 55.4-**21/27/41/47/1+21/27/41/47/1**, 55.3-**21/27/41/47**, 52.7-**3**, 52.3-**21/27/41/47**, 39.4-**7**, 31.8-**4**, 29.0-**6**, 23.2-**5**.

In some cases, peaks overlap and therefore are hidden by others. This is believed to be the case at:

- 55.4 ppm = two of **21**, **27**, **41** and **47** - which are undistinguishable
- 114.2 ppm = two of **19**, **25**, **39** and **45** - which are undistinguishable
- 129.8 and 129.6 ppm, each = two of **18**, **24**, **38**, **44** - which are undistinguishable
- 130.8 and 130.6 ppm, each = two of **19**, **25**, **39**, **45** - which are undistinguishable

NMR spectra are in agreement with literature reports.[9]

(2*S*)-2,6-Bis({2,3-bis[(4-methoxyphenyl)methoxy]phenyl}form-amido)hexanoic acid, 17

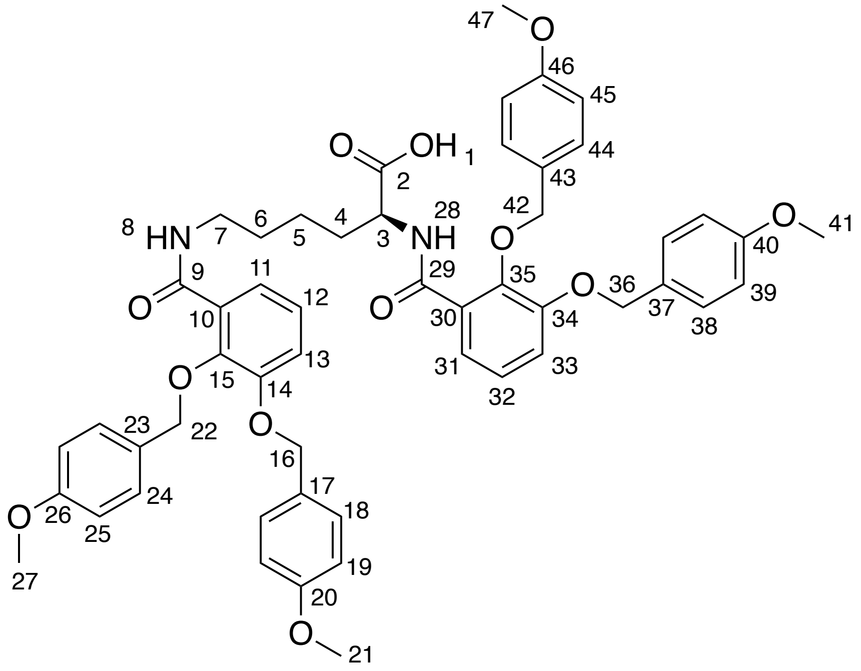

$C_{52}H_{54}N_2O_{12}$, 899.01 g mol^{-1}

(2*S*)-2,6-Bis({2,3-bis[(4-methoxyphenyl)methoxy]phenyl}formamido)hexanoic acid was prepared following a procedure from the literature.[9]

Yield: 742 mg, 0.825 mmol, 88%

Rf: 0.34 in dichloromethane : MeOH (9:1)

HRMS (ESI+):

Calc. for $[C_{52}H_{54}N_2O_{12}]+H^+$ = 899.3750, Found = 899.3765 (-1.7 ppm error, 14.5 mSigma)

Calc. for $[C_{52}H_{54}N_2O_{12}]+Na^+$ = 921.3569 Found = 921.3587 (-1.9 ppm error, 9.9 mSigma)

^{1}H NMR: (400 MHz, $CDCl_3$) δ: 8.64 (d, *J* = 7.0 Hz, 1H-**28**), 8.02 (t, *J* = 5.5 Hz, 1H-**8**), 7.69 (td, *J* = 7.0, 2.5 Hz, 2H-**11+31**), 7.38 (d, *J* = 8.5 Hz, 4H-**Ar**), 7.24 (d, *J* = 8.5 Hz, 2H-**Ar**), 7.19 (d, *J* = 8.5 Hz, 2H-**Ar**), 7.14 – 7.09 (m, 4H-**12+13+32+33**), 6.92 (d, *J* = 8.5 Hz, 4H-**Ar**), 6.80 (d, *J* = 8.5 Hz, 2H-**Ar**), 6.75 (d, *J* = 8.5 Hz, 2H-**Ar**), 5.04 (d, *J* = 3.5 Hz, 6H-**16/21/36/42+16/21/36/42+16/21/36/42**), 4.96 (s, 2H-**16/21/36/42**), 4.55 (q, *J* = 7.0 Hz, 2H-**3**), 3.83 (s, 6H-**27/47**), 3.73 (s, 3H-**21/41**), 3.71 (s, 3H-**21/41**), 3.15 (dh, *J* = 13.3, 6.7 Hz, 2H-**7**), 1.74 (dq, *J* = 13.0, 7.0, 6.0 Hz, 1H-**4**), 1.46 (dq, *J* = 14.4, 7.5 Hz, 1H-**4**), 1.33 – 1.13 (m, 4H-**5+6**).

^{13}C NMR: (126 MHz, $CDCl_3$) δ: 174.3-**2**, 166.2-**29/19**, 165.5-**29/19**, 160.0-**20/26/40/46**, 156.0-**20/26/40/46**, 159.8-**20/26/40/46**, 159.8-**20/26/40/46**, 151.9-**17/23/37/43**, 151.8-**17/23/37/43**, 147.3-**17/23/37/43**, 147.0-**17/23/37/43**, 130.9-**19/25/39/45+19/25/39/45**, 130.6-**19/25/39/45+19/25/39/45**, 129.8-**18/24/38/44+18/24/38/44**, 129.6-**18/24/38/44+18/24/38/44**, 128.6-**14/15/34/35**, 128.6-**14/15/34/35**, 128.5-**14/15/34/35**, 128.4-**14/15/34/35**, 127.1-**10/30**, 126.1-**10/30**, 124.4-**13**+**33**, 123.3-**11/31**, 123.2-**11/31**, 117.6-**12/32**, 117.2-**12/32**, 114.2-**19/25/39/45+19/25/39/45**, 114.1-**19/25/39/45**, 114.0-**19/25/39/45**, 76.2-**22/42**, 76.1-**22/42**, 71.2-**16/36**, 71.2-**16/36**, 55.4-**21/27/41/47+21/27/41/47**, 55.4-**21/27/41/47**, 55.3-**21/27/41/47**, 53.0-**3**, 39.5-**7**, 31.1-**4**, 29.0-**6**, 23.0-**5**.

In some cases, peaks overlap and therefore are hidden by others. This is believed to be the case at:

- 55.4 and 55.4 ppm, each = two of **21**, **27**, **41** and **47** - which are undistinguishable
- 114.2 ppm = two of **19**, **25**, **39** and **45** - which are undistinguishable
- 129.8 and 129.6 ppm, each = two of **18**, **24**, **38**, **44** - which are undistinguishable
- 130.9 and 130.6 ppm, each = two of **19**, **25**, **39**, **45** - which are undistinguishable

NMR spectra are in agreement with literature reports.[9]

(2*S*)-2,6-Bis({2,3-bis[(4-methoxyphenyl)methoxy]phenyl}formamido)-*N*-({[8-(prop-2-en-1-yloxy)quinolin-5-yl]carbamoyl}methyl)hexanamide, 18

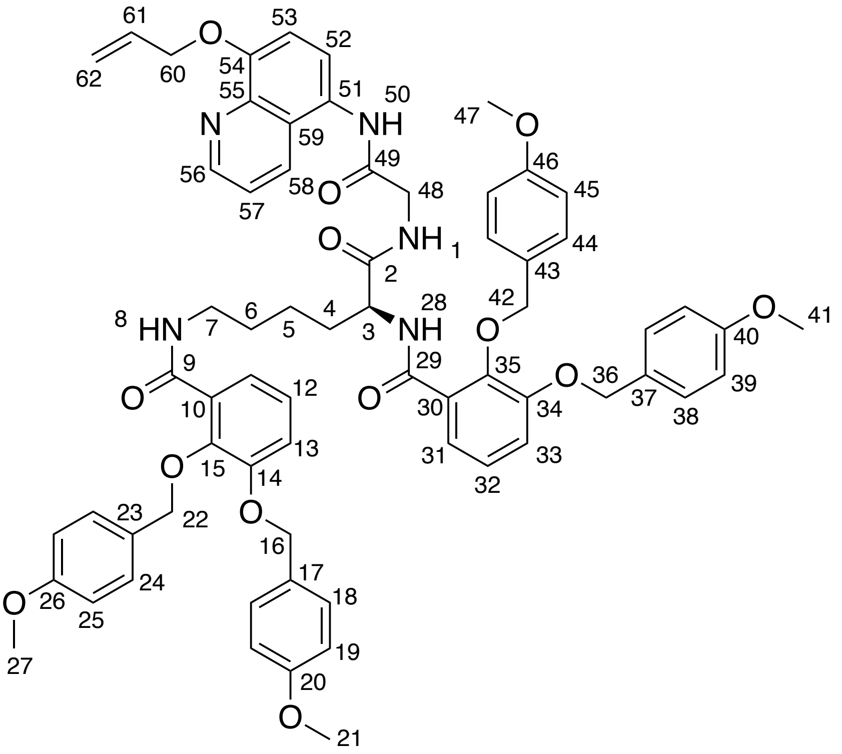

$C_{66}H_{67}N_5O_{13}$, 1138.28 g mol^{-1}

(2*S*)-2,6-bis({2,3-bis[(4-Methoxyphenyl)methoxy] phenyl}formamido)-*N*-({[8-(prop-2-en-1-yloxy)quinolin-5-yl]carbamoyl}methyl)hexanamide was prepared based on a procedure from the literature.[9]

To a solution of (2*S*)-2,6-bis({2,3-bis[(4-methoxyphenyl)methoxy]phenyl}formamido)hexanoic acid (299 mg, 0.355 mmol) in dry DMF (5 mL) was added *N'*-ethylcarbodiimide hydrochloride (102 mg, 0.533 mmol), hydroxybenzotriazole monohydrate (82 mg, 0.522 mmol) and DIPEA (216 μL, 1.24 mmol), and then 5-(2-azaniumylacetamido)-8-(prop-2-en-1-yloxy)quinolin-1-ium ditrifluoroacetate (172 mg, 0.355 mmol). The resulting solution was left stirring for 18 h at room temperature and after this time, the reaction solvent was removed *in vacuo*. The produced dark purple residue was re-dissolved in dichloromethane (30 mL) and washed with saturated NH_4Cl(aq) (30 mL, x3) and then saturated $NaHCO_3$(aq) (30 mL, x3). The organic layer

solvent was removed *in vacuo* to leave an orange/brown foam. This solid was purified *via* column chromatography, eluting with dichloromethane : MeOH : NEt_3 (100:3:1), and then triturated in Et_2O, to yield the title compound as a white solid.

Yield: 222 mg, 0.195 mmol, 55%

Rf: 0.17 in dichloromethane : MeOH : NEt_3 (100:3:1)

HRMS (ESI+):

Calc. for $[C_{66}H_{67}N_5O_{13}]+H^+$ = 1138.4808, Found = 1138.4862 (-4.8 ppm error, 5.3 mSigma)

Calc. for $[C_{66}H_{67}N_5O_{13}]+Na^+$ = 1160.4628 Found = 1160.4691 (-5.4 ppm error, 4.2 mSigma)

^{1}H NMR: (400 MHz, $CDCl_3$) δ 9.18 (s, 1H-**50**), 8.85 (dd, *J* = 4.0, 1.0 Hz, 1H-**56**), 8.61 (d, *J* = 4.5 Hz, 1H-**28**), 8.25 (d, *J* = 8.5 Hz, 1H-**58**), 8.07 (t, *J* = 5.5 Hz, 1H-**8**), 7.60 (dd, *J* = 7.5, 2.0 Hz, 1H-**11/31**), 7.56 (d, *J* = 8.5 Hz, 1H-**11/31**), 7.38 (dd, *J* = 8.5, 2.0 Hz, 4H-**Ar**), 7.31 (d, *J* = 8.5 Hz, 2H-**Ar**), 7.21 (d, *J* = 8.5 Hz, 2H-**Ar**), 7.17-7.05 (m, 6H-**57**+**12/32+13+33+52/53**), 7.03 (d, *J* = 8.5 Hz, 1H-**12/32**), 6.93 (d, *J* = 8.5 Hz, 4H-**Ar**), 6.87 (d, *J* = 8.0 Hz, 1H-**52/53**), 6.84 – 6.78 (m, 4H-**Ar**), 6.25 – 6.12 (m, 1H-**61**), 5.46 (dd, *J* = 17.0, 1.5 Hz, 1H-**62(trans)**), 5.32 (dd, *J* = 10.5, 1.5 Hz, 1H-**62(cis)**), 5.06 (s, 6H-**16/21/36/42+16/21/36/42+16/21/36/42**), 4.98 (s, 2H-**16/21/36/42**), 4.83 (d, *J* = 5.5 Hz, 2H-**60**), 4.22 (dd, *J* = 17.0, 7.0 Hz, 1H-**48**), 4.17-4.07 (m, 1H-**3**), 3.99 (dd, *J* = 17.0, 5.5 Hz, 1H-**48**), 3.82 (d, 6H-**27+47**), 3.75 (s, 3H-**21/41**), 3.71 (s, 3H-**21/41**), 3.21 (q, *J* = 6.5 Hz, 2H-**7**), 1.68 (dq, *J* = 13.5, 7.0, 6.5 Hz, 1H-**4**), 1.39 (dt, *J* = 14.0, 7.5 Hz, 1H-**4**), 1.34 – 1.26 (m, 2H-**6**), 1.20 (q, *J* = 6.5, 5.5 Hz, 2H-**5**).

^{13}C NMR: (126 MHz, $CDCl_3$) δ: 173.2-**2**, 169.1-**49**, 166.8-**29/19**, 165.5-**29/19**, 160.2-**20/26/40/46**, 160.0-**20/26/40/46**, 159.8-**20/26/40/46**, 159.8-**20/26/40/46**, 151.9-**17/23/37/43+54**, 151.7-**17/23/37/43**, 148.5-**56**, 147.2-**17/23/37/43**, 146.9-**17/23/37/43**, 133.1-**58**, 130.9-**19/25/39/45+19/25/39/45**, 130.6-**19/25/39/45+19/25/39/45**, 129.7-**18/24/38/44+18/24/38/44**, 129.7-**18/24/38/44+18/24/38/44**, 128.7-**14/15/34/35**, 128.5-**14/15/34/35**, 128.4-**14/15/34/35**, 128.3-**14/15/34/35**, 127.0-**51/55**, 125.7-**51/55**, 125.4-

10/30, 125.2-**10/30**, 124.5-**59**, 124.3-**13/33**, 124.2-**13/33**, 123.1-**11/31**, 122.8-**11/31**, 121.5-**57**, 118.7-**62**, 117.8-**12/32**, 117.1-**12/32**, 114.2-**19/25/39/45**, 114.2-**19/25/39/45**, 114.2-**19/25/39/45+19/25/39/45**, 76.2-**22/42**, 76.1-**22/42**, 71.2-**16/36**, 71.2-**16/36**, 70.2-**60**, 55.5-**21/27/41/47**, 55.5-**21/27/41/47+21/27/41/47**, 55.4-**21/27/41/47**, 44.0-**62**, 39.0-**7**, 30.2-**4**, 28.9-**6**, 23.1-**5**.

In some cases, peaks overlap and therefore are hidden by others. This is believed to be the case at:

- 55.5 = two of **21**, **27**, **41** and **47** - which are undistinguishable
- 114.2 ppm = two of **19**, **25**, **39** and **45** - which are undistinguishable
- 129.7 and 129.7 ppm, each = two of **18**, **24**, **38**, **44** - which are undistinguishable
- 130.9 and 130.6 ppm, each = two of **19**, **25**, **39**, **45** - which are undistinguishable

NMR spectra are in agreement with literature reports.[9]

IR ATIR (cm^{-1}): 3351 m br (N-H stretch), 3270 m br (N-H stretch), 3066 w (Aromatic/Alkene C-H stretch), 2930 m (Alkane C-H stretch), 2871 m (Alkane C-H stretch), 2838 m (Alkane C-H stretch), 1642 m (C=O stretch), 1611 (Alkene C=C stretch), 1513 (Aromatic C=C stretch).

Elemental Analysis:

Calculated for [$C_{66}H_{67}N_5O_{13}$ • 0.4 CH_2Cl_2 • 0.15 CH_4O]: %C 67.91, %H 5.86, %N 5.95

Measured for [$C_{66}H_{67}N_5O_{13}$ • 0.4 CH_2Cl_2 • 0.15 CH_4O]: %C 68.09, %H 5.85, %N 5.77

Melting Point: 78-80 °C

2,6-Bis[(2,3-dihydroxyphenyl)formamido]-*N*-({[8-(prop-2-en-1-yloxy)quinolin-5-yl]carbamoyl}methyl)hexanamide, 19

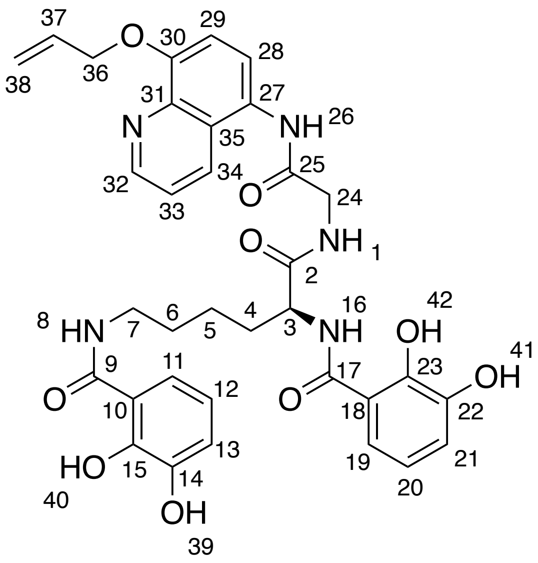

$C_{34}H_{35}N_5O_9$, 657.68 g mol^{-1}

To a stirred solution of (2*S*)-2,6-bis({2,3-bis[(4-methoxyphenyl)methoxy]-phenyl} formamido)-*N*-({[8-(prop-2-en-1-yloxy)quinolin-5-yl]carbamoyl}methyl)hexanamide (222 mg, 195 μmol) in dioxane (9 mL) was added 4 N HCl in dioxane (2.2 mL) dropwise and left stirring for 5 h at room temperature. After this time, the reaction solvent was removed *in vacuo* and the produced residue was purified *via* column chromatography, eluting with 9 : 1 dichloromethane : MeOH, and then triturated in Et_2O, to yield the title compound as a beige solid.

Yield: 30 mg, 65.4 μmol, 34%

Rf: 0.44 in dichloromethane : MeOH (8:2)

HRMS (ESI-):

Calc. for $[C_{34}H_{35}N_5O_9]-H^+$ = 656.2362, Found = 656.2373 (-1.7 ppm error, 6.7 mSigma)

^{1}H NMR: (400 MHz, MeOD-*d4*) δ: 8.77 – 8.68 (m, 1H-**32**), 8.33 (d, *J* = 8.5 Hz, 1H-**34**), 7.46 (d, *J* = 8.5 Hz, 1H-**28/29**), 7.41 (dd, *J* = 8.5, 4.5 Hz, 1H-**33**), 7.26 – 7.21 (m, 1H-**11/19**), 7.16 – 7.10 (m, 2H-**11/19+28/29**), 6.92 – 6.86 (m, 2H-**13+21**), 6.64 (td, *J* = 8.0,

4.0 Hz, 2H-**12+20**), 6.19 (ddt, *J* = 16.0, 10.5, 5.5 Hz, 1H-**37**), 5.47 (dd, *J* = 17.5, 1.5 Hz, 1H-**38(trans)**), 5.30 (dd, *J* = 10.5, 1.5 Hz, 1H-**38(cis)**), 4.80 (d, *J* = 5.0 Hz, 2H-**36**), 4.54 (t, *J* = 7.0 Hz, 1H-**3**), 4.25 – 4.05 (m, 2H-**24**), 3.37 (t, *J* = 6.5 Hz, 2H-**7**), 2.08 – 1.84 (m, 2H-**4**), 1.71 – 1.62 (m, 1H-**6**), 1.60 – 1.47 (m, 2H-**5**).

^{13}C NMR: (126 MHz, MeOD-*d*$_4$) δ: 175.6-**2**, 171.6-**9/17/25**, 171.5-**9/17/25**, 171.4-**9/17/25**, 153.9-**30**, 150.2-**15/23**, 149.7-**32**, 149.5-**15/23**, 147.3-**14/22**, 147.1-**14/22**, 140.5-**27**, 134.4-**37**, 134.0-**34**, 130.9-**15/23**, 130.7-**15/23**, 127.2-**35**, 126.2-**31**, 126.0-**28/29**, 123.0-**33**, 119.9-**12/20/13/21**, 119.8-**12/20/13/21**, 119.8-**12/20/13/21**, 119.5-**12/20/13/21**, 118.6-**11/19**, 118.4-**11/19**, 116.9-**10/18**, 116.7-**10/18**, 110.2-**28/29**, 70.9-**36**, 55.8-**3**, 44.2-**24**, 40.1-**7**, 32.1-**4**, 30.0-**6**, 24.3-**5**.

IR ATIR (cm^{-1}): 3332 w br (O-H stretch), 3046 w (Aromatic/Alkene C-H stretch), 2943 w (Alkane C-H stretch), 1638 s (C=O stretch), 1587 s (Alkene C=C stretch), 1535 s (Aromatic C=C stretch), 1504 s (Aromatic C=C stretch).

Elemental Analysis:

Calculated for [$C_{34}H_{35}N_5O_9$ • 0.05 CH_2Cl_2 • 1.1 $C_4H_{10}O$]: %C 62.12, %H 6.25, %N 9.42

Measured for [$C_{34}H_{35}N_5O_9$ • 0.05 CH_2Cl_2 • 1.1 $C_4H_{10}O$]: %C 62.35, %H 5.82, %N 9.01

Melting Point: 157-159 °C

Ru-s4

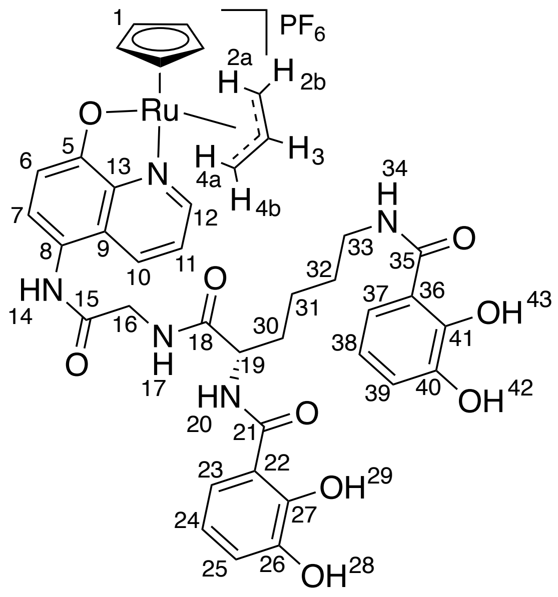

$C_{39}H_{40}F_6N_5O_9PRu$, 968.809 g mol^{-1}

To a stirred solution of tris(acetonitrile)cyclopentadienylruthenium(II) hexafluorophosphate (12.85 mg, 29.6 μmol) in dry, degassed DMF (0.5 mL) was added 2,6-bis[(2,3-dihydroxyphenyl)formamido]-*N*-({[8-(prop-2-en-1-yloxy)quinolin-5-yl]carbamoyl}methyl)hexanamide (24.32 mg, 29.6 μmol) as a solution in dry, degassed DMF (3 mL) under a dry, nitrogen atmosphere at room temperature to form, at first a dark red solution which turned to a light red solution over 2 min. After stirring for 30 min under these conditions, the ruthenium-complex was precipitated using dry Et_2O (45 mL). The precipitate was re-dissolved in dry DMF (5 mL) and precipitated using dry Et_2O (15 mL). The produced solid was washed with more dry Et_2O (20 mL, x3) and then dry dichloromethane (10 mL x3), to yield the title compound as a dark brown solid.

Yield: 12.2 mg, 12.6 μmol, 43%

HRMS (ESI+):

Calc. for $[C_{36}H_{35}N_5O_9Ru]+Na^+$ = 806.1370, Found = 806.1408 (-3.3 ppm error, 65.0 mSigma)

Calc. for $[C_{39}H_{40}N_5O_9Ru]^+$ = 656.2362, Found = 656.2373 (-3.0 ppm error, 22.7 mSigma)

^{1}H NMR: (500 MHz, DMSO-*d6*) δ: 12.81 (s, 1H-**29/43**), 11.85 (d, *J* = 10.5 Hz, 1H-**29/43**), 9.60 (d, *J* = 8.0 Hz, 1H-**14**), 9.27 (br s, 1H-**28/42**), 9.09 (br s, 1H-**28/42**), 8.86 (s, 1H-**12**), 8.82 (d, *J* = 6.5 Hz, 1H-**20**), 8.75 (s, 1H-**34**), 8.57 – 8.50 (m, 1H-**17**), 8.37 – 8.31 (m, 1H-**10**), 7.55 (d, *J* = 8.0 Hz, 1H-**11**), 7.40 (d, *J* = 7.0 Hz, 1H-**23**), 7.26 (d, *J* = 9.0 Hz, 1H-**7**), 7.24 (d, *J* = 8.0 Hz, 1H-**37**), 6.93 (d, *J* = 8.0 Hz, 1H-**25**), 6.89 (d, *J* = 8.0 Hz, 1H-**39**), 6.86 (d, *J* = 8.5 Hz, 1H-**6**), 6.69 (dt, *J* = 8.0, 4.0 Hz, 2H-**24**), 6.64 (t, *J* = 8.0 Hz, 1H-**38**), 6.25 (s, 5H-**1**), 4.58 – 4.41 (m, 3H-**2a/4a+3+19**), 4.25 (d, *J* = 9.0 Hz, 1H-**2a/4a**), 4.09 (s, 1H-**2b/4b**), 4.04 – 3.93 (d, *J* = 17.5 Hz, 3H-**2b/4b+16**), 3.34 (s, 2H-**33**), 3.29 – 3.17 (m, 1H-**33**), 1.92 – 1.74 (m, 2H-**30**), 1.62 – 1.52 (m, 2H-**32**), 1.50 – 1.30 (m, 2H-**31**).

^{13}C NMR: (151 MHz, DMSO-*d6*) δ: 172.2-**18**, 169.7-**35**, 169.1-**21**, 168.8-**15**, 167.3-**5**, 155.6-**12**, 149.7-**41**, 148.7-**27**, 146.2-**22/36**, 146.0-**22/36**, 144.8-**13**, 135.9-**10**, 126.7-**7**, 126.0-**9**, 123.2-**11**, 118.9-**25/39**, 118.7-**25/39**, 118.7-**8**, 118.4-**23**, 118.1-**24**, 117.9-**38**, 117.0-**37**, 115.6-**26**, 114.9-**40**, 113.8-**6**, 98.0-**3**, 95.8-**1**, 68.2-**2/4**, 63.1-**2/4**, 53.4-**19**, 42.6-**16**, 38.8-**7**, 31.1-**30** 28.6-**32**, 23.1-**31**.

^{19}F NMR: (471 MHz, DMSO-*d6*) δ: -70.2 (d, *J* = 711.0 Hz, 6F).

^{31}P NMR: (202 MHz, DMSO-*d6*) δ: -146.0 (d, *J* = 711.0 Hz, 1P).

IR ATIR (cm^{-1}): 3507 w br (O-H stretch), 3325 m br (Amide N-H stretch), 3111 w (Aromatic/Alkene C-H stretch), 2939 w (Alkane C-H stretch), 2868 w (Alkane C-H stretch), 1640 s (C=O stretch), 1588 s (Alkene C=C stretch), 1532 s (Aromatic C=C stretch).

Elemental Analysis:

Calculated for $[C_{39}H_{40}F_6N_5O_9PRu \cdot 1.0\ CH_2Cl_2 \cdot 0.5\ C_4H_{10}O]$: %C 46.25, %H 4.34, %N 6.42

Measured for $[C_{39}H_{40}F_6N_5O_9PRu \cdot 1.0\ CH_2Cl_2 \cdot 0.5\ C_4H_{10}O]$: %C 46.55, %H 4.04, %N 6.15

UV-vis Spectroscopy:

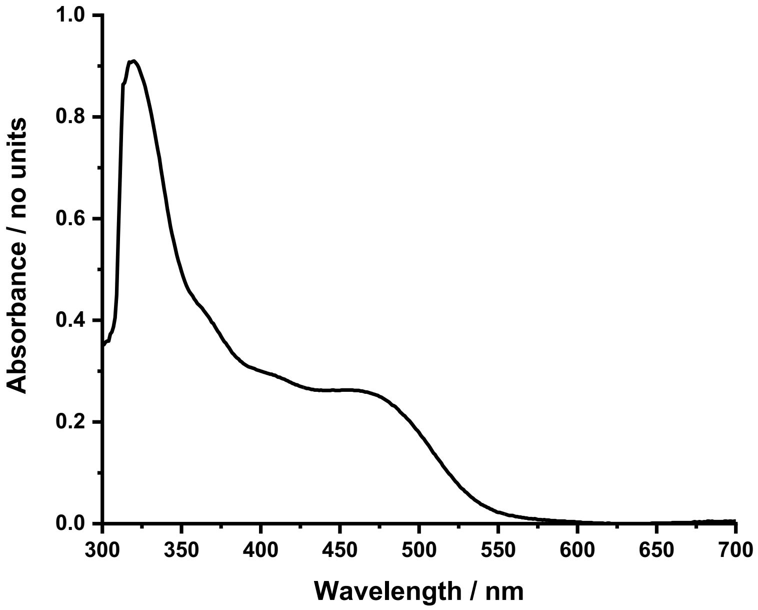

Figure S 7 UV-vis spectrum, 200 μM in DMSO.

2.9. The Synthesis of Ru-s5

N-{5-[(2-Ethoxy-3,4-dioxocyclobut-1-en-1-yl)amino]pentyl}-*N*-hydroxy-*N'*-[5-(*N*-hydroxy-3-{[5-(*N*-hydroxyacetamido)pentyl]carbamoyl}propanamido)pentyl]butanediamide, 20

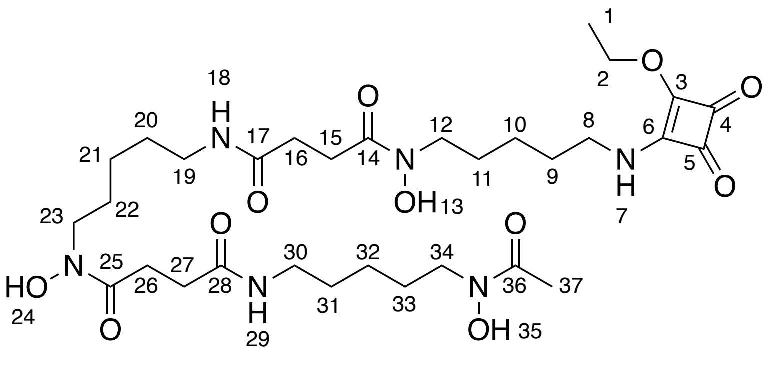

$C_{31}H_{52}N_6O_{11}$, 684.788 g mol^{-1}

N-{5-[(2-Ethoxy-3,4-dioxocyclobut-1-en-1-yl)amino]pentyl}-*N*-hydroxy-*N'*-[5-(*N*-hydroxy-3-{[5-(*N*-hydroxyacetamido)pentyl]carbamoyl}propanamido)pentyl]butanediamide was prepared based on a procedure from the literature.[10]

A white suspension of desferrioxamine mesylate (492 mg, 0.749 mmol) and DIPEA (131 μL) in dry EtOH (20 mL) was heated to 50 °C for 1 h. After this time, 2,3-diethoxy-3-cyclobutene-1,2-dione (333 μL, 2.25 mmol) was added and the resulting mixture was left to stir at 50 °C for 18 h. Reaction solvent was then removed *in vacuo* and washed with room temperature EtOH (20 mL, x3), to yield the title compound as a fluffy white solid.

Yield: 488 mg, 0.713 mmol, 95%

Rf: 0.13 in dichloromethane : MeOH (9:1)

HRMS (ESI+):

Calc. for $[C_{31}H_{52}N_6O_{11}]+H^+$ = 685.3767 Found = 685.3769 (-0.3 ppm error, 25.2 mSigma)

Calc. for $[C_{31}H_{52}N_6O_{11}]+Na^+$ = 707.3586 Found = 707.3571 (2.1 ppm error, 5.7 mSigma)

^{1}H NMR: (400 MHz, DMSO-*d*$_6$) δ: 9.84-9.44 (m, 3H-**13+24+35**), 8.80 (t, *J* = 5.0 Hz, 1H-**7(rotamer 1)**), 8.60 (t, *J* = 5.5 Hz, 1H-**7(rotamer 2)**), 7.82 – 7.75 (m, 2H-**18**+**29**), 4.71 – 4.58 (m, 2H-**2**), 3.45 (t, *J* = 7.0 Hz, 6H-**12**+**23**+**34+8(rotamer 2)**), 3.26 (q, *J* = 6.5 Hz, 2H-**8(rotamer 1)**), 2.99 (q, *J* = 7.0 Hz, 4H-**19**+**30**), 2.57 (t, *J* = 7.5 Hz, 4H-**15**+**26**), 2.26 (t, *J* = 7.5 Hz, 4H-**16**+**27**), 1.96 (s, 3H-**37**), 1.50 (q, *J* = 7.5 Hz, 8H-**9**+**11**+**22**+**33**), 1.37 (m, 7H-**1**+**20**+**31**), 1.22 (m, 6H-**10**+**21**+**32**).

For this compound, it is believed two rotamers exist. The only two environments that change in magnetic environment between these rotamers are **7** and **8**.

NMR spectra of this compound in literature reports are incorrectly assigned. Despite this, comparison of the spectra obtained in this study to that of Rudd *et al.* 2016, suggests the desired compound was successfully obtained.[10]

N-[5-({3,4-Dioxo-2-[({[8-(prop-2-en-1-yloxy)quinolin-5-yl]carbamoyl}methyl)amino]cyclobut-1-enz-1-yl}amino)pentyl]-*N*-hydroxy-*N'*-[5-(*N*-hydroxy-3-{[5-(*N*-hydroxyacetamido)pentyl]carbamoyl}propanamido)pentyl]butanediamide, 21

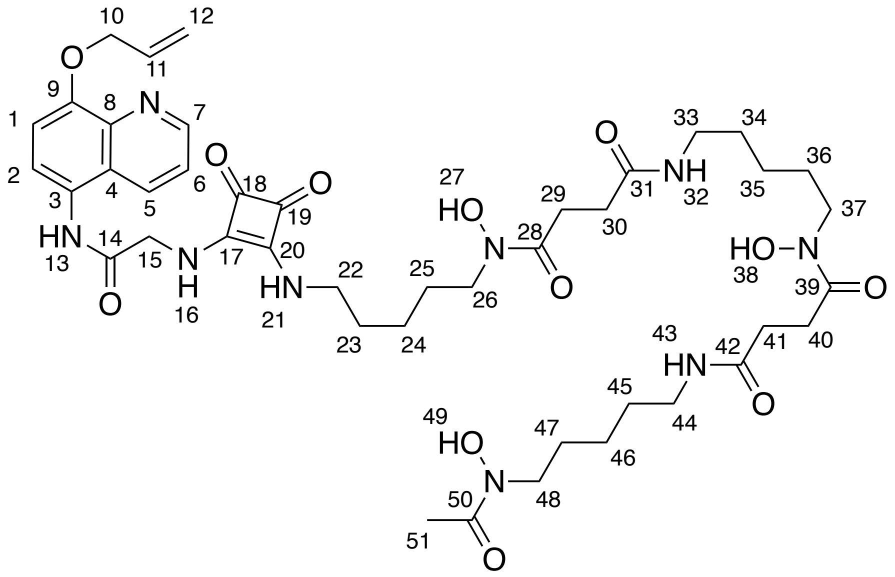

$C_{43}H_{61}N_9O_{12}$, 896.012 g mol^{-1}

To a pale yellow/orange solution of *N*-{5-[(2-ethoxy-3,4-dioxocyclobut-1-en-1-yl)amino]pentyl}-*N*-hydroxy-*N'*-[5-(*N*-hydroxy-3-{[5-(*N*-hydroxyacetamido)pentyl]carbamoyl}propanamido)pentyl]butanediamide (80 mg, 0.123 mmol) and DIPEA (107 μL, 0.614 mmol) in a solvent mixture of EtOH : H_2O (6 mL, 4:1) was added 5-(2-azaniumylacetamido)-8-(prop-2-en-1-yloxy)quinolin-1-ium ditrifluoroacetate, (114 mg, 0.235 mmol) in EtOH (4 mL). After stirring for 24 h at room temperature, reaction solvent was removed *in vacuo*. The resulting residue was re-suspended in MeCN (40 mL), where the produced supernatant was decanted to leave the solid. This solid was washed with further MeCN (20 mL, x4), to yield the title compound as a light brown solid.

Yield: 85 mg, 94.9 μmol, 77%

HRMS (ESI+):

Calc. for $[C_{43}H_{61}N_9O_{12}]+H^+$ = 896.4512 Found = 896.4531 (-2.1 ppm error, 30.2 mSigma)

Calc. for $[C_{43}H_{61}N_9O_{12}]+Na^+$ = 918.4332 Found = 918.4361 (-3.2 ppm error, 39.5 mSigma)

^{1}H NMR: (400 MHz, DMSO-*d*$_6$) δ: 10.10 (br s, 1H-**13**), 9.77 – 9.46 (m, 3H-**27**+**38**+**49**), 8.88 (d, *J* = 4.0 Hz, 1H-**7**), 8.39 (d, *J* = 8.5 Hz, 1H-**5**), 7.79 (t, *J* = 5.5 Hz, 2H-**32**+**43**), 7.67 (br s, 1H-**16/21**), 7.58 (dd, *J* = 8.5, 4.0 Hz, 1H-**6**), 7.52 (d, *J* = 8.5 Hz, 1H-**1**), 7.21 (d, *J* = 8.5 Hz, 1H-**2**), 6.16 (ddt, *J* = 16.0, 10.5, 5.5 Hz, 1H-**11**), 5.51 (d, *J* = 17.5 Hz, 1H-**12**(**trans)**), 5.32 (d, *J* = 10.5 Hz, 1H-**12(cis)**), 4.77 (d, *J* = 5.5 Hz, 2H-**10**), 4.56 (br s, 2H-**16/21**), 3.56 – 3.42 (m, 10H-**22+15**+**26**+**37**+**48**), 2.99 (q, *J* = 6.5 Hz, 4H-**33**+**44**), 2.57 (t, *J* = 7.5 Hz, 4H-**29**+**40**), 2.26 (t, *J* = 7.5 Hz, 4H-**30**+**41**), 1.96 (s, 3H-**51**), 1.59 – 1.43 (m, 8H-**23**+**25**+**36**+**46**), 1.43 – 1.32 (m, 4H-**34**+**45**), 1.32 – 1.16 (m, 6H-**24**+**35**+**47**).

^{13}C NMR: (126 MHz, DMSO-*d*$_6$) δ: 182.6-**18**+**19**, 172.0-**28**+**39**, 171.4-**14**+**31**+**42**, 170.2-**50**, 152.2-**9**, 149.1-**7**, 139.8-**3**, 133.7-**11**, 131.7-**5**, 125.52-**4**, 124.8-**8**, 123.2-**2**, 121.6-**6**, 117.8-**12**, 109.3-**1**, 69.2-**10**, 47.1-**17+20**, 47.1-**15**, 46.8-**15**+**26**+**37**+**48+17+20**, 46.1-**15**+**26**+**37**+**48+17+22**, 43.3-**22**, 38.4-**33**+**44**, 30.4 **23**+**25**+**36**+**47**, 29.9-**30**+**41**, 28.8-**34+45**, 27.6-**29**+**40**, 26.0-**23**+**25**+**36**+**47**, 23.5-**24**+**35**+**46**, 23.0-**24**+**35**+**46**, 20.4-**51**.

In some cases, different peaks that correspond to numerous environments are undistinguishable and therefore the number of environments that correspond to each is unknown. This is the case at:

- 23.0 ppm = one or two of **34**, **35**, and **46** - which are undistinguishable
- 23.5 ppm = one or two of **34**, **35**, and **46** - which are undistinguishable
- 46.1 ppm = one, two, three, four or five of **15**, **26**, **37**, **48**, **17**, and **20** - which are undistinguishable
- 46.8 ppm = one, two, three, four or five of **15**, **26**, **37**, **48**, **17**, and **20** - which are undistinguishable

IR ATIR (cm^{-1}): 3225 m br (O-H stretch), 3091 m (Aromatic/Alkene C-H stretch), 2928 m (Alkane C-H stretch), 2860 m (Alkane C-H stretch), 1803 w (Alkene C-H stretch), 1644 s (C=O stretch), 1574 s (Alkene C=C stretch), 1550 s (Aromatic C=C stretch).

Elemental Analysis:

Calculated for [$C_{43}H_{61}N_9O_{12}$ • 1.15 H_2O]: %C 56.34, %H 6.96, %N 13.75

Measured for [$C_{43}H_{61}N_9O_{12}$ • 1.15 H_2O]: %C 56.48, %H 6.57, %N 13.62

Melting Point: 155 – 157 °C

UV-vis Spectroscopy:

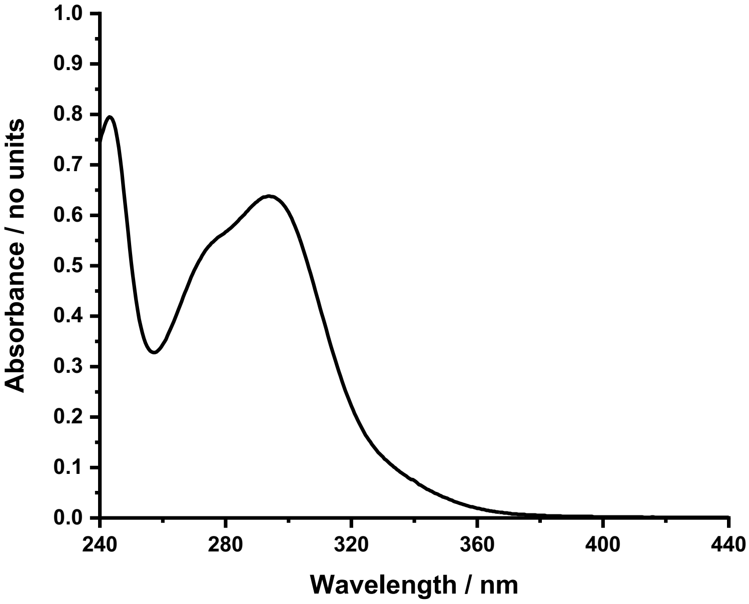

Figure S 8 UV-vis spectrum, 25 μM in 10% DMSO in water.

HPLC:

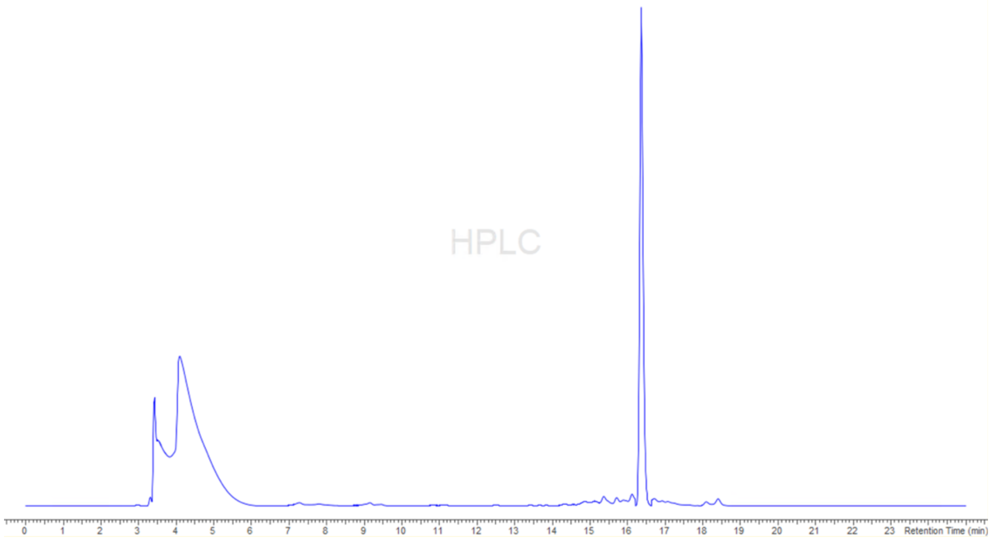

Figure S 9 HPLC chromatogram at 254 nm, 10 μL injection, 40 °C oven temperature, eluted with 1-20% MeCN in water over 25 min. Rt = 16.4 min, 94% purity. Ignore absorbances between 3.0-5.5 min due to solvent.

Ru-s5

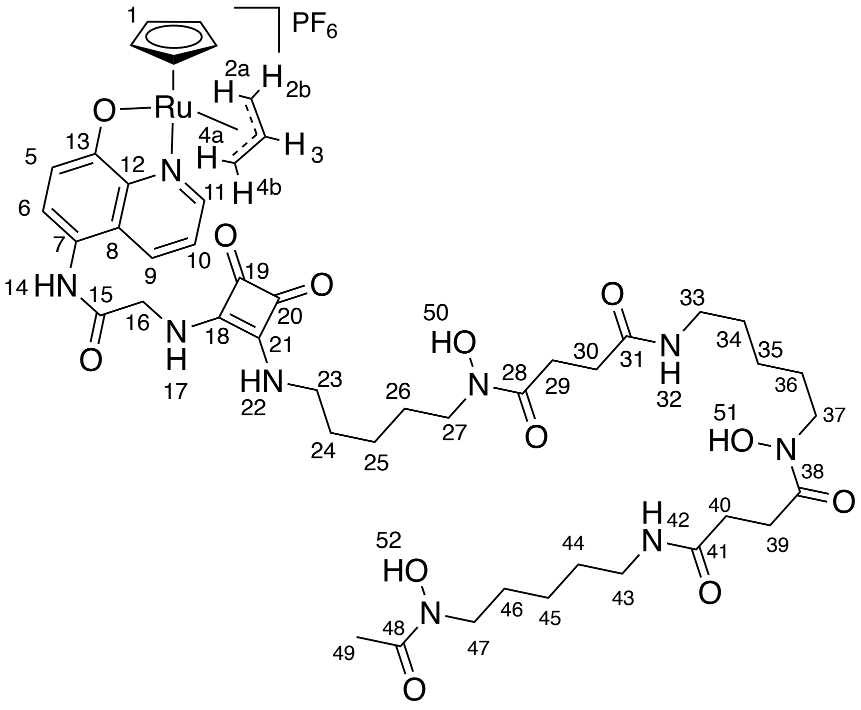

$C_{48}H_{66}F_6N_9O_{12}PRu$, 1207.1412 g mol^{-1}

To a stirred solution of tris(acetonitrile)cyclopentadienylruthenium(II) hexafluorophosphate (15.50 mg, 35.7 μmol) in dry, degassed DMF (0.5 mL) was added *N*-[5-({3,4-dioxo-2-[({[8-(prop-2-en-1-yloxy)quinolin-5-yl]carbamo-yl}methyl) amino]cyclobut-1-enz-1-yl}amino)pentyl]-*N*-hydroxy-*N'*-[5-(*N*-hydroxy-3-{[5-(*N*-hyd-roxyacetamido)pentyl]carbamoyl}propanamido)pentyl]butanediamide (27.19 mg, 29.7 μmol) as a solution in dry, degassed DMF (19 mL) under a dry, nitrogen atmosphere at room temperature to form, a dark red solution. After stirring for 2 h under these conditions, the ruthenium-complex was precipitated using dry Et_2O (45 mL) and the resulting supernatant decanted to leave a dark orange solid. This solid was then re-suspended in dry DMF (5 mL). The resulting supernatant was collected and the complex precipitated using dry Et_2O (45 mL). This precipitate was then washed with further dry Et_2O (20 mL, x3) and then dry dichloromethane (10 mL x3), to yield the title compound as a dark brown solid.

Yield: 16.7 mg, 13.8 μmol, 47%

HRMS (ESI+):

Calc. for $[C_{47}H_{60}N_9O_{12}PRu]^+$ = 1044.3399 Found = 1044.3434 (-2.0 ppm error, 90.6 mSigma)

Calc. for $[C_{48}H_{66}N_9O_{12}Ru]^+$ = 1062.3839 Found = 1062.3891 (-0.8 ppm error, 49.3 mSigma)

Calc. for $[C_{48}H_{65}N_9O_{12}PRu]+Na^+$ = 1084.3688 Found = 1084.3687 (1.4 ppm error, 173.5 mSigma)

^{1}H NMR: (500 MHz, DMSO-d*6*) δ: 9.95 (s, 1H-**14**), 9.69 – 9.45 (m, 3H-**50+51+52**), 8.90 (d, *J* = 4.5 Hz, 1H-**11**), 8.44 (d, *J* = 8.5 Hz, 1H-**9**), 7.76 (t, *J* = 5.0 Hz, 2H-**32+42**), 7.69 – 7.48 (m, *J* = 17.5, 9.0 Hz, 1H-**10**), 7.30 (d, *J* = 8.5 Hz, 1H-**5**), 6.88 (d, *J* = 8.5 Hz, 1H-**6**), 6.25 (s, 5H-**1**), 4.51 – 4.43 (m, 2H-**3+2a/4a**), 4.25 (d, *J* = 9.5 Hz, 1H-**2a/4a**), 4.14 – 4.08 (m, 1H-**2b/4b**), 4.05 – 3.99 (m, 1H-**2b/4b**), 3.52 – 3.41 (m, 10H-**16+23+27+37+47**), 2.99 (dd, *J* = 13.0, 6.5 Hz, 4H-**33+43**), 2.57 (t, *J* = 7.0 Hz, 4H-**29+39**), 2.26 (t, *J* = 7.5 Hz, 4H-**30+40**), 1.96 (s, 3H-**49**), 1.56 – 1.45 (m, 1H-**24+26+36+46**), 1.42 – 1.33 (m, 4H-**34+44**), 1.31 – 1.18 (m, 6H-**25+35+45**).

^{13}C NMR: (151 MHz, DMSO-*d*$_6$) δ: 182.6-**19+20**, 172.0-**28+38**, 171.3-**15+31+42**, 170.1-**48**, 167.3-**13**, 155.7-**11**, 144.7-**8**, 136.0-**9**, 126.7-**5**, 125.8-**12**, 123.3-**10**, 118.6-**7**, 113.8-**6**, 98.0-**3**, 95.8-**1**, 68.2-**2/4**, 63.1-**2/4**, 47.1-**18+21**, 46.8-**16+27+37+47+18+21**, 45.9-**16+27+37+47+18+21**, 43.2-**23**, 38.4-**33+43**, 30.4-**24+26+37+47**, 29.9-**30+40**, 28.8-**34+44**, 27.5-**29+39**, 26.0-**24+26+37+47**, 23.5-**25+35+46**, 23.0-**25+35+46**, 20.3-**49**.

The additional peak at 85.6 ppm is due to the cyclopentadienyl environment of the active Ru(II) intermediate species that is presumed to form *in situ* on reaction with residual water in the NMR solvent.

In some cases, different peaks that correspond to numerous environments are undistinguishable and therefore the number of environments that correspond to each is unknown. This is the case at:

- 23.0 ppm = one or two of **25**, **35**, and **46** - which are undistinguishable
- 23.5 ppm = one or two of **25**, **35**, and **46** - which are undistinguishable
- 45.9 ppm = one, two, three, four or five of **16**, **27**, **37**, **47**, **18**, and **21** - which are undistinguishable
- 46.8 ppm = one, two, three, four or five of **16**, **27**, **37**, **47**, **18**, and **21** - which are undistinguishable

^{19}F NMR: (471 MHz, DMSO-*d*$_6$) δ: -70.2 (d, *J* = 711.0 Hz, 6F).

^{31}P NMR: (202 MHz, DMSO-*d*$_6$) δ: -142.4 (d, *J* = 711.0 Hz, 1P).

IR ATIR (cm^{-1}): 3361 m br (Alcohol O-H stretch), 3276 m br (Amide N-H stretch), 2928 m (Alkane C-H stretch), 2860 m (Alkane C-H stretch), 1803 w (Alkene C-H stretch), 1649 s (C=O stretch), 1622 s (C=O stretch), 1587 (Alkene C=C stretch), 1560 (Aromatic C=C stretch).

Elemental Analysis:

Calculated for [$C_{48}H_{66}F_6N_9O_{12}PRu$ • 1.5 CH_2Cl_2]: %C 45.24, %H 5.28, %N 9.66
Measured for [$C_{48}H_{66}F_6N_9O_{12}PRu$ • 1.5 CH_2Cl_2]: %C 45.40, %H 5.10, %N 9.50

UV-vis Spectroscopy:

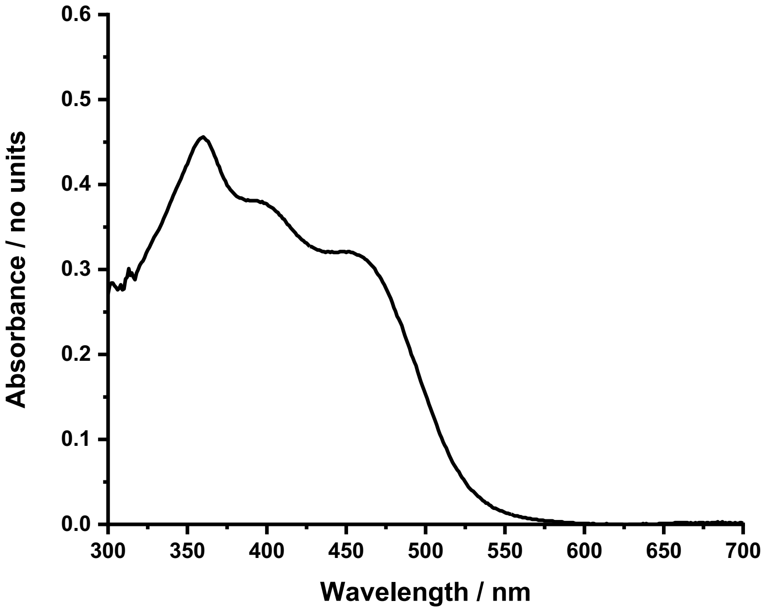

Figure S 10 UV-vis spectrum, 200 μM in 10% DMSO in water.

3. Catalyst-mediated Prodrug Activation Kinetics using HPLC

Reaction kinetics for catalyst-mediated prodrug activation were measured using an HPLC assay, the procedure of which is detailed below, separated into sections labelled: sample conditions, column conditions, calibration curves and kinetic runs. This assay was carried out by taking aliquots of a sample, at specific time intervals during a kinetic run under sample conditions and injecting them onto an HPLC column. The solvent gradient for elution was optimised for the separation of reaction components, which were subsequently quantified using calibration curve data that is obtained for each reaction component by measuring UV-vis absorbance relative to an internal standard. This data was plotted to represent data as conversion as a function of time.

3.1. Sample Conditions

10% DMSO in aqueous buffer (see below) with 500 μM caffeine (internal standard) +/- glutathione (5 mM), at a specified pH at room temperature under aerobic or anaerobic conditions.

Buffer: aqueous MOPS buffer (pH 7.4, 40 mM)

3.2. Column Conditions

Elution: (i) 20-27% acetonitrile in water over 5 min, (ii) 27-99% over 5 min, (iii) 99-20% for 5 min (total run time = 15 min). Both acetonitrile and water mobiles phases contained 0.1% w/v formic acid.

Column: Sunfire reverse phase column.

Oven Temperature: 40 °C.

Injection Volume: 20 μL.

3.3. Calibration Curves

The area under the curve (AUC) for the UV-vis absorbance of moxifloxacin, ***N*-moxi**, and ***C*-moxi** was measured at 290 nm, relative to the internal standard caffeine (500 μM) at room temperature, over a concentration range that contains those expected during kinetic runs. The obtained relative absorbance data (reagent AUC / caffeine AUC) at 290 nm was then plotted against the known reagent concentration.

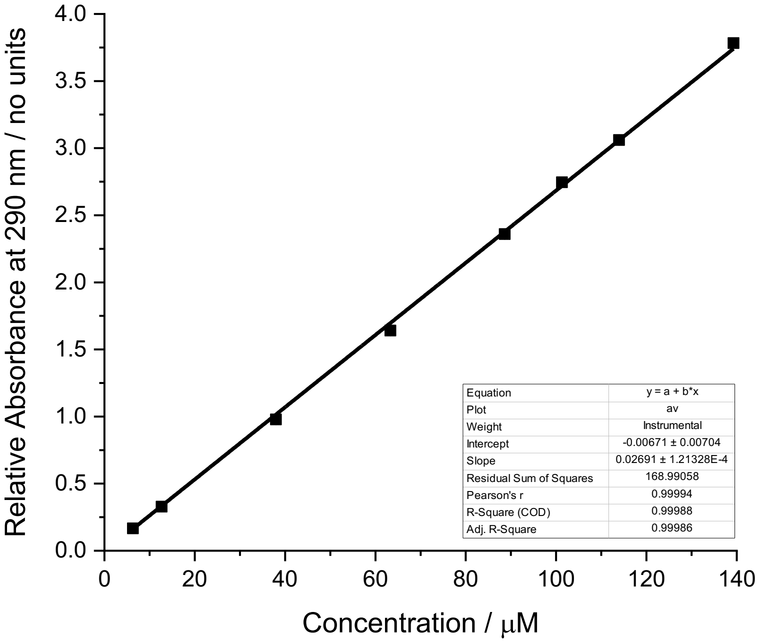

Figure S 11 Calibration curve for moxifloxacin (5 – 110 μM) as relative absorbance at 290 nm to caffeine (500 μM) in aq. MOPS buffer (40 mM) at pH 7.0 at room temperature.

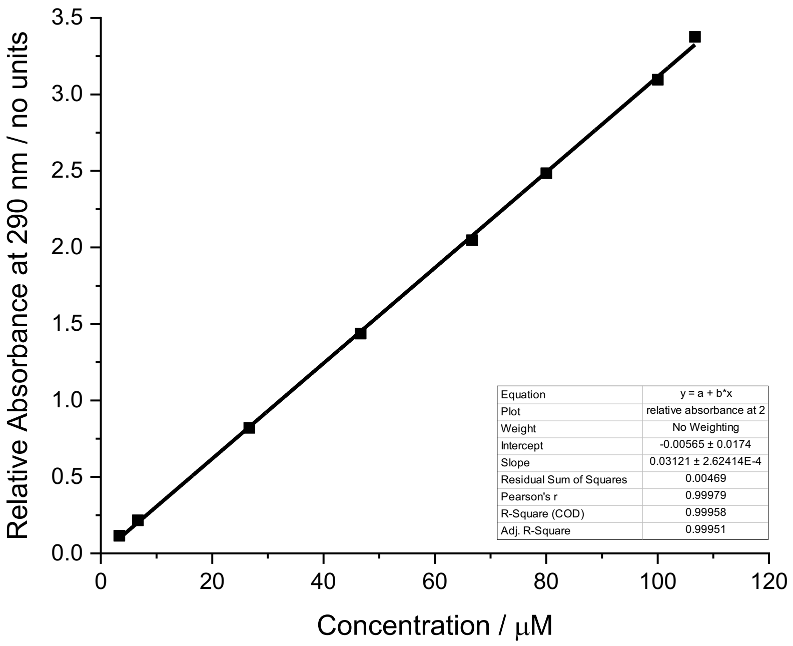

Figure S 12 Calibration curve for ***C*-moxi** (5 – 140 μM) as relative absorbance at 290 nm at caffeine (500 μM) in aq. MOPS buffer (40 mM) at pH 7.0 at room temperature.

3.4. Kinetic Run Procedure

AUC data for each reaction component and caffeine (internal standard) was extracted and converted to conversion % using the relevant calibration curves obtained in section 3.3 as a function of time, in hours. In some cases, prodrug consumption is omitted for clarity. The length of kinetic runs varies depending on the kinetic profile.

3.4.1. Aerobic Conditions

Reaction samples were made up into 2 mL HPLC vials at a volume of 1.6 mL. T = 0 at time of catalyst addition. Prodrug starting concentration was 100 μM. Reaction aliquots for measurement were taken at intervals, which vary depending on the experiment. Experimental triplicates (where relevant) were ran at 15-minute staggered start times, so that injection is taken at the same reaction time. Injection time corresponds to reaction time.

3.4.2. Anaerobic Conditions

Reaction samples were made up in a Schlenk tube under a nitrogen atmosphere at a volume of 3.2 mL in de-gassed solvent. T = 0 at time of catalyst addition. Prodrug starting concentration was 100 μM. Reaction aliquots of 0.2-0.3 mL for measurement were taken at intervals which vary depending on the experiment, from the Schlenk tube and filter transferred into high-recovery HPLC vials. Experimental triplicates (where relevant) were ran at 15-minute staggered start times, so that injection is taken at the same reaction time. Injection time corresponds to reaction time.

3.5. Additional Data for *C*-moxi Activation by Catalyst-Siderophore Catalyst Conjugates

This section includes ***C*-moxi** consumption and corresponding moxifloxacin formation kinetic data for each of the synthesised catalyst-siderophore conjugates and the control catalyst (**Ru-1**) under biologically relevant conditions under an anaerobic atmosphere.

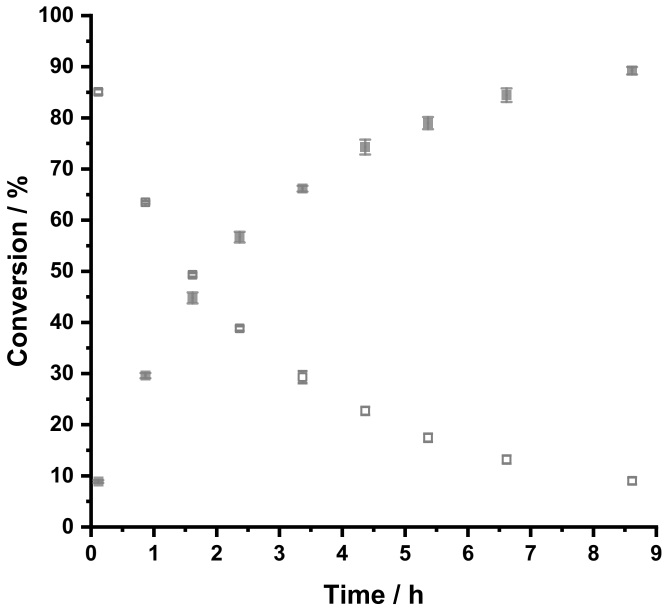

Figure S 13 Catalyst-mediated prodrug activation kinetics in 10% DMSO in aqueous MOPS buffer at pH 7.4 at room temperature under an anaerobic atmosphere, showing ***C*-moxi** (100 μM) consumption (empty squares) and moxifloxacin formation (solid squares) for **Ru-1** at 10 mol % loading.

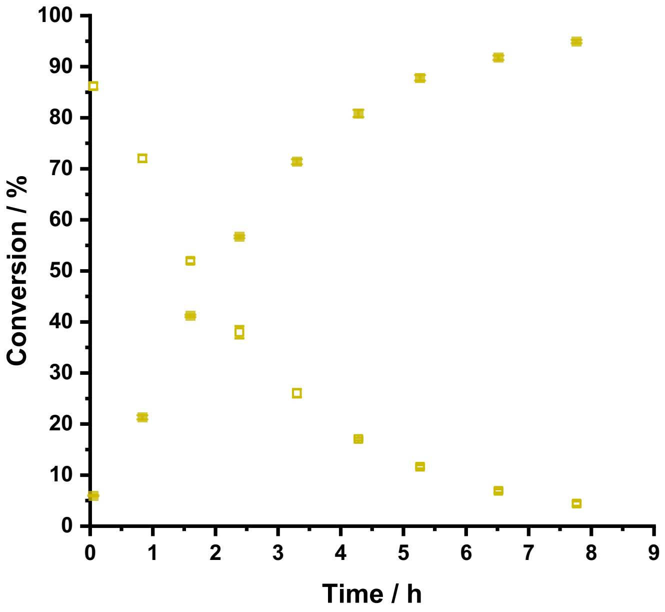

Figure S 14 Catalyst-mediated prodrug activation kinetics in 10% DMSO in aqueous MOPS buffer at pH 7.4 at room temperature under an anaerobic atmosphere, showing ***C*-moxi** (100 μM) consumption (empty squares) and moxifloxacin formation (solid squares) for **Ru-s1** at 10 mol % loading.

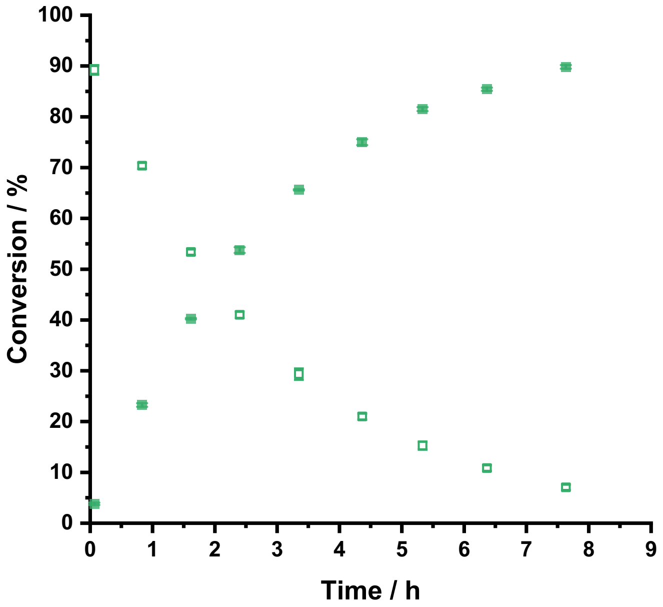

Figure S 15 Catalyst-mediated prodrug activation kinetics in 10% DMSO in aqueous MOPS buffer at pH 7.4 at room temperature under an anaerobic atmosphere, showing ***C*-moxi** (100 μM) consumption (empty squares) and moxifloxacin formation (solid squares) for **Ru-s2** at 10 mol % loading.

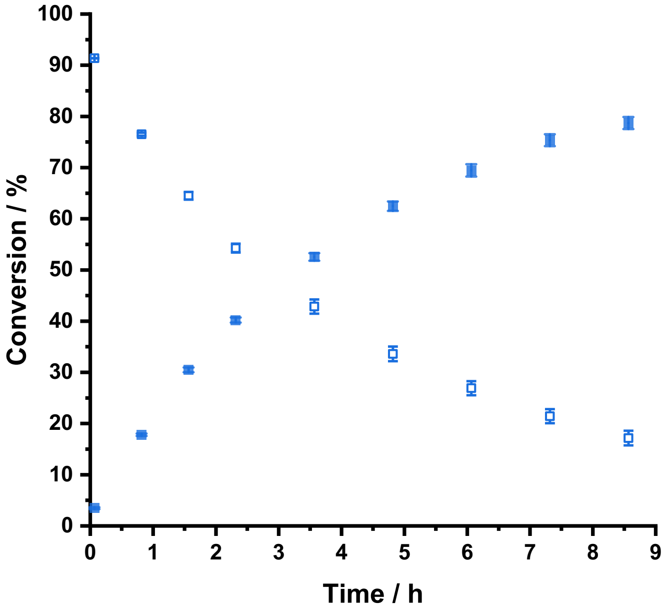

Figure S 16 Catalyst-mediated prodrug activation kinetics in 10% DMSO in aqueous MOPS buffer at pH 7.4 at room temperature under an anaerobic atmosphere, showing ***C*-moxi** (100 μM) consumption (empty squares) and moxifloxacin formation (solid squares) for **Ru-s3** at 10 mol % loading.

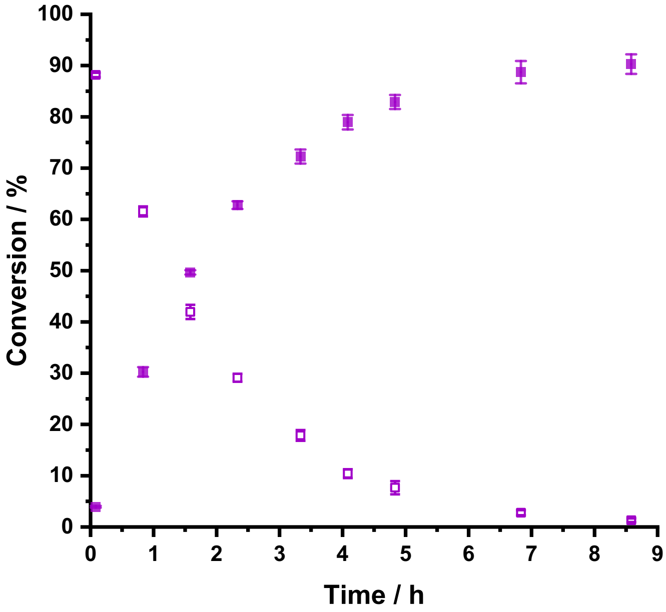

Figure S 17 Catalyst-mediated prodrug activation kinetics in 10% DMSO in aqueous MOPS buffer at pH 7.4 at room temperature under an anaerobic atmosphere, showing ***C*-moxi** (100 μM) consumption (empty squares) and moxifloxacin formation (solid squares) for **Ru-s4** at 10 mol % loading.

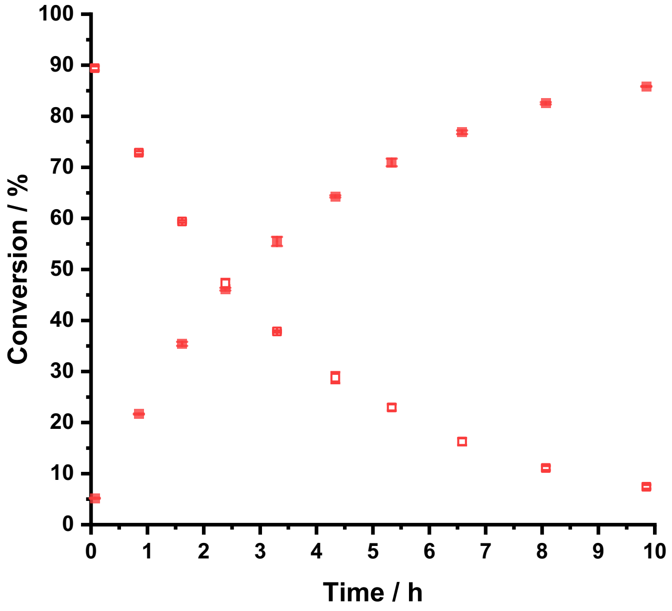

Figure S 18 Catalyst-mediated prodrug activation kinetics in 10% DMSO in aqueous MOPS buffer at pH 7.4 at room temperature under an anaerobic atmosphere, showing ***C*-moxi** (100 µM) consumption (empty squares) and moxifloxacin formation (solid squares) for **Ru-s5** at 10 mol % loading.

4. Catalyst Stability Studies

4.1. Oxygen Sensitivity of the Ru(IV) catalyst Precursor Species

To rule out decomposition of the Ru(IV) catalyst precursor species, **Ru-1** was dissolved in DMSO-*d6* at 10 mM and ^{1}H NMR spectra were recorded at intervals over 2 weeks.

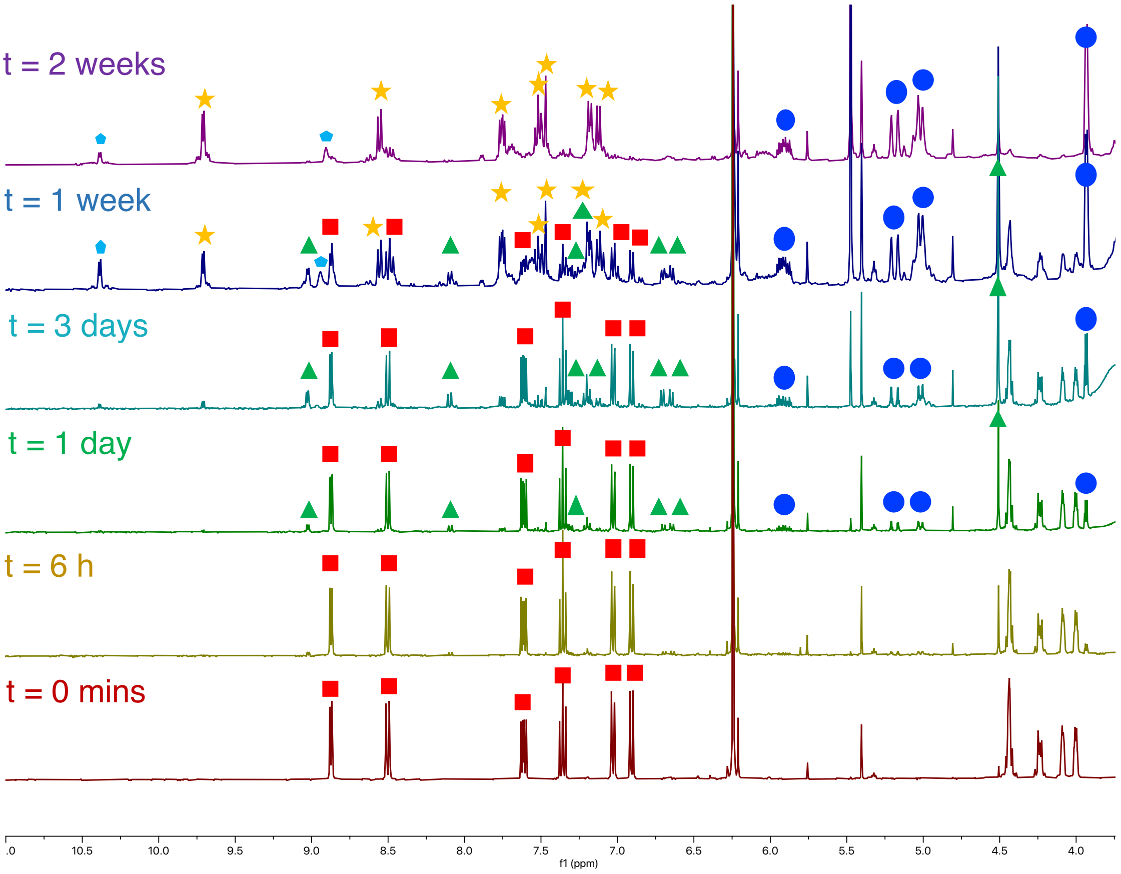

Figure S 19 ^{1}H NMR spectra of **Ru-1** in DMSO-*d6*, after different time points after catalyst addition. **Key:** red square = **Ru-1**, Ru(IV) catalyst precursor species, blue circle = allyl alcohol, green triangle = active Ru(II) intermediate species, cyan pentagon = unknown product 1, yellow star = unknown product 2.

4.2. Oxygen Sensitivity of the Active Ru(II) Intermediate Species

In buffered media or the presence of millimolar concentrations of strong nucleophiles (such as GSH), **Ru-1** is rapidly converted to the active Ru(II) intermediate species. If catalyst decomposition is observed under these conditions at a similar rate to loss of activity displayed in the kinetic assays, this suggests that the active Ru(II) catalyst species is in fact sensitive to molecular oxygen. To test this, **Ru-1** was dissolved at 2 mM in aqueous MOPS buffer (pH 7.4) using deuterated water with 10% DMSO-d_6, where spectra were recorded at t = 15 min and t = 18 h, under an aerobic and anaerobic atmosphere, Figure S 20.

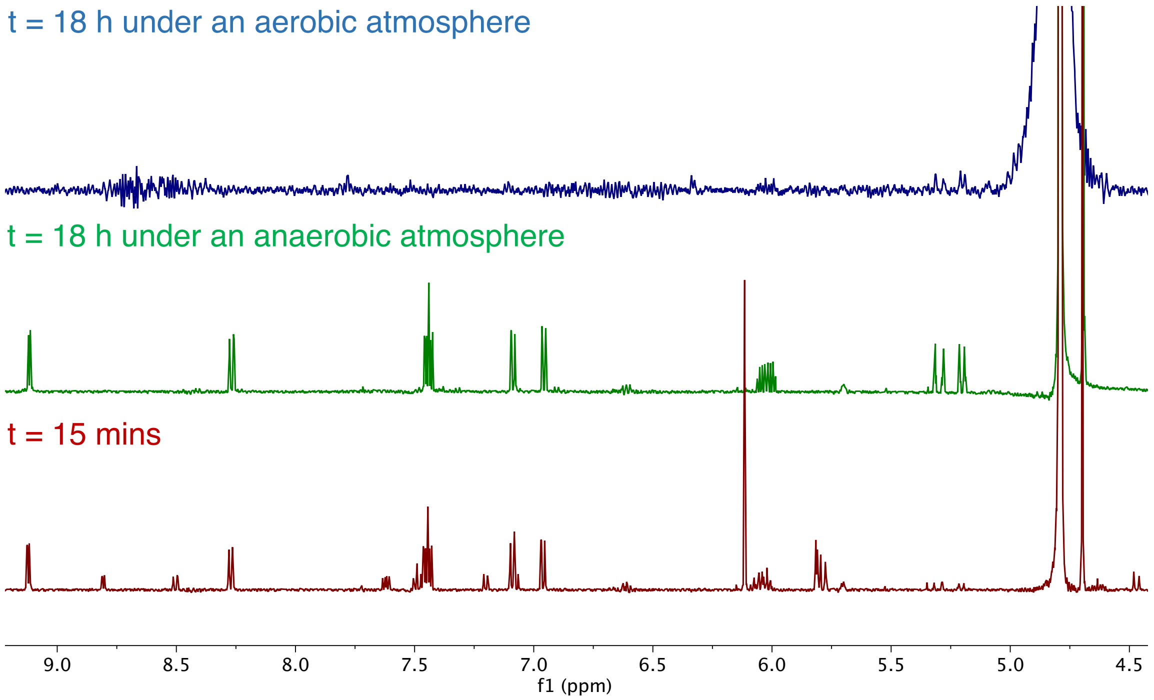

Figure S 20 ^{1}H NMR spectra of **Ru-1** (2 mM) in aqueous MOPS buffer (pH 7.4, 40 mM) using D_2O with 10% DMSO-d_6, at t = 15 min (red) and 18 h under an anaerobic (green) and aerobic (blue) atmosphere. A single ^{1}H NMR spectrum is shown for t = 15 min, as they are identical regardless of atmosphere.

5. Calculated Octanol-Water Coefficients

Calculated octanol-water coefficients at pH 7.4, were calculated using the Marvin Sketch 21.12 plug-in, ChemAxon, with electrolyte concentrations: of 0.1 M Na^+ K^+ and 0.1 M Cl^-.

6. Bacteria Growth Assays

6.1. Media Recipes

Minimal M9 stocks were made from individual stock solutions, using milli-Q pure water and stirred, in 10% w/v Chelex® 100 sodium form, 50-100 mesh (dry) resin (Sigma Aldrich, C7901), for 1 h and then filter sterilised before combining to give the desired media. MHII was made using milli-Q pure water and stirred in 20% w/v Chelex for 24 h and then filter sterilised. MHII was pH adjusted after Chelex treatment (and cation-re-supplementation). Minimal M9 media was not pH adjusted.

All media was prepared and used in either sterile plastics (e.g. falcon tubes) or acid-treated glassware. Acid treatment involved the stirring of 6M hydrochloride in water in glassware for 18 h, followed by decanting, rinsing with acetone, and oven drying.

6.1.1. Minimal M9 Media

The following recipe is reported by Sanderson *et al.*[155]

To make a 10 mL stock of M9 media, was added 7.779 mL milli-Q pure water, 2 mL M9, minimal salts, 5X (Sigma Aldrich, M6030), 200 μL 20% w/v glucose, 20 μL 1M $MgSO_4$ and 1 μL 1M $CaCl_2$. The final concentrations of each component in M9 media are as follows: 48 mM Na_2HPO4, 22 mM KH_2PO_4, 19 mM NH_4Cl, 9 mM NaCl, 22 mM glucose, 2 mM $MgSO_4$ and 0.1 mM $CaCl_2$.

6.1.2. Müller-Hinton II Broth

The following recipe is based on those reported by Ito *et al.*[219]

To make a 200 mL stock of MHII, 200 mL milli-Q pure water was added to 4.4 g Müller Hinton Broth 2 (Sigma Aldrich, 90922). After Chelex treatment, this stock was re-supplemented with 0.4 mM $MgSO_4$ and 0.5 mM $CaCl_2$.

6.2. ICP-MS Measurements for the Iron and Ruthenium Content of Media

ICP-MS measurements were carried out by John Angus using a microwave dilution assay, at the Biorenewables Development Centre, Chessingham Park, Dunnington, York.

Samples for measurement were made up to 2 mL in 15 mL sterile falcon tubes and contained 1% DMSO in the chelexed MHII with 200 μM bpy. Samples made up to determine the iron and ruthenium concentrations of the MHII following the introduction of each synthesised catalyst-siderophore conjugate contained them at 10 μM.

The concentration of ruthenium was also measured by ICP-MS, Table S 1.

Table S 1 Iron content of selected growth media, determined by ICP-MS.

Additive	Additive Concentration / μM	[^{56}Fe] / μM	[^{101}Ru] / μM
**No Addition**	n/a	53	<LLOQ*
**$FeCl_3$**	100	155	<LLOQ*
**Ru-1**	10	19	9
**Ru-s1**	10	33	9
**Ru-s2**	10	22	10
**Ru-s3**	10	33	8
**Ru-s4**	10	43	8
**Ru-s5**	10	56	9

* Lowest limit of quantification = 36.2 ppb; detection limit = 12.05 ppb.

6.3. Bacterial Strains

E. coli K12 (BW25113) is a widely used K12 derivative laboratory strain.

6.4. Iron-starved Bacteria

For all bacteria growth assays, a stock of iron-starved bacteria was grown using the following procedure:

The following procedure is reported by Sanderson *et al.*[11]

E. coli K12 (BW25113) were grown overnight in chelexed M9 supplemented with 10% w/v casamino acids at 37 °C, 180 rpm. Each sample was centrifuged at 3000 g, 4 °C and the solution decanted. The remaining solid was gently re-suspended in chelexed M9 media (10 mL), and centrifuged again, at 3000 g, 4 °C and the solution decanted (x2). The solid was then made to an OD_{600} of 0.05 in chelexed M9 media (20 mL) and grown for 24 h at 37 °C, 180 rpm. This overnight culture was then diluted by adding 0.4 mL to 0.1 mL chelexed glycerol in water and stored at -80 °C. All growth assays that used *E. coli* K12 (BW25113) used these 'iron starved' bacteria stocks.

6.5. Prodrug Uptake Assay

The uptake of the prodrug ***C*-moxi** into *E. coli* K12 (BW25113) was measured utilising an incubation experiment that uses HPLC analysis to quantify the amount of the prodrug that remains in the extracellular environment after its addition to bacteria in their exponential growth, under iron-limited and micro-aerobic conditions. The amount up-taken into bacteria can then be approximated by subtracting this value from the amount added.

The amount of ***C*-moxi** in the extracellular environment was measured using the procedure detailed below, which is separated into sections labelled: incubation preparation, HPLC sample preparation, column conditions, calibration curves and incubation run procedure. This assay works by taking aliquots of a sample, at and injecting them onto a HPLC column which is optimised for the separation of reaction components which are subsequently quantified using calibration curve data that is

obtained for ***C*-moxi** component by measuring UV-vis absorbance relative to an external standard. This data is then plotted to represent data as conversion as a function of time.

6.5.1. Incubation Preparation

Four *E. coli* K12 (BW25113) cultures were prepared in MHII (pH 7.4, total volume 5 mL) overnight at 37 °C with shaking at 180 rpm, in a 50 mL falcon tube, to OD_{600} values in the range of 1.61 – 1.66.

Each overnight culture was diluted to OD_{600} = 0.01 in degassed MHII (pH 7.4) supplemented with 200 μM Bpy, 0.5% DMSO, into the four central wells of a COSTAR 24-well plate (with the surrounding wells filled with degassed water). The plate was covered with a PCR film. These samples were then grown in an Epoch 2 Microplate Spectrophotometer (Biotek) at 37 °C with shaking at 282 rpm, with OD_{600} measurement every 10 min, until an average OD_{600} = 0.1, compared to a 'no cell' control (exponential growth phase).

After this, 1.99 mL aliquots of each sample well were taken into 2 mL Eppendorfs, and to three of them was added 10 μL of ***C*-moxi** (2 mM), to form triplicate. To the remaining sample was added 10 μL of DMSO, as a 'no prodrug' control. These samples were incubated at 37 °C for 15 min.

After incubation, the samples were centrifuged at 3000 g for 10 min at 4 °C, and the supernatant filtered through a 0.2 μm filter where 475 μL was taken for HPLC sample preparation (extracellular sample). The remaining cells were then re-suspended in fresh MHII and also centrifuged at 3000 g, 10 min at 4 °C, and the supernatant filtered through a 0.2 μm filter where 475 μL was taken for HPLC sample preparation (wash sample).

6.5.2. HPLC Sample Preparation

To each of the extracellular and wash samples obtained during incubation, was added 25 μL caffeine in water (10 mM), so that caffeine concentration = 500 μM, including the 'no prodrug' control. Three 'no cell' controls were made up by adding 10 μL ***C*-**

moxi (2 mM) to 1990 μL MHII. A 475 μL aliquot of each was taken, and to it was added 25 μL caffeine in water (10 mM), so that caffeine concentration equalled 500 μM. Each sample was then filtered again through a 0.2 μm filter immediately prior to HPLC

6.5.3. HPLC Column Conditions

Elution: (i) 0-20% acetonitrile in water over 7 min, (ii) 20-80% acetonitrile in water over 5 min, (iii) 80-10% acetonitrile in water over 6 s (iv) 10% acetonitrile in water for 2.5 min (total run time = 15 min). Both acetonitrile and water mobiles phases contained 0.1% w/v formic acid.

Column: Waters Eterra®MS C18 reverse phase column.

Oven Temperature: 40 °C.

Injection Volume: 50 μL.

6.5.4. Calibration Curve

The area under the curve (AUC) for the UV-vis absorbance of ***C*-moxi** was measured at 290 nm, relative to internal standard caffeine (500 μM) at room temperature, over a concentration range that contains those expected during incubation runs. The obtained relative absorbance data (reagent AUC / caffeine AUC) at 290 nm was then plotted against the known ***C*-moxi** concentration.

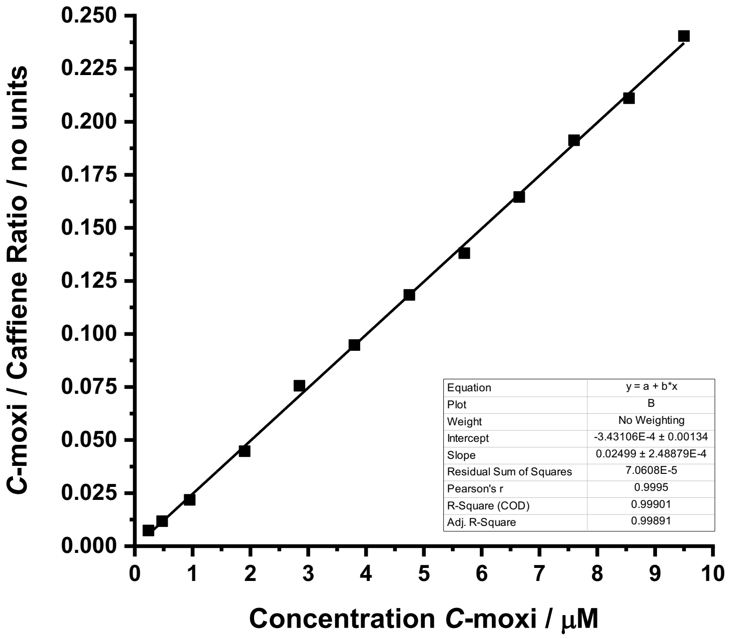

Figure S 21 Calibration curve for ***C*-moxi** (0.3 – 10 µM) as relative absorbance at 290 nm *versus* caffeine (500 µM) in MHII (pH 7.4) at room temperature.

6.5.5. Data Processing

Each of the extracellular and wash samples, including ‘no cell’ controls, were made up and measured in triplicate. Those for the ‘no prodrug addition’ control was made up and measured in singlet. A mean average of the ***C*-moxi** incubated samples and ‘no cell’ controls’ relative AUC to the internal standard caffeine, was calculated. Using the line of best fit acquired in section 6.5.4, ***C*-moxi** concentration for each sample was calculated, and considering the volume of these samples, amount in moles was determined. The percentage ***C*-moxi** in the extracellular and wash samples was calculated by dividing the mean amount (in moles) of ***C*-moxi** by that of the ‘no cell’ control. The percentage up-taken into bacteria was approximated by subtracting the summated percentage of ***C*-moxi** in the extracellular and wash samples from that of the ‘no cell’ control. The molar uptake per OD_{600} was calculated by dividing the approximated molarity inside the bacteria, by the incubation OD_{600} value (0.1).

The bacterial uptake studies results are shown in Table S 2. Unfortunately, bacterial uptake studies were unsuccessful for ***N*-moxi** due to precipitation. It is possible that ***C*-moxi** is in fact stuck on the cell surface. If this is not the case, these data suggests

that *E. coli* K12 (BW25113) bacteria grown under iron-limited, micro-aerobic conditions do indeed uptake ***C*-moxi**, at 16% of the prodrug added at 10 μM. This means that for bacteria grown under these conditions, approximately 20% need activation to observe the maximum inhibitory effect of moxifloxacin.

Table S 2 Estimated bacterial uptake of ***C*-moxi**. This data shows the percentage ***C*-moxi** present in the extracellular and wash samples, relative to the 'no cell' controls.

Extracellular / %	Wash / %	Remainder / %	Uptake / nmol OD_{600}^{-1}
82.7	0.9	16.4	3.31

6.6. Plate Assays

6.6.1. Probing Iron-limitation of Müller-Hinton Broth II

Bacteria Growth

E. coli K12 (BW25113) was grown in MHII (total volume 5 mL), overnight at 37 °C with shaking at 180 rpm, in a 50 mL falcon tube, typically to an OD_{600} ~ 1.7.

Plate Preparation Procedure

Growth assays were carried out in NUNC 96-well plates (Sigma Aldrich, M9410-1CS), with 350 μL wells. To each well was added a total volume of 200 μL. Well conditions were MHII (pH 7.4) with 1% DMSO, with and without 100 μM $FeCl_3$ for iron-supplemented and iron-limited conditions, respectively. For no addition conditions: 150 μL of MHII was added. For iron-limited conditions, no iron: 100 μL of MHII was added, alongside 50 μL 800 μM Bpy in 4% DMSO in MHII. For iron-supplemented, with bpy conditions: 50 μL 800 μM Bpy in 4% DMSO in MHII, 50 μL of MHII was added with 4% DMSO alongside 50 μL 400 μM $FeCl_3$ in MHII. For iron-supplemented, no bpy conditions: 100 μL of MHII was added with 4% DMSO alongside 50 μL 400 μM $FeCl_3$ in MHII. Each well was then inoculated to a starting OD_{600} = 0.01, by adding 50 μL of a 4X concentrated stock of the overnight *E. coli* K12 (BW25113) culture diluted in fresh

MHII, with mixing. Moats and surrounding wells were filled with de-ionised water (2 mL and 200 μL, respectively).

Plate Incubation Procedure

Aerobically grown plates were incubated in an Epoch 2 Microplate Spectrophotometer (Biotek) at 37 °C, shaken continuously in a double orbital pattern (282 rpm). Absorbances at OD_{800} for each well were recorded at t = 0 and every 30 min, for 24 h.

Anaerobically-grown plates were incubated in a FLUOstar Omega microplate reader from BMG LABTECH at 37 °C, shaken continuously in a double orbital pattern (180 rpm) with attached Atmosphere Control Unit (ACU) ensuring a 2% O_2 atmosphere using an attached N_2 gas cylinder. Absorbances at OD_{800} for each well were recorded at t = 0 and every 30 min, for 24 h.

Data Processing

Data was processed in Excel software by first subtracting the average of the no cell controls from all read data. At this point, obvious anomalies were discarded. All data was obtained in at least technical triplicate. For each cell condition, a mean average and error as standard deviation was calculated.

Data is plotted using Origin software, as a growth curve. The mean average absorbance and its corresponding error for each well condition was taken at each time point. This data is shown as absorbance as a function of time.

6.6.2. Antibacterial Activity of Moxifloxacin and Prodrugs

Bacteria Growth

E. coli K12 (BW25113) was grown in MHII (total volume 5 mL), overnight at 37 °C with shaking at 180 rpm, in a 50 mL falcon tube, typically to an OD_{600} ~ 1.7.

Plate Preparation Procedure

Growth assays were carried out in NUNC 96-well plates (Sigma Aldrich, M9410-1CS), with 350 μL wells. To each well was added a total volume of 200 μL. Well conditions

were 200 μM Bpy in MHII (pH 7.4) with 1% DMSO for iron-limited conditions with a starting OD_{600} = 0.01 by dilution of the overnight *E. coli* K12 (BW25113) culture. Substrates were added at 50 μL as X4 stocks in 2% DMSO in MHII. For co-addition experiments, substrates were added at 0.5 μL as X400 stocks in DMSO. Each well was mixed and then the moats and surrounding wells were filled with de-ionised water (2 mL and 200 μL, respectively). Prepared plates were then transported in a sealed container to the plate reader, so that they remained under an anaerobic atmosphere. Moats and surrounding wells were filled with de-ionised water (2 mL and 200 μL, respectively).

Plate Incubation Procedure

Plates were incubated in a FLUOstar Omega microplate reader from BMG LABTECH at 37 °C, shaken continuously in a double orbital pattern (180 rpm) with attached Atmosphere Control Unit (ACU) ensuring a 2% O_2 atmosphere using an attached N_2 gas cylinder. Absorbances at OD_{800} for each well were recorded at t = 0 and every 30 min, for 24 h.

Data Processing

Data was processed in Excel software by first subtracting the average of the no cell controls from all read data. At this point, obvious anomalies were discarded. All data was obtained in at least technical triplicate. For each cell condition, a mean average and error as standard deviation was calculated.

Data is plotted using Origin software, as a dosage response curve. The mean average absorbance at OD_{800} for each well condition was taken at 24 h, along with its corresponding error, and converted to normalised OD_{800} by diving by the mean average growth of the no addition control. This data was then plotted as a function of substrate concentration on a logarithmic scale. A line of best was determined by for each substrate using Origin's default sigmoidal fit analysis.

6.6.3. Antibacterial Activity of Catalyst-Siderophore Conjugates and their Co-addition with Prodrug *C*-moxi

Bacteria Growth

E. coli K12 (BW25113) was grown in MHII (total volume 5 mL), overnight at 37 °C with shaking at 180 rpm, in a 50 mL falcon tube, typically to an OD_{600} ~ 1.7.

Plate Preparation Procedure

Growth assays were carried out in NUNC 96-well plates (Sigma Aldrich, M9410-1CS), with 350 μL wells and prepared in an anaerobic chamber. All stocks were degassed in the chamber at least 3 d prior to their use in assays. To each well was added a total volume of 200 μL. Well conditions were 200 μM Bpy in MHII (pH 7.4) with 1% DMSO for iron-limited conditions with a starting OD_{600} = 0.01 by dilution of the overnight *E. coli* K12 (BW25113) culture. For catalyst-siderophore conjugate antibacterial activity experiments, substrates were added at 1 μL as X200 stocks in DMSO. For co-addition experiments, substrates were added at 0.5 μL as X400 stocks in DMSO. Each well was mixed and then the moats and surrounding wells were filled with de-ionised water (2 mL and 200 μL, respectively). Prepared plates were then transported in a sealed container to the plate reader, so that they remained under an anaerobic atmosphere.

Plate Incubation Procedure

Plates were incubated in a FLUOstar Omega microplate reader from BMG LABTECH at 37 °C, shaken continuously in a double orbital pattern (180 rpm) with attached Atmosphere Control Unit (ACU) ensuring a 2% O_2 atmosphere using an attached N_2 gas cylinder. Absorbances at OD_{800} for each well were recorded at t = 0 and every 30 min, for 24 h.

Data Processing

Data was processed in Excel software by first subtracting the average of the no cell controls from all read data. At this point, obvious anomalies were discarded. All data

was obtained in at least technical triplicate*. For each cell condition, a mean average and error as standard deviation was calculated.

For catalyst-siderophore conjugate antibacterial activity experiments, data is plotted using Origin software, as a dosage response curve. The mean average absorbance at OD_{800} for each well condition was taken at 24 h, along with its corresponding error. This data was then plotted as a function of substrate concentration on a linear scale. The data points are joined by straight lines to depict a general trend.

*Apart from during the antibacterial activity determination of **Ru-1** at 100 nM, **Ru-s2** at 10 μM and 1 μM and **Ru-s3** at 1 μM, which were technical duplicates due to the appearance of obvious anomalies.

For co-addition experiments, data is plotted using Origin software, as a bar chart. The mean average absorbance at OD_{800} for each well condition was taken at 24 h, along with its corresponding error. This data was then plotted with the labelled specific conditions underneath each bar.

6.6.4. Antibacterial Activity of Moxifloxacin and *C*-moxi during the Exponential Growth Phase

Bacteria Growth

E. coli K12 (BW25113) was grown in MHII (total volume 5 mL), overnight at 37 °C with shaking at 180 rpm, in a 50 mL falcon tube, typically to an OD_{600} ~ 1.7.

Plate Preparation 1

Growth assays were carried out in NUNC 96-well plates (Sigma Aldrich, M9410-1CS), with 350 μL wells and prepared in an anaerobic chamber. All stocks were degassed in the chamber at least 3 d prior to their use in assays. To each well was added a total volume of 199 μL. Well conditions were 200 μM Bpy in MHII (pH 7.4) with 1% DMSO for iron-limited conditions with a starting OD_{600} = 0.01 by dilution of the overnight *E. coli* K12 (BW25113) culture. Each well was mixed and then the moats and surrounding wells were filled with de-ionised water (2 mL and 200 μL, respectively). Prepared

plates were the covered with a PCR film and their lid, then transported in a sealed container to the plate reader, so that they remained under an anaerobic atmosphere.

Plate Incubation Procedure 1

Plates were incubated in an Epoch 2 Microplate Spectrophotometer (Biotek) at 37 °C, with no shaking, until absorbances OD_{600} reached values 0.1 greater than the average of 'no cell' controls (exponential growth phase). These plates were then transported back to the anaerobic chamber.

Plate Preparation 2

The PCR film was removed and to each well was added substrate at 1 μL as a X200 concentrated stock in DMSO with mixing. Prepared plates were then transported in a sealed container to the plate reader, so that they remained under an anaerobic atmosphere.

Plate Incubation Procedure 2

Plates were incubated in a FLUOstar Omega microplate reader from BMG LABTECH at 37 °C, shaken continuously in a double orbital pattern (180 rpm) with attached Atmosphere Control Unit (ACU) ensuring a 2% O_2 atmosphere using an attached N_2 gas cylinder. Absorbances at OD_{800} for each well were recorded at t = 0 and every 30 min, for 18 h.

Data Processing

Data was processed in Excel software by first subtracting the average of the no cell controls from all read data. At this point, obvious anomalies were discarded. All data was obtained in at least technical triplicate. For each cell condition, a mean average and error as standard deviation was calculated.

Data is plotted using Origin software, as a dosage response curve. The mean average absorbance at OD_{800} for each well condition was taken at 18 h, along with its corresponding error, and converted to normalised OD_{800} by diving by the mean average growth of the no addition control. This data was then plotted as a function of

substrate concentration on a logarithmic scale. A line of best was determined by for each substrate using Origin's default sigmoidal fit analysis.

The MIC data were obtained for ***C*-moxi** and moxifloxacin after addition during the exponential growth phase, represented as dosage response curves, Figure S 22.

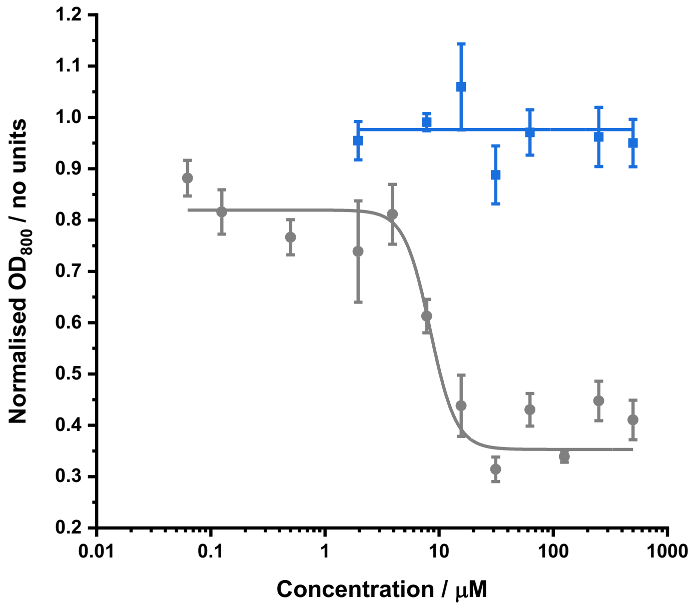

Figure S 22 Dosage-response curves of *E. coli* K12 (BW25113) overall growth, 18 h after substrate addition during the exponential growth phase. Data are normalised to 'no addition' controls, for each of moxifloxacin (grey) and ***C*-moxi** (blue), at their varied substrate concentrations under iron-limited (MHII supplemented with 200 µM bpy), micro-aerobic (2% O_2) conditions.

6.6.5. Antibacterial Activity of Catalyst-Siderophore Conjugates following Bacteria Incubation with *C*-moxi During the Exponential Growth Phase

Bacteria Growth

E. coli K12 (BW25113) was grown in MHII (total volume 5 mL), overnight at 37 °C with shaking at 180 rpm, in a 50 mL falcon tube, typically to an OD_{600} ~ 1.7.

Plate Preparation 1

Growth assays were carried out in COSTAR 24-well plates (Sigma Aldrich, CLS3527), with 3.4 mL wells and prepared in an anaerobic chamber. All stocks were degassed in the chamber at least 3 d prior to their use in assays. To each well was added a total volume of 2.5 mL. Well conditions were 200 μM Bpy in MHII (pH 7.4) with 1% DMSO for iron-limited conditions with a starting OD_{600} = 0.01 by dilution of the overnight *E. coli* K12 (BW25113) culture. Each well was mixed and then the moats and surrounding wells were filled with de-ionised water (2.5 mL). Prepared plates were the covered with a PCR film and their lid, then transported in a sealed container to the plate reader, so that they remained under an anaerobic atmosphere.

Plate Incubation Procedure 1

Plates were incubated in an Epoch 2 Microplate Spectrophotometer (Biotek) at 37 °C, with no shaking, until absorbances OD_{600} reached values 0.1 greater than the average of 'no cell' controls (= exponential growth). These plates were then transported back to the anaerobic chamber.

Spin Down and Washing Procedure

A volume of 1.99 mL was taken from each well into a 2 mL Eppendorf and to it was added was added either 10 μL of a X200 concentrated ***C*-moxi** stock in DMSO (for ***C*-moxi** incubation), or plain DMSO (for non-***C*-moxi** incubation controls), with mixing. These Eppendorfs were placed into a sealed container and transferred to the 37 °C room and shaken for 15 min at 180 rpm. After this time, the Eppendorfs were spun down (3000g, 10 min, 4 °C), the pellet isolated and then re-suspended in 2 mL of 200 μM Bpy in MHII (pH 7.4) with 1% DMSO (x3).

Plate Preparation 2

Subsequent growth assays were carried out in NUNC 96-well plates (Sigma Aldrich, M9410-1CS), with 350 μL wells and prepared in an anaerobic chamber. To each well was added a total volume of 199 μL from the aforementioned stocks prepared during the 'Spin Down and Washing Procedure'. Well conditions were 200 μM Bpy in MHII

(pH 7.4) with 1% DMSO for iron-limited conditions with a starting OD_{600} = 0.1. Substrates were added at 1 μL as X200 stocks in DMSO. Each well was mixed and then the moats and surrounding wells were filled with de-ionised water (2 mL and 200 μL, respectively). Prepared plates were transported in a sealed container to the plate reader, so that they remained under an anaerobic atmosphere.

Plate Incubation Procedure 2

Plates were incubated in a FLUOstar Omega microplate reader from BMG LABTECH at 37 °C, shaken continuously in a double orbital pattern (180 rpm) with attached Atmosphere Control Unit (ACU) ensuring a 2% O_2 atmosphere using an attached N_2 gas cylinder. Absorbances at OD_{800} for each well were recorded at t = 0 and every 30 min, for 18 h.

Data Processing

Data was processed in Excel software by first subtracting the average of the no cell controls from all read data. At this point, obvious anomalies were discarded. Incubations were employed in technical triplicate, and subsequent data for each in at least technical duplicate. For each cell condition, a mean average and error as standard deviation was calculated.

Data is plotted using Origin software, as a bar chart. The mean average absorbance at OD_{800} for each well condition was taken at 18 h, along with its corresponding error. This data was then plotted with the labelled specific conditions underneath each bar. The difference between incubations (OD_{800} for substrate following ***C*-moxi** incubation – OD_{800} for substrate following DMSO incubation), is shown above the corresponding bars in the bar chart.

7. Cytotoxicity against Mammalian Cells

7.1. Cell viability assays for Moxi, *C*-Moxi and Ru-s4

Dulbecco's Modified Eagle's Medium (DMEM: [+] 4.5 g/L D-Glucose, L-Glutamine; [-] Pyruvate), containing 10% fetal calf serum, 1% penicillin and streptomycin, was used as growth medium for HeLa cells. DMEM ([+] 1 g/L D-Glucose; [+] Pyruvate), containing 10% fetal calf serum, 1% penicillin and streptomycin, was respectively used for GM5657 cells. HeLa and GM5657 cells were detached from the wells with trypsin and EDTA, harvested by centrifugation and resuspended in the respective cell culture medium. The assays were carried out on 96 well plates with a density of 6000 cells per well. After 24 h of incubation at 37°C and 10% CO_2, the cells were treated with **Moxi**, ***C*-Moxi** and **Ru-s4** (constant DMSO concentrations of 0.5%) with a final volume of 200 μL per well. For a negative control, one series of cells were only treated with 0.5% DMSO, and for a positive control, one series was treated with cisplatin. After 48 h of incubation, 50 μL PBS containing MTT (2.5 mg/mL) was added. The cells were incubated for an additional 2 h. Afterwards, the medium was removed, and 200 μL of DMSO were added. The formazan crystal absorption was measured at 550 nm, using a reference wavelength of 650 nm. Each assay was carried out in triplicates (three independent experiments) for each cell line, Table S3.

Table S 3 Results for the cell viability assays of **Moxi**, ***C*-Moxi** and **Ru-s4** with cisplatin as control. MTT assay, 48 h incubation time, IC_{50} values are given in μM.

**Compound**	**Cisplatin**		**Moxi**		***C*-Moxi**		**Ru-s4**	
**Cell line**	HeLa	GM5657	HeLa	GM5657	HeLa	GM5657	HeLa	GM5657
**Run 1**	12.6	19.5	>100	>100	>100	>100	>100	>100
**Run 2**	20.6	18.0	>100	>100	>100	>100	>100	>100
**Run 3**	17.8	18.9	>100	>100	>100	>100	>100	>100
**Mean**	17.0	18.8	>100	>100	>100	>100	>100	>100
**Standard Error**	2.3	0.4	-	-	-	-	-	-

8. References

(1) Houghton, T. J.; Tanaka, K. S. E.; Kang, T.; Dietrich, E.; Lafontaine, Y.; Delorme, D.; Ferreira, S. S.; Viens, F.; Arhin, F. F.; Sarmiento, I.; et al. *J. Med. Chem.* **2008**, *51* (21), 6955-6969. DOI: 10.1021/jm801007z.

(2) Tanaka, K. S. E.; Houghton, T. J.; Kang, T.; Dietrich, E.; Delorme, D.; Ferreira, S. S.; Caron, L.; Viens, F.; Arhin, F. F.; Sarmiento, I.; et al. *Bioorg. Med. Chem.* **2008**, *16* (20), 9217-9229. DOI: https://doi.org/10.1016/j.bmc.2008.09.010.

(3) Völker, T.; Meggers, E. *ChemBioChem* **2017**, *18* (12), 1083-1086. DOI: 10.1002/cbic.201700168.

(4) Bergeron, R. J.; McGovern, K. A.; Channing, M. A.; Burton, P. S. *J. Org. Chem.* **1980**, *45* (9), 1589-1592.

(5) Barden, T. C.; Buckwalter, B. L.; Testa, R. T.; Petersen, P. J.; Lee, V. J. *J. Med. Chem.* **1994**, *37* (20), 3205-3211.

(6) Sleath, P. R.; Noar, J. B.; Eberlein, G. A.; Bruice, T. C. *J. Am. Chem. Soc.* **1985**, *107* (11), 3328-3338.

(7) Ernst, G.; Akuma, D.; Au, V.; Buchler, I. P.; Byers, S.; Carr, G. V.; Defays, S.; de León, P.; Demaude, T.; DePasquale, M.; et al. *ACS Med. Chem. Lett.* **2019**, *10* (11), 1573-1578. DOI: 10.1021/acsmedchemlett.9b00345.

(8) Brown, M. F.; Mitton-Fry, M. J.; Arcari, J. T.; Barham, R.; Casavant, J.; Gerstenberger, B. S.; Han, S.; Hardink, J. R.; Harris, T. M.; Hoang, T.; et al. *J. Med. Chem.* **2013**, *56* (13), 5541-5552, Article. DOI: 10.1021/jm400560z.

(9) Tarapdar, A.; Norris, J. K. S.; Sampson, O.; Mukamolova, G.; Hodgkinson, J. T. *Beilstein J. Org. Chem.* **2018**, *14* (1), 2646-2650.

(10) Rudd, S. E.; Roselt, P.; Cullinane, C.; Hicks, R. J.; Donnelly, P. S. *Chem. Commun.* **2016**, *52* (80), 11889-11892, 10.1039/C6CC05961A. DOI: 10.1039/C6CC05961A.

(11) Sanderson, T. J.; Black, C. M.; Southwell, J. W.; Wilde, E. J.; Pandey, A.; Herman, R.; Thomas, G. H.; Boros, E.; Duhme-Klair, A.-K.; Routledge, A. *ACS Infect. Dis.* **2020**, *6* (9), 2532-2541. DOI: 10.1021/acsinfecdis.0c00568.

(12) Ito, A.; Nishikawa, T.; Matsumoto, S.; Yoshizawa, H.; Sato, T.; Nakamura, R.; Tsuji, M.; Yamano, Y. *Antimicrob. Agents Chemother.* **2016**, *60* (12), 7396-7401.
